# Major Intrinsic Proteins in Fungi: A Special Emphasis on the XIP Subfamily

**DOI:** 10.3390/jof11070543

**Published:** 2025-07-21

**Authors:** Jean-Stéphane Venisse, Gisèle Bronner, Mouadh Saadaoui, Patricia Roeckel-Drevet, Mohamed Faize, Boris Fumanal

**Affiliations:** 1 University Clermont Auvergne, INRAE, PIAF, 63000 Clermont-Ferrand, France; l54860435@hotmail.com (M.S.); patricia.drevet@uca.fr (P.R.-D.); boris.fumanal@uca.fr (B.F.); 2 CNRS, Laboratoire Microorganismes: Génome et Environnement, University Clermont Auvergne, 63000 Clermont-Ferrand, France; gisele.bronner@uca.fr; 3 Laboratory of Plant Biotechnology, Ecology and Ecosystem Valorization CNRST-URL10, Faculty of Sciences, University Chouaib Doukkali, El Jadida 24000, Morocco; faizemohamed@yahoo.fr

**Keywords:** aquaporin, aquaglyceroporin, *X*-intrinsic protein (XIP), evolution, deep divergence, phylogeny, 3D modeling

## Abstract

The fungal kingdom, with an estimated five million species, has undergone extensive diversification over the past billion years and now occupies a wide array of ecological niches from terrestrial to aquatic ecosystems. To thrive in such diverse environments, fungi must exhibit finely tuned physiological and morphological responses orchestrated by conserved molecular pathways. Increasing evidence suggests that aquaporins (AQPs) play a key role in mediating these adaptive responses, particularly under varying abiotic and biotic stress conditions. However, despite notable advances in recent decades, the precise functional roles of AQPs within the fungal kingdom remains largely unresolved in the field of cell biology. AQPs are transmembrane proteins belonging to the major intrinsic proteins (MIPs) superfamily, which is characterized by remarkable sequence and structural diversity. Beyond their established function in facilitating water transport, MIPs mediated the bidirectional diffusion of a range of small inorganic and organic solutes, ions, and gases across cellular membranes. In fungi, MIPs are classified into three main subfamilies: orthodox (i.e., classical) AQPs, aquaglyceroporins (AQGP), and *X*-intrinsic proteins (XIPs). This review provides a concise summary of the fundamental structural and functional characteristics of fungal aquaporins, including their structure, classification, and known physiological roles. While the majority of the current literature has focused on the aquaporin and aquaglyceroporin subfamilies, this review also aims to offer a comprehensive and original overview of the relatively understudied *X*-intrinsic protein subfamily, highlighting its potential implication in fungal biology.

## 1. General Considerations on MIPs

Membranes, whether plasmamembranes, endomembranes, or those delimiting organelles such as chloroplasts or mitochondria, serve as semi-permeable barriers that separate various cellular systems (cytosol and organelles) from their respective external environments (apoplasm or cytosol). Due to their hydrophobic nature, membranes establish the physical and chemical boundaries of cell or subcellular structures within water-soluble environments. Consequently, these systems must rely on a vast and highly complex network of transmembrane protein transporters to regulate the controlled influx and efflux of polar molecules and ions. The maintenance of distinct organic and hydro-mineral compositions between the extracellular fluid and the cytosol is essential for cellular stability and survival. In this context, aquaporins (AQPs) figure as major actors [1].

AQPs are members of the major intrinsic proteins (MIPs) superfamily and facilitate the passive movement of water and small, neutral solutes across biological membranes. They belong to an ancient family of water channel proteins that are found across all kingdoms of life (Archaea, Bacteria, Fungi, Plantae, Animalia, Protozoa, Chromista, and virus), underscoring their essential role in a plethora of fundamental biological processes [2,3]. The discovery of aquaporins has provided valuable insights into the molecular mechanisms underlying solute transmembrane transport, encompassing both natural and synthetic substances (e.g., drugs).

**Figure 1:**
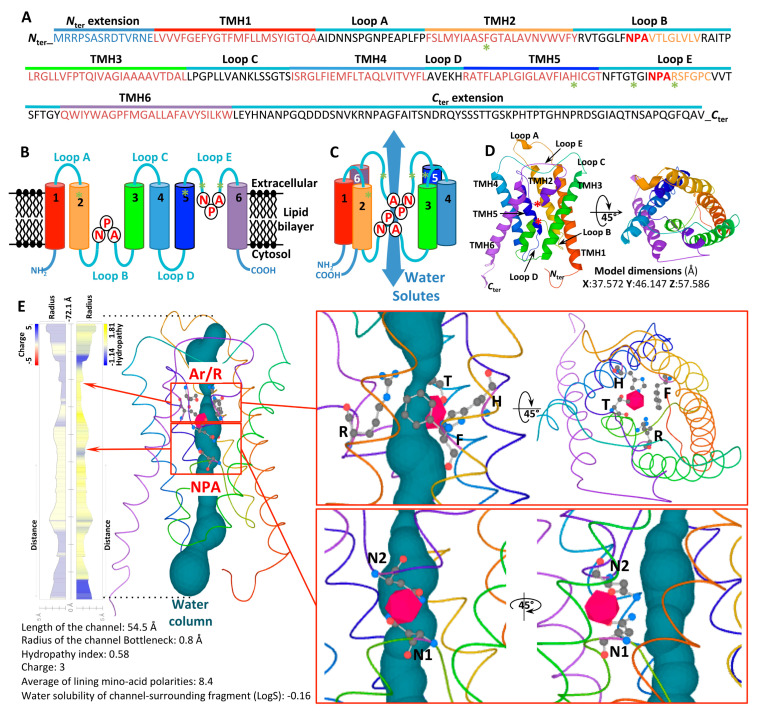
Structure of the AQP protomer (Orthodox AQP; *Triat*6990) from *Trichoderma atroviride*. (**A**) Primary structure of *Triat*6990. TMH, transmembrane α-helices domain. The green asterisks correspond to the amino acid tetrad constituting the Ar/R domain. In orange are shown the two pseudo TMHs (LE and LB), each including the NPA motif. (**B**) Schematic representation of the secondary structures. (**C**) Schematic representation of the folding of the six secondary structures (TMH1–TMH6) and the five electrostatic loops (Loops A–E) into a tertiary structure, an aquaporin protomer. (**D**) Predicted 3D models of the protomer, in both lateral (**left**) and top views (apoplastic side, **right**). Models were generated by using the PHYRE2.0 Protein Fold Recognition server, using the Normal mode modeling based on alignment to experimentally solved protein structures. The modeling of *Triat*AQP-6990 was performed by comparing it with the c5bn2A template (AQY1 from *Saccharomycas cerevisiae*), with 241 residues (i.e., 80% of the sequence) modeled with 100% confidence. X, Y, and Z model dimensions were expressed in Angströms (Å). The two NPA motifs are indicated by red asterisks. (**E**) Physicochemical properties (radius, charge, and hydrophobicity) of *Triat*6990 pore structure and details of the amino acid residues involved in the Ar/R (F64, H184, T193, and Arg199) and the two NPA motifs and their positions within the protomer. Only the Arginines (N1 from NPA1 in Loop A, and N2 from NPA2 in Loop E), which are positioned antiparallel and involved in the selectivity and inversion of each water molecule as it passes through the channel, are depicted. The NPA motifs fold into half-membrane-spanning helices that create a dipole moment and prevent proton (H^+^) entry into the cell. The Ar/R selectivity filters act as size-exclusion filters for water and small molecules. Each schema and model is visualized by rainbow colors from *N* to *C* termini. The 3D model, physicochemical properties, and the predicted water column were calculated using Mole 2.5 software.

Structurally, AQP proteins typically assemble into homotetrameric and heterotetrameric arrangements, with each protomer acting as an independent pore [4,5]. These tetrameric configurations are regarded as a crucial regulatory mechanism for the permeability of each AQP protomer [6]. Each protomer is composed of six transmembrane (TM) α-helices (H1–H6), connected by five loops (denoted LA through LE), with both the *N*- and *C*-terminal regions localized in the cytosol, as presented for the fungus *Trichoderma atroviride* in Figure 1. The helices are symmetrically organized into two vestibules, forming a central conduction pore that governs transport activity. The pore spans approximately 55 Å in height, from the intra- to the extra-cellular space. The three-dimensional arrangement of these secondary structure elements thus imparts the characteristic hourglass shape to aquaporins.

Inside the channel, solute permeation is facilitated by a series of polar interactions between the solute molecules and specific amino acids, whose side chains are embedded within the central channel. Two primary constriction sites within the pore are involved in its selectivity. The LB and LE loops each contain a short α-helix, forming a seventh pseudo-transmembrane helix, and are connected by highly conserved Asparagine-Proline-Alanine (NPA) motifs [7]. These loops are partially located within the membrane. The asparagine (N) side chains, oriented toward the pore’s interior, are situated at the ends of two half-helices, and their antiparallel dipoles create an electrostatic barrier in this region, which is essential for proton exclusion.

A second structural feature common to aquaporins is the aromatic/arginine (ar/R) constriction residue quartet [8,9]. This quartet comprises four amino acid residues arranged within the same spatial plane, with two residues located in the H2 and H5 helices and two in loop E (LE1 and LE2). Forming the narrowest part of the pore, this constriction plays a crucial role in the substrate selectivity. It acts as a size-exclusion barrier, blocking the transport of bulkier substrates, while simultaneously providing key hydrogen bonds and van der Waals contacts that stabilize the solutes and/or water molecules being transported [8,10]. In this regard, the selectivity filter residues of water-specific aquaporins typically form a smaller (~2.8 Å) and more hydrophilic pore, whereas those in glycerol transporters form a larger (~3.4 Å) and more hydrophobic pore [11,12].

Natural substitutions within the ar/R selectivity filter and NPA motifs are believed to play a critical role in determining the broad substrate specificity of AQPs. Additionally, residues surrounding these key sites may vary, substantially influencing the overall size and hydrophobicity of the pore. Molecules transported through aquaporins pass via a network of hydrogen bonds, which are formed with the carbonyl groups of the B and E loops, as well as with the side chains of specific residues. While the aforementioned motifs are highly conserved across all aquaporins, their local chemical environment—defined by the chemical groups of amino acids that are spatially proximal, although not necessarily adjacent in the linear protein sequence—determines the selectivity and efficiency of solute transport. These atomic interactions and chemical groups underpin the functional variability of aquaporins, which, in part, explains the complexity and multifactorial nature of AQP gating, as observed in various cell systems [13,14].

In addition, various motifs involved in co- and post-translational modifications (such as *N*-glycosylation, deamidation, *N*-terminal acetylation, methylation, ubiquitination, and phosphorylation), along with external factors such as pH, temperature, solute gradients, pressure, and membrane tension, all contribute to shaping the functional core of the channels. These factors, along with trafficking processes (where AQPs are transported from intracellular storage sites to the plasma membrane) and interactions with protein or minerals partners (e.g., cadmium, calcium, etc.), govern the selective transport of water, neutral solutes, ions, small uncharged molecules, and gases across cellular membranes. Notably, structural variations in the α-helices, loops, and the *N*- and *C*-termini of AQPs appear to confer substantial functional diversity [15].

Thus, two “prototype” members of the MIP superfamily can be distinguished: “orthodox” or “classical” aquaporins (i.e., aquaporins, stricto sensu), which mediate the rapid and selective flux of water across biological membranes and play important roles in the osmoregulation of cells and organisms, and aquaglyceroporins (AQGPs or AGPs), which facilitate the transmembrane transport of small uncharged molecules such as polyols, H_2_O_2_, urea, arsenite, and others, in addition to or independent of water (depending on the specific member). These AQGPs are involved in osmoregulation, nutrient uptake, and potentially other physiological processes. However, within both groups, a significant diversity of members and dedicated subfamilies exists. In animals, aquaporins are categorized into four major groups: Aqp4-like classical aquaporins (Aqp), Aqp8-like aqua-ammoniaporin, Aqp12-like unorthodox aquaporins, and Glp aquaglyceroporins [15]. In *Viridiplantae*, based on sequence homology and potential subcellular localization, the diversity of MIPs is organized into eight subfamilies. Four of these subfamilies are well characterized: PIP (Plasma Membrane Intrinsic Protein), TIP (Tonoplast Intrinsic Protein), NIP (Nodulin 26-like Intrinsic Protein), and SIP (Small and Basic Intrinsic Protein) [16,17]. A fifth subfamily, XIP (Uncharacterized *X*-Intrinsic Protein), was identified in some bryophytes and dicots [18,19]. Three additional subfamilies—GIP (GlpF-Like Intrinsic Protein), HIP (Hybrid Intrinsic Protein), and LIP (Large Intrinsic Proteins)—have been described in more basal plant groups. GIP and HIP were identified in the bryophyte *Physcomitrella patens* [18] and the lycophyte *Selaginella moellendorffii* [20], respectively, while LIP has been found exclusively in some algae, such as the Ochrophytes [21]. In bacteria, approximately 10% of studied genomes encode MIPs, which fall into two groups: AQP and AQGP (or Glp) [2,22]. However, the increasing power of phylogenetic models and the availability of annotated genomes from various taxa are revealing a complex evolutionary history of both Archaeal and bacterial aquaporins, categorized into four main types: AqpZ, AqpN, AqpM, and GlpF [15,23]. Finally, as the focus of this special issue, fungal MIPs have been classified into three major subfamilies: the fungal “orthodox” aquaporins (AQPs), the fungal aquaglyceroporins (AQGPs), and the fungal uncategorized (*X*)-intrinsic proteins (XIPs) [23,24,25].

Although AQPs are remarkably conserved in their core structural architecture and function as permeases for a variety of solutes essential for life, they paradoxically exhibit considerable diversity in the residues that shape them. Numerous future studies will be required to support and refine our understanding of the evolutionary dynamics of AQPs across the major clades of life. However, it is already striking that the evolution of most major AQPs groups closely correlates with the diversification of the biosphere, as reflected in the tree of life. There are, however, notable exceptions, such as the XIPs, which appear to be shared exclusively by a few plant and fungal groups. This subfamily warrants particular attention as its original nomenclature suggests that it remains poorly studied and the diversity of its members is still largely unexplored.

## 2. MIP Fungal Diversity and Function

**Figure 2:**
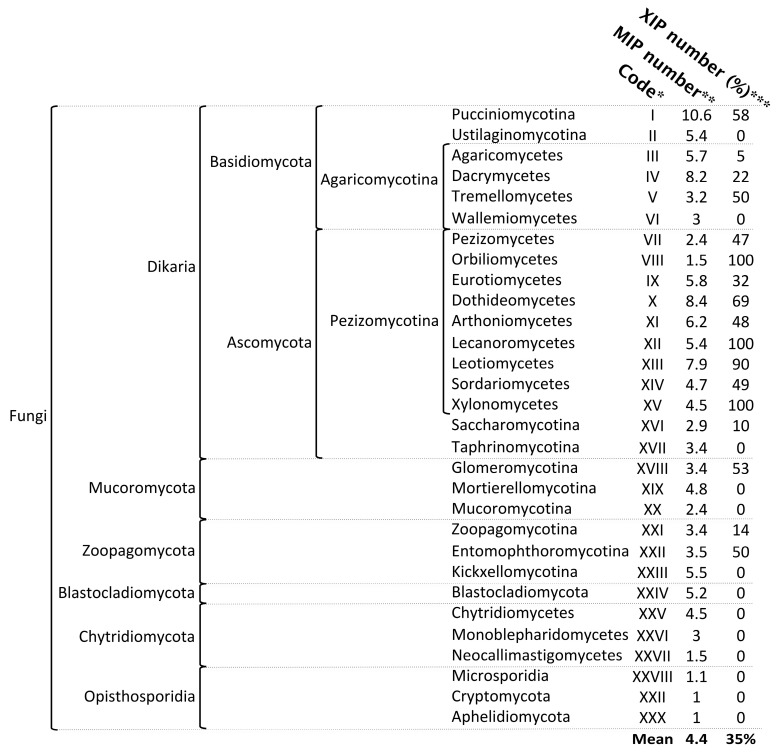
Diversity and distribution of fungal aquaporins within the major phyla of the fungal kingdom. * Code assigned to each phylum and used in the phylogenetic analyses for this study. ** Average number of aquaporins, without subfamily discrimination. *** Percentage of species sharing the XIP subfamily. The distribution of major phyla was derived from the data displayed in JGI’s MycoCosm portal. The phyla *Arthonomycetes* and *Aphelidiomycota* were added. All the data were extracted from the information available on the JGI public platform.

Fungi constitute one of the five kingdoms of the Eukaryota life domain and are likely the most species-rich group of eukaryotic organisms after insects [26]. A recent reassessment of global fungal diversity suggests that the total number of fungal species may range from 2.2 to 5 million worldwide [27]. According to widely accepted classifications and alternative simplifications [28,29,30,31], more than 200 fungal orders are classified into 19 phyla, which are divided into six major groups: the subkingdoms *Dikarya* (comprising the phyla Ascomycota and *Basidiomycota*) and *Chytridiomyceta* (comprising the phyla *Chytridiomycota*, *Monoblepharidomycota*, and Neocallimastigomycota); the phyla *Mucoromycota*, *Zoopagomycota*, and *Blastocladiomycota*; and the major group *Opisthosporidia* (comprising the phyla *Aphelidiomycota*, *Cryptomycota*/*Rozellomycota*, and *Microsporidia*) (Figure 2). Among the relatively small fraction of fungal species currently described, 2683 annotated/reference genomes are available through the JGI’s MicoCosm portal [32], and 5028 are available through the NCBI portal, covering the major phyla that structure the fungal kingdom (April 2025).

In contrast to the remarkable diversity of fungal species evolving in extraordinarily varied biotopes, the diversity of fungal MIPs appears to be more limited than that observed in animals and plants. Fungal genomes typically contain an average of 4.4 MIP members, with a range of 1 to 20 (Figure 2). Notably, the highest numbers of MIP members are found in four clades of filamentous fungi: two in *Basidiomycota* (*Pucciniomycotina* and *Dacrymycetes*) and two in Ascomycota (*Dothideomycetes* and *Leotiomycetes*). Basal fungal lineages (according to the classification of [31]) exhibit a low number of MIPs, with some species possessing only a single member: *Aphelidiomycota*, *Cryptomycota*, *Microsporidia*, and *Neocallimastigomycetes*. However, it remains challenging to determine whether their single member should be classified as an AQP or an AQGP based solely on sequence alignment, phylogeny analysis, and the presence of certain predicted protein motifs. Functional studies using heterologous expression systems are necessary to resolve this issue.

**Figure 3:**
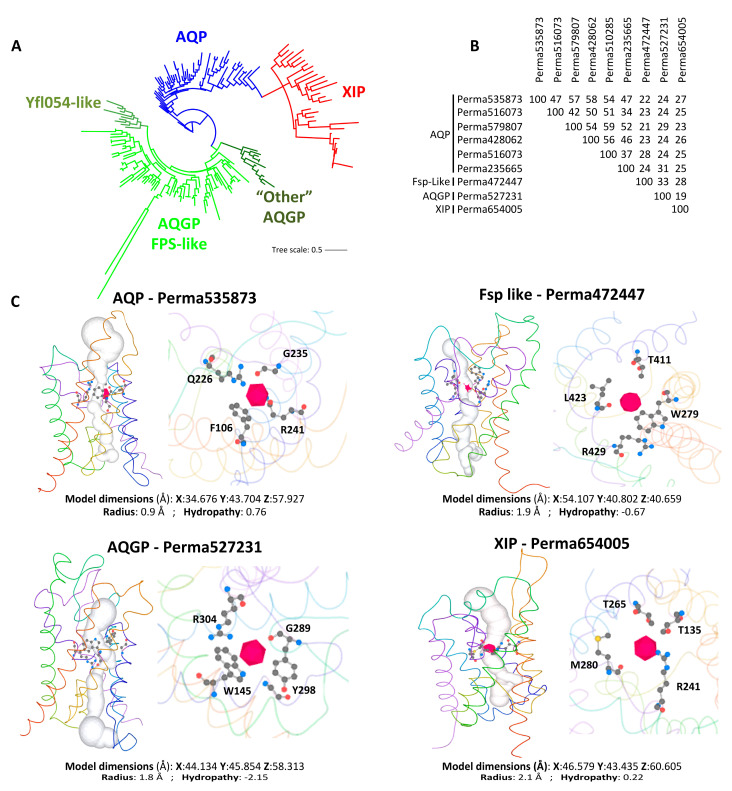
Structure and diversity of fungal aquaporins. (**A**) Maximum-likelihood (1000 bootstrap replicates) phylogenetic tree of 188 full-length aquaporin protein sequences from one species of each major fungal phylum detailed on JGI’s MycoCosm portal. Details of the phylogenetic tree are given in Figure S1. Species and related sequences are listed in Table S2. The nomenclature of each MIP group was established based on the classification adopted for *Trichoderma atroviride* [49] and *Aspergillus niger* [50]. Perma, ***Per**iconia **ma**crospinosa*, XXXXX, protein ID from JGI. (**B**) Protein identity percentage between the nine aquaporins present in the *Periconia macrospinosa* genome, calculated using “Align Sequences Protein BLAST” software on the NCBI resource (https://blast.ncbi.nlm.nih.gov/Blast.cgi; accessed on 1 April 2025). (**C**) Monomeric lateral of the water path (white column) through the “Orthodox” AQP, Fsp-like, AQGP, and XIP aquaporins, including the predicted aromatic-arginine (ar/R) constrictions, which are detailed in the right view. The 3D model, physicochemical properties, and the predicted water column were calculated using Mole 2.5 software. F, Phenylalanine; G, Glutamate; L, Leucine; M, Methionine; Q, Glutamine; R, Arginine; T, Threonine; W, tryptophan; Y, Tyrosine. The number next to each letter corresponds to the position of the amino acid within the primary structure of the protein.

For clades with multiple MIP members, they are classified into three main subfamilies of fungal MIPs: fungal aquaporins (AQPS, which correspond to water channels, including both “orthodox” and “facultative” types), fungal aquaglyceroporins (AQGPs, which facilitate the transmembrane transport of small uncharged molecules and include Fps-like proteins with a conserved regulatory region in the N-terminus, Yfl054p-like proteins with a very long *N*-terminal extension, and “Other” aquaglyceroporins with significant sequences divergence that do not fit into the Fps-like or Yfl054p-like categories), and the unique fungal *X*-intrinsic proteins (XIPs) (Figure 3A and Figure S1). This classification of fungal MIPs is now widely accepted and has been comprehensively detailed in several studies [22,24,25,33,34,35,36].

It is important to emphasize that the number of AQPs and AQGPs can vary significantly between closely related species, plausibly due to overlapping functions as bifunctional water/solute channels. This is exemplified by the AQPs identified in the parasites *Plasmodium falciparum* [37] and *Toxoplasma gondii* [38]. Similarly, in organisms exhibiting more than five aquaporins, the number of each subtype differs: *Trichoderma atroviride* contains three AQPs, three AQGPs, and one XIP [25], while *Periconia macrospinosa* has two AQGPs, six AQPs, and one XIP, with very low sequence identity among subfamilies (Figure 3B). However, it should be stressed that in comparison with the extensive data available on plant and animal AQPs, the knowledge regarding the structural topologies, regulatory mechanisms, permease capacities, and biological functions of fungal AQPs remains limited. Examples of fungal AQPs, such as those from *Saccharomyces cerevisiae* [33,39,40,41], *Laccaria bicolor* [34,42], *Terfezia claveryi* [43,44], *Tuber melanosporum* [45], *Glomus intraradices* [46], *Aspergillus glaucus* [35], *Botrytis cinerea* [47], *Cenococcum geophilum* [48], *Trichoderma atroviride* [25,49], and *Aspergillus niger* [50], illustrate some of the structural and biochemical peculiarities of fungal AQPs, as well as their functional significance in various physiological processes of hyphal cells and their interactions with the biotic and/or abiotic environment.

As we have seen, AQPs are involved in a wide range of processes, acting as key regulators of material and gas exchange, hydro-mineral homeostasis, and cellular signaling. On the other hand, beyond their ecological significance, fungi possess considerable industrial potential across various sectors, including agriculture, pollution control, agri-food, and pharmaceuticals. Then, exploring the diversity of fungal aquaporins represents a highly promising avenue for both fundamental and applied research, opening up new opportunities for the scientific community.

## 3. Case Study of Fungal XIPs—State of the Art

### 3.1. State of the Art

The diversity of fungal aquaporins is relatively well understood; however, the XIP subfamily remains largely unexplored. The second objective of this review is to investigate the origin and diversification of X-intrinsic proteins within the evolutionary history of the MIP superfamily in fungi. XIPs constitute the most recently identified subfamily of AQPs, discovered in the era of high-throughput “omics” technologies. Initially discovered in certain non-vascular plant lineages [18], XIPs have also been characterized in some protozoa [18] and fungal species [34,51]. However, this subfamily remains quite distinct within the MIP family as it is entirely absent from the genome of animals and insects sequenced to date. Similarly, several fungal clades (such as yeasts), Oomycetes, and some plant groups including Coniferophytes [52], Brassicaceae [53], and Monocotyledons [54], also lack the XIP subfamily, which, in plants, has been attributed to functional redundancy [55].

XIPs are localized on the plasma membrane of plant cells [19,56]. Phylogenetic analyses suggest that they are closely related to orthodox AQPs, including those of fungi [49,50]. XIPs exhibit high transport activity for small solutes such as H_2_O_2_ and glycerol, although they show variable activity for water, as demonstrated in heterologous expression experiments using *Xenopus* oocytes or yeast models [19,56]. The biological functions of XIPs have primarily been characterized in plants [19,56,57,58,59,60,61]. Like other MIPs, XIPs are hypothesized to function as significant cellular checkpoints, regulating permeability and osmolarity; however, their biological significance remains largely speculative.

This knowledge gap is even more pronounced in the context of fungi. To date, the most comprehensive structural and functional studies on fungal XIPs come from two studies: one on the *Sordariomycetes* species, *Trichoderma atroviride* [49], a beneficial fungus for sustainable and eco-friendly agriculture, and another on the *Eurotiomycetes* species, *Aspergillus niger*, a major species in the chemical industry (microbial fermentations) and in medicine (as both a food contaminant and a potential human pathogen) [50]. In *Trichoderma atroviride*, the XIP subfamily consists of a single XIP member, which plays a notable role in hyphal development and chlamydosporogenesis. However, despite its involvement in these processes, and plausibly due to its compensating effect with other MIP counterparts, mutants lacking XIP do not show significant changes in mycoparasitic activity against three plant pathogens: *Botrytis cinerea*, *Fusarium graminearum*, and *Rhizoctonia solani*. In *A. niger*, two XIP genes, so-called AQPC and AQPF, are significantly transcribed in response to hydrogen peroxide. However, only AQPF appears to play a role in facilitating the transport this molecule across cellular membranes.

Beyond these studies, several other notable investigations have examined the MIPs of fungal species that possess XIPs in their genomes, such as *Tuber melanosporum* [45], *Cenococcum geophilum* [48], and *Botrytis cinerea* [47]. Paradoxically, none of these studies have specifically addressed the biochemical and/or functional properties of XIPs, with this subfamily consistently overlooked.

### 3.2. Diversity of the Fungal XIPs—Evolution and Topology

The diversity of fungal XIPs has been examined within the context of the fungal kingdom’s exceptionally rich and complex evolutionary history. Given the limited availability of information on XIPs in the current literature, we assembled a dataset of 950 full-length XIP sequences by mining two major public genomic repositories: JGI (http://genome.jgi-psf.org/; 1 April 2025) and NCBI (http://www.ncbi.nlm.nih.gov/; 1 April 2025). This was achieved through a combination of BLAST searches (BLASTp and tBLASTn) and keyword-based queries (i.e., “KOG0223, Aquaporin”, and “PF00230, Major intrinsic protein”), as described in File S1. A comprehensive list of sequences, along with their corresponding species and phylum-level classifications, is provided in Table S1.

**Figure 4:**
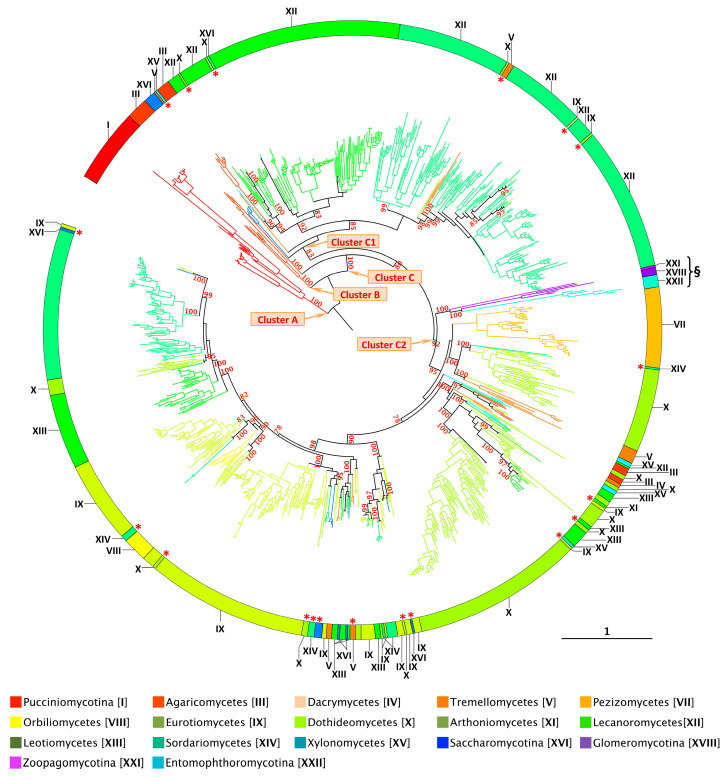
Overview of XIP diversity in fungi. Maximum-likelihood (1000 bootstrap replicates) phylogenetic tree of 950 full-length fungal XIP nucleotide sequences. Only selected significant bootstrap values are displayed; the complete set is provided in Figure S3. The scale bar indicates the number of amino acid substitutions per site. The fungal names are provided in Table S1 and Figure S3. The parameters used for the construction of the phylogenetic tree are available in Files S2 and S3. * Red asterisks indicate sequences with atypical phylogenetic placements that do not cluster within their expected fungal clade but instead group with distantly related fungal groups. **§** Indicates sequences originating from basal fungal phyla: *Glomeromycotina* (XVIII), *Zoopagomycotina* (XXI), and *Entomophthoromycotina* (XXII).

Based on extensive phylogenetic analyses and multiple sequence alignments (methods detailed in File S1), eight principal conclusions were drawn (Table 1 and Figure 2 and Figure 4):

(1) According to currently available sequenced genomes, XIPs appear to be absent from several fungal lineages. Specifically, no XIP sequences were identified in the *Kickxellomycotina* (XXIII), *Mucoromycotina* (XX), *Mortierellomycotina* (XIX), *Taphrinomycotina* (XVII), *Wallmiomycetes* (VI), and *Ustilaginomycotina* (II) (Figure 2). Similarly, XIPs are entirely absent from all lineages within the *Opisthosporidia* subdivision (including *Aphelidiomycota* (XXX), *Cryptomycota* (XXIX), and *Microsporidia* (XXVIII)), as well as from all subdivisions of the *Chytridiomycota* (XXV to XXVII) and the *Blastocladiomycotina* (XXIV). These basal clades, generally regarded as the earliest-diverging lineages of the fungal kingdom, are predominantly composed of unicellular organisms and are characterized by the production of both motile and non-motile sporangiospores [62,63].
(2) In their review of fungal AQPs, Nehls and Dietz [35] reported the presence of a putative XIP sequence in a species belonging to the phylum *Microsporidia*. Although the specific origin of this sequence remains unspecified, its mention is nonetheless significant as it highlights the limitations inherent to bioinformatic approaches commonly used to characterize specific subfamilies. Unless this observation stems from an annotation error or the inadvertent incorporation of foreign genetic material into public databases—an event we have not yet been able to confirm—our analyses suggest that *Microsporidia* do not possess any genuine XIP members (Figure S2 and Table S1). tBLASTn searches and keyword-based queries conducted in both the JGI and NCBI databases consistently retrieve aquaporin sequences that are not related to the XIP subfamily. Furthermore, the protein translations of these candidate sequences exhibit very low sequence identity with known XIPs (approximately 22%), and phylogenetic analyses—including XIP sequences from both closely and distantly related fungal species—do not support the hypothesis that *Microsporidia* harbor members of this aquaporin subfamily. This conclusion appears to extend to all members of the *Chytridiomycetes* and *Opisthosporidia* as well. Continued efforts to identify new species of basal fungi, particularly those inhabiting underexploited environments, combined with expanded genome sequencing initiatives, will be crucial for enriching and potentially refining our understanding of aquaporin diversity and evolution within these early-diverging fungal lineages.
(3) The abundance of XIPs is markedly lower compared with that of the AQP and AQGP subfamilies. In the vast majority of fungal species, the XIP subfamily is represented by a single gene copy, a trend particularly evident in six clades: three within *Agaricomycotina* (II, IV, and V), as well as *Orbiliomycetes* (VIII), *Arthoniomycetes* (XI), and *Saccharomycotina* (XVI). This limited diversity among fungal XIPs is reminiscent of the low number of XIP members observed in plants, as previously reported by Verma et al. [23], and may reflect evolutionary constraints or functional specificity that limit the expansion of this subfamily.
(4) A striking concordance emerges between the phylogenetic distribution of XIPs and the currently established fungal phylogeny, suggesting an evolutionary trajectory in which most XIP members have diversified in a lineage-specific manner across multiple distinct fungal clades (Figure 4 and Figure S3). This distribution pattern parallels the divergence of major fungal phyla, implying that XIPs may have undergone multiple and independent expansion events throughout fungal evolutionary history. Notably, such lineage-specific expansions of XIP genes in fungi closely mirror those documented in plants [19,54], further supporting the hypothesis of convergent evolutionary strategies among eukaryotes adapting to diverse environmental niches.
(5) In contrast to AQPs and AQGPs members, XIPs display an uneven distribution across fungal clades, a pattern that persists even at finer taxonomic resolutions. For example, the class *Sordariomycetes* (XIV) contains the highest number of identified XIPs, totaling 271 sequences. However, these sequences are not uniformly distributed across all subclasses. XIP members have been identified in several orders belonging to four subclasses: *Diaporthomycetidae*, *Sordariomycetidae*, *Xylariomycetidae*, and *Hypocreomycetidae* (Figures S4 and S5). To date, no XIP sequences have been detected within the subclasses *Savoryellomycetidae* and *Lulworthiomycetidae*. This observation highlights the uneven retention of XIPs across fungal lineages and raises questions about the evolutionary and ecological factors underlying the differential presence of this subfamily among closely related taxonomic groups.
(6) Fungal XIPs, based on their nucleotide sequences, are phylogenetically grouped into three well-supported clades (Figure 4 and Figure S3). The first (Cluster A) consists exclusively of members of the Pucciniomycotina (I); the second (Cluster B) comprises a subset of divergent sequences from the Agaricomycetes (III); and the third (Cluster C), which encompasses 96% of the retrieved members, forms the largest group. Notably, this major clade is sub-partitioned into two distinct branches, hereafter referred to as Clusters C1 and C2. This bipartite structure, described here for the first time, is consistently observed in fungal species that harbor multiple XIP paralogs. Despite a relatively low average sequence similarity across the four clusters, approximately 39%, all proteins within both clusters retain conserved molecular signatures characteristic of the XIP family [25,51]. This phylogenetic organization underscores an unexpected level of diversity and divergence within the fungal XIP subfamily. Importantly, the taxonomic distribution across the two clusters is markedly uneven. Cluster C2 contains the most divergent sequences, spanning a broad range of fungal phyla, from basal lineages (XVIII, XXI, and XXII) to more recently evolved groups such as the *Dikarya*. In contrast, Cluster C1 is restricted to the *Dikarya* and includes representatives from only a subset of its constituent phyla. Within the *Dikarya*, certain major phyla are represented exclusively in one cluster: for example, *Pucciniomycotina*, *Pezizomycetes*, and *Lecanoromycetes* in Cluster C2 and *Dacrymycetes*, *Orbiliomycetes*, and *Leotiomycetes* in Cluster C1. Intriguingly, some sequences (highlighted with an asterisk in Figure 4) do not cluster with their expected fungal clades but instead appear among distantly related fungal groups. This applies to several phyla (e.g., *Eurotiomycetes* (IX), *Dothideomycetes* (X), *Sordariomycetes* (XIV), and *Leotiomycetes* (XIII)); however, this pattern is particularly noticeable in *Tremellomycetes* (V), which fails to form a distinct cluster. These atypical phylogenetic placements suggest plausible horizontal gene transfer (HGT) between fungal species, a phenomenon previously reported for other fungal proteins [64,65]. HGT, coupled with the stable integration of the transferred genetic material, may confer adaptive advantages to the host, including enhanced environmental responsiveness and the acquisition of novel traits and functions [66,67]. This hypothesis warrants further dedicated investigation.

Revisiting the example of the *Sordariomycetes* (XIV), the two identified XIP clusters reflect the four major lineage-specific subdivisions within this class: (i) *Diaporthomycetidae* (orders *Diaporthales*, *Togniniales*, Magnaporthales, and *Ophiostomatales*), (ii) *Sordariomycetidae* (orders *Sordariales* and *Chaetosphaeriales*), (iii) *Xylariomycetidae* (order *Xylariales*), and (iv) *Hypocreomycetidae* (orders *Glomerellales*, *Microascales*, and *Hypocreales*) (Figure S4). While Cluster C2 includes representatives from all these taxonomic groups, Cluster C1 is restricted to sequences from *Diaporthomycetidae* and *Hypocreomycetidae*. Notably, two sequences from *Apiospora montagnei* (order: *Xylariales*) fall within Cluster C2 and display a highly degree of similarity (62% identity), likely reflecting a recent gene duplication event. In contrast, *Trichoderma virens* (order *Hypocreales*) possesses two highly divergent XIP homologs (33% identity) that segregate into both Clusters C1 and C2, suggesting the presence of two ancestral “XIP archetypes” prior to the diversification of the *Sordariomycetes* lineage. This pattern appears to be broadly representative of the major fungal phyla distributed across the two clusters.

Collectively, these findings support the hypothesis that the four XIP clusters originated from ancient out-paralogs that arose in the common ancestor of the *Dikarya*, followed by lineage-specific losses of one cluster over evolutionary time. To the best of our knowledge, this is the first report of such a dichotomous organization among fungal XIPs, which, intriguingly, closely mirrors the phylogenetic structuring reported for plant XIPs within the Viridiplantae [19,54]. This similarity reinforces the notion of a conserved evolutionary trajectory across two distantly related eukaryotic lineages: fungi and plants.

(7) The amino acid length of fungal XIPs ranges from 274 to 362 residues, with an average of 326, corresponding to theoretical molecular weights between 29.17 and 38.82 kDa (mean: 34.97) (Table 1). The isoelectric points (pI) of these proteins span a range from 6.41 to 8.22, suggesting functional constraints potentially linked to regulatory mechanisms. These include conserved regulatory motifs in the *C*-terminal regions and in several electrostatic loops, which may be involved in post-translational modifications such as methylation, phosphorylation, and trafficking, as well as interactions with regulatory partners. Such modifications, known to vary among MIP families and subfamilies, underscore the multifunctional and dynamic regulation of solute permeability mediated by AQPs; this phenomenon is documented in plants and animals [68,69] but still insufficiently characterized in filamentous fungi. Additionally, XIPs exhibit favorable biochemical properties, including high intrinsic stability (instability index < 40), and thermal resilience, as indicated by an average aliphatic index of 95. These features are characteristic of canonical MIP protomers. In silico predictions of subcellular localization predominantly place XIPs in the plasma membranes; however, a marginal presence in other cellular compartments—such as the mitochondria, endoplasmic reticulum, and peroxisomes—cannot be excluded. In this context, the positive GRAVY (grand average of hydropathy) index of 0.389 further supports their hydrophobic nature, a fundamental trait for aquaporins that facilitates efficient transmembrane transport of solutes [70].
(8) As expected for members of the MIP superfamily, the protomeric architecture of all fungal XIPs is highly conserved and adopts the canonical hourglass configuration. This structure comprises six transmembrane helices (TMH1 to TMH6), connected by five inter-helical loops (Loop A to E), with both the *N*- and *C*-terminal regions oriented toward the cytosolic side (Figure 3C and Table 1). Centrally located within the pore are two half-helices, derived from loops B and E, each capped by conserved “NPA” motifs, along with the ar/R selectivity filter. The latter is composed of specific hydrophobic amino acids whose side chains line the pore and form a narrow constriction at the extracellular entrance. Although this ar/R filter appears to be somewhat less hydrophobic than its counterparts in dicotyledonous plants and moss [51], it is likely optimized for the transmembrane passage of relatively large and/or hydrophobic solutes, such as glycerol, urea, ammonia (NH_3_), lactic and boric acids, polyols, and various metalloids or ions including arsenic (As), antimony (Sb), zinc (Zn^2+^), aluminum (Al^3+^), silicon (Si), and selenium (Se). Notably, there is considerable variability in the amino acid residues lining the channel both between and within fungal phyla, which may reflect adaptations to distinct physiological environments and suggest divergent substrate specificities of XIPs across fungal lineages.

### 3.3. Fungal XIPs: A Relatively Recent Chapter in the Evolution of Fungal AQP

The most ancient fungal phyla in which genomes contain at least one XIP-encoding gene belong to *Zoopagomycota*, specifically within the subphyla *Zoopagomycotina* and *Entomophthoromycotina*. *Zoopagomycota* represent the most basal lineage among zygomycetous fungi and are notably associated with three major phenotypic transitions in the fungal evolutionary history: successful adaptation to terrestrial environments (terrestrialization), the emergence of zygospores and novel sexual reproductive structures, and the acquisition of the spindle pole body (SPB) [63]. These findings support the hypothesis that fungal XIPs emerged more recently than the two other major aquaporin subgroups—AQPs and AQGPs—as previously suggested by [24]. This emergence is estimated to have occurred approximately 450 million years ago (Mya), potentially coinciding with or shortly following the divergence of the *Mucoromycota* and *Dikarya* subkingdoms, which occurred between 350 and 400 Mya. It is therefore plausible to propose that fungal XIPs originated from a common ancestral gene that duplicated early in the evolutionary history of the fungal kingdom, followed by a significant diversification burst, particularly within the *Dikarya*. This evolutionary window overlaps with the Silurian and Devonian geological periods, a critical epoch in the history of eukaryotic biodiversity. This period is marked by increased terrestrialization events, the emergence and complexification of multicellular body plans (exemplified in fungi by the development of non-flagellated, multicellular thalli composed of apically growing and branching hyphae, whose development and spatial organization exhibit characteristic analogous to fractal geometry), the expansion of terrestrial biodiversity, and the plateauing of atmospheric O_2_ concentrations at approximately 21% [71,72,73]. Interestingly, the timing of complex innovation in the fungal multicellularity coincides with the explosive diversification of terrestrial animals and plants, suggesting a possible co-evolutionary relationship between fungi and *Embryophyta* (land plants), which also experienced a major radiation around 430–450 Mya [74,75].

Within this evolutionary and ecological context, the expansion of the XIP family appears to have paralleled the early colonization of terrestrial habitats by emerging eukaryotic biodiversity. Nonetheless, despite these broadly similar evolutionary trajectories, the expansion and diversification of the XIP subfamily in fungi appears to have been more constrained than in plants. While land plant genomes frequently encode multiple XIP isoforms [19,54], fungal genomes typically harbor only a single XIP candidate per species. This pattern suggests that although the XIP archetype has been maintained across both fungal and plant lineages, its evolutionary expansion has followed markedly divergent trajectories. These differences likely reflect distinct functional roles and regulatory constraints within the respective cellular architectures and ecological niches of fungi and plants.

### 3.4. Fungal XIP—Outstanding Questions

Despite the high sequence diversity among XIP homologs, accumulating evidence supports the hypothesis that XIPs have a deep evolutionary origin [76]. This is substantiated by the presence of key conserved residues and a set of synapomorphic amino acids identified in XIPs across a broad spectrum of eukaryotic lineages, including Angiosperms, Bryophytes, *Dyctyostelium* (Amoebozoa), Chlorarachniophyte algae (*Rhizaria*), a Stramenopile (diatom), and fungi [22,51]. Although absent in many Protists, Oomycetes, and Metazoans, available evidence suggests that an ancestral XIP-like protein was already present in certain early eukaryotic ancestors. This ancestral archetype likely underwent extensive diversification, potentially driven by an ancient burst of gene duplication, which, according to the most recent estimates of the eukaryotic molecular clock, may have occurred over 2 billion years ago [77,78].

Within the fungal kingdom, the phylogenetic topology of the XIP subgroup is paraphyletic, encompassing members with varying degrees of relatedness. This paraphyly implies the emergence of a structural and functional diversification of fungal XIPs, potentially shaped by distinct ecological and physiological pressures, such as nutritional strategies, habitat specificity, or biotic interactions. The retention of XIP genes in some fungal lineages, alongside their absence in others, highlights unresolved questions concerning the selective forces that may underpin their maintenance or loss. Elucidating the roles of XIPs in fungal growth, development, and solute homeostasis could provide key insights into their evolutionary significance. Notably, only a limited number of fungal species within each phylum possess identifiable XIP orthologs.

Given the current state of limited knowledge, no clear functional rationale has yet emerged to explain the selective presence of the XIP family in certain fungal species. This uncertainty gives rise to several key questions:

- Do the expansion and relative conservation of the XIP subgroup within the *Dikarya*, together with its apparent correlation to the terrestrialization of the fungal kingdom, point to a specialized function for these proteins in transporting solutes essential to the full developmental cycle of these fungi?
- Could the concomitant presence of XIP homologs in both fungal and green plant lineages be associated with their parallel evolutionary success of in terrestrial ecosystems? This prompts broader questions regarding the potential roles of XIPs as an evolutionary link between these two kingdoms, whose ecological interdependence is both diverse and deep. Despite their distinct phylogenetic classifications, remarkably, XIP proteins in plants and fungi share striking structural similarities, hinting at a plausible shared ancestral origin.
- Do the two divergent XIP archetypes observed across fungal species reflect a degree of functional specialization, potentially involving distinct solute transport profiles and regulatory mechanisms, or are they associated with tissue-specific expression patterns in multicellular organisms, indicative of sub-functionalization?

Expanding the scope of phylogenetic analyses to include a broader range of XIP sequences should improve the resolution of these outstanding questions. More broadly, unlike MIP families in animals and plants, fungal MIP repertories are typically composed of a relatively small number of isoforms. This trend is particularly pronounced for the XIP group, which is generally represented by a single isoform in the majority of fungal species. Additionally, the spectrum of substrates transported by fungal MIPs—especially XIPs—remains largely uncharacterized. This raises the possibility that the limited number of fungal MIP channels may have evolved multifunctional capabilities, thereby compensating for reduced isoform diversity through functional versatility adapted to the wide array of ecological niches that fungi inhabit.

Collectively, these observations offer novel perspectives on the classification and evolutionary history of fungal XIPs and lay the groundwork for future investigations into their functional and ecological relevance.

## 4. Conclusions

The extensive body of research on aquaporins (AQPs) has yielded highly detailed insights into their diversity, topology, function, regulatory mechanisms, transported solutes, and evolutionary trajectories across biological kingdoms. While AQPs in plants and animals are the most thoroughly studied, largely due to their economic and biomedical relevance, substantial knowledge gaps persist regarding fungal AQPs, particularly those belonging to less common subfamilies such as XIPs. A key current challenge is to achieve a comprehensive understanding of how fungal AQPs, AQGPs, and XIPs contribute to fundamental biological processes, including growth, development, defense mechanisms, and overall fitness under fluctuating environmental conditions. This entails elucidating their roles in diverse fungal lifestyle strategies and their involvement in the establishment and evolution of specialized interactions with surrounding organisms, including animals, insects, and plants.

The number of functionally characterized fungal aquaporins remains limited, making it difficult to draw generalizable conclusions. However, in light of the animal models and the evolutionary complexity of fungal cell biology, it is reasonable to anticipate that in fungal genomes encoding multiple potentially expressible AQP isoforms, these proteins exhibit specialized expression patterns and subcellular localization, depending on developmental stage and specific cellular structures. Physiologically, AQPs allow for the rapid modulation of water permeability in response to osmotic stress, particularly during the influx and efflux of solutes between the cytosol and the extracellular environment. Likewise, AQPs facilitate the uptake of key metabolic precursors and nutrients (e.g., glycerol, nitrogen, gases, etc.), as well as the expulsion of potentially toxic metabolic byproducts, such as methylglyoxal or ammonia. In addition, from a therapeutic perspective, fungal AQPs have already emerged as promising drug targets and potential gateways for antifungal compounds [79]. The development of high-affinity and highly specific inhibitors targeting these channels remains a critical avenue for future research, with potential applications in the treatment of severe cryptogamic diseases affecting both plants and animals.

In brief, a major research priority will be to predict and characterize the diverse ecological functions of MIPs across the fungal kingdom, guided by large-scale structural and phylogenetic analyses. Integrating multidisciplinary approaches, from molecular biology and structural bioinformatics to ecology and biotechnology, will be essential for advancing our understanding of the physiological roles of AQP subclasses and their contributions to fungal biology.

## Figures and Tables

**Table 1:** Protein characteristics of the fungal XIP sequences identified through the JGI and NCBI databases.

(A) XIP Physicochemical Properties ***										
Code *	Phyla	Cluster **	Size (AA)	MW (kDa)	*p*I Moyen		Instability Index		Aliphatic Index	GRAVI
I	*Pucciniomycotina*	A	275 ± 7	29.174 ± 1.292	7.36 ± 0.71		30.09 ± 2.24		99.81 ± 6.62	0.585 ± 0.071
III	*Agaricomycetes*	B-C1-C2	334 ± 21	35.411 ± 1.962	7.54 ± 1.18		33.87 ± 6.83		95.46 ± 5.34	0.386 ± 0.093
IV	*Dacrymycetes*	C2	322 ± 1	35.793 ± 0.334	8.22 ± 0.64		35.49 ± 1.85		95.78 ± 6.41	0.376 ± 0.057
V	*Tremellomycetes*	C1-C2	326 ± 8	38.824 ± 1.018	8.22 ± 1.13		37.79 ± 6.68		95.12 ± 6.59	0.336 ± 0.105
VII	*Pezizomycetes*	C2	316 ± 9	33.123 ± 1.256	7.71 ± 1.47		34.98 ± 4.79		98.73 ± 8.07	0.399 ± 0.111
VIII	*Orbiliomycetes*	C2	351 ± 9	37.624 ± 0.964	8.18 ± 0.88		35.99 ± 2.96		89.01 ± 2.31	0.282 ± 0.061
IX	*Eurotiomycetes*	C1-C2	326 ± 13	35.050 ± 1.344	7.53 ± 1.01		36.49 ± 4.59		96.09 ± 3.21	0.429 ± 0.087
X	*Dothideomycetes*	C1-C2	323 ± 7	34.369 ± 0.914	7.29 ± 0.93		36.21 ± 4.94		99.11 ± 4.67	0.391 ± 0.086
XI	*Arthoniomycetes*	C2	323	34.2391	6.41		40.04		93.62	0.355
XII	*Lecanoromycetes*	C1	313 ± 14	33.297 ± 1.747	7.04 ± 1.15		33.42 ± 4.59		91.07 ± 3.82	0.361 ± 0.097
XIII	*Leotiomycetes*	C2	319 ± 13	34.358 ± 1.569	7.92 ± 0.71		36.63 ± 5.13		93.28 ± 4.51	0.354 ± 0.075
XIV	*Sordariomycetes*	C1-C2	332 ± 12	35.012 ± 1.141	7.58 ± 1.03		36.46 ± 4.41		89.55 ± 4.47	0.388 ± 0.097
XV	*Xylonomycetes*	C1-C2	324 ± 2	34.687 ± 0.311	7.062 ± 1.01		34.46 ± 3.33		96.03 ± 2.57	0.374 ± 0.033
XVI	*Saccharomycotina*	C1-C2	316 ± 5	33.998 ± 0.762	7.78 ± 0.28		34.12 ± 3.12		94.66 ± 4.99	0.373 ± 0.067
XVIII	*Glomeromycotina*	C2	346 ± 22	38.362 ± 2.395	6.55 ± 0.73		39.80 ± 2.98		102.52 ± 8.08	0.297 ± 0.157
XXI	*Zoopagomycotina*	C2	317	34.521	6.69		25.85		103.47	0.29
XXII	*Entomophthoromycotina*	C2	334 ± 10	36.010 ± 0.981	6.36 ± 0.51		34.91 ± 4.66		93.83 ± 4.21	0.337 ± 0.072
		**Mean**	326	34.973	7.24		35.34		95.46	0.356
		**SD**	12	1.537	0.60		3.46		4.53	0.044
(B) XIP Structure Singularities ****										
Code *	**Phyla**	**TMH**	**SubCL**	**NPA-LB**		**NPA-LE**		**Ar/r**		
I	*Pucciniomycotina*	6	PM	(**N**S)**P**(**IF**LV)		(**F**Y)**P**(**NA**T)		(**VY)**(**VN**CA)(**V**I)**R**		
III	*Agaricomycetes*	6	PM	**N**(**P**S)(**LM**)		(**FN**)**P**(**NA**GS)		(**N**TS)(**VG**TN)(**SL**GC)**R**		
IV	*Dacrymycetes*	6	PM	**NP**(**MT**)		**NPA**		**N**(**ST**)**SR**		
V	*Tremellomycetes*	6	PM(Mito)	**NP**(**LI**MF)		**NP**(**AT**GS)		(**N**T)(**TV**FM)(**Q**S)**R**		
VII	*Pezizomycetes*	6	PM(Pero)	**N**(**P**S)(*L*M)		**NP**(**AS**TCG)		(**I**AV)(**ST**E)**SR**		
VIII	*Orbiliomycetes*	6	PM	**NPL**		**NP**(**A**GT)		(**S**A)(AI)(**A**S)**R**		
IX	*Eurotiomycetes*	6	PM	(**N**PS)**P**(**TL**M)		**NPA**		**(NAQ)**(**SA**T)(**S**CGA)**R**		
X	*Dothideomycetes*	6	PM	**NP**(**L**FTSA)		**N**(**P**S)A		(**N**TQ)(**S**TL)(**S**GA)**R**		
XI	*Arthoniomycetes*	6	PM	**NPM**		**NPA**		**QICR**		
XII	*Lecanoromycetes*	6	PM	**NP**(**MT**L)		(**N**H)**P**(**A**T)		(**N**S)(**S**TG)(**SG**A)**R**		
XIII	*Leotiomycetes*	6	PM	**NP**(**VTI**LM)		**NP**(**AS**G)		(**N**S)(**A**STL)(**G**N**S**)**R**		
XIV	*Sordariomycetes*	6	PM	**NP**(**LV**MTSA)		(**N**MH)**P**(**AS**TG)		(**N**CST)(**VS**TAV)(**QS**GN)R		
XV	*Xylonomycetes*	6	PM	**N(PSQ**)(**TFL)**		**(NF)P(ASG)**		(**LT**N)(**AG**T)**SR**		
XVI	*Saccharomycotina*	6	PM	**NP(LTI)**		**NP(GA)**		(**NG**I)(**IS**)**SR**		
XVIII	*Glomeromycotina*	6	PM(Mito)	**N**(**P**S)(**FL**)		**NP(AV)**		**H**(**V**A)**SK**		
XXI	*Zoopagomycotina*	6	PM	**NPS**		**NPA**		**H**I**SR**		
XXII	*Entomophthoromycotina*	6	PM	**N**(**P**T)(**ML**CI)		**NP**(**AG)**		(**VT**)**IGK**		

(**A**) XIP physicochemical properties. (**B**) XIP structure singularities. * Code related to the phylogenetic distribution of the major fungal phyla. ** Clusters were determined based on the phylogenetic distribution of the 950 XIP sequences. *** Physicochemical properties were predicted using the ProtParam tool (Expasy portal): AA, number of amino acids; MW, molecular weight; *p*I, isoelectric point; GRAVI, grand average of hydropathicity. **** Structural features were predicted through sequence alignment. Transmembrane helices (TMHs) were predicted using TMHMM, and subcellular localization (SubCL) was assessed using WoLF PSORT.

## Data Availability

The original contributions presented in this study are included in the article/Supplementary Materials. Further inquiries can be directed to the corresponding author.

## Supplementary Materials

The following supporting information can be downloaded at https://www.mdpi.com/article/10.3390/jof11070543/s1: File S1: The materials and methods used to generate the new data that supplement this review. File S2: MAFFT_TrimAL_IQ-tree1000fstBtp_ML_Figure3.nhx. File S3: Tol_color_strip_Figure3. File S4: MIPs from the Chytridiomycota and Opisthosporidia phyla (MycoCosm portal of JGI), along with XIP sequences from one representative fungal strain per phylum where XIP sequences are present. The assignment of the AQP and AQGP subgroups is putatively predicted based on an alignment that includes the characterized orthodox AQP, AQGP, Yfl054-like, Fps-like, “Other” AQGP, and XIP sequences of Trichoderma atroviride (highlighted by an asterisk) [25]. Figure S1: Phylogenetic analysis of full-length aquaporin nucleotide sequences from one species of each major fungal phylum detailed on JGI’s MycoCosm portal. The assignment of the AQP, AQGP, Yfl054-like, Fps-like, “Other” AQGP, and XIP subgroups is predicted based on the characterized heterologous AQP sequences of *Trichoderma atroviride* [25] and *Aspergillus niger* [50] (highlighted by one or two asterisks, resp.). Species and related sequences are listed in Table S2. Tree Inference was performed on the maximum likelihood (1000 bootstrap replicates), using FastTree program and based on a MAFFT alignment, and edited with ITOL V7. Figure S2: Phylogenetic distribution of all MIPs extracted from the *Chytridiomycota* and *Opisthosporidia* phyla (MycoCosm portal of JGI), along with XIP sequences from one representative fungal strain per phylum where XIP sequences are present. These XIPs are those used in the phylogeny presented in Figure 3A. All the MIP sequences of the basal phyla are available in File S4, and the XIP sequences in Table S1. The assignment of the AQP and AQGP subgroups is putatively predicted based on an alignment that includes the characterized orthodox AQP, AQGP, Yfl054-like, Fps-like, “Other” AQGP, and XIP sequences of *Trichoderma atroviride* (highlighted by an asterisk) [25]. Sequences for which it is uncertain to assign a subgroup without further biochemical and bioinformatic analysis are named MIP and colored in purple. Tree Inference was performed on the maximum likelihood (1000 bootstrap replicates). Figure S3: Detailed view of the maximum likelihood (1000 bootstrap replicates) tree based on 950 full-length fungal XIP nucleotide sequences, presented in Figure 4. The scale bar represents the number of amino acid substitutions per site. The parameters used for the construction of the phylogenetic tree are available in Files S2 and S3. The complete fungal names corresponding to the sequences are provided in Table S1. Figure S4: Maximum-likelihood phylogenetic tree of 271 nucleotide sequences from *Sordariomycetes*. The bootstrap values indicated at the nodes are based on 1000 bootstrap replicates. Branch values lower than 50% are hidden. The tree scale denotes the evolutionary distance expressed at the number of nucleotide substitutions per site. Clusters C1 and C2 correspond to the primary clusters illustrated in Figure 4. Accession numbers and sequences of *XIP* are provided in Table S1. Figure S5: Modified representation of the *Sordariomycetes* maximum likelihood phylogenetic tree from 345 taxa (1000 bootstraps), based on Hongsanan et al. [80]. Orders containing fungal species with identified XIP sequences in their genomes are highlighted in red. Branch lengths are not proportional to genetic distances. Table S1: Set of XIP sequences. Table S2: Full-length aquaporin protein sequences from one species of each major fungal phylum detailed on JGI’s MycoCosm portal. References [81,82,83,84,85,86,87,88,89,90] are cited in the Supplementary Materials.
