# Supplementary material for: Major Intrinsic Proteins in Fungi: A Special Emphasis on the XIP Subfamily
Source: J Fungi (Basel). 2025 Jul 21;11(7):543. doi: 10.3390/jof11070543 (PMC12300952; doi:10.3390/jof11070543)
Supplement: Supplementary file 1 [file jof-11-00543-s001.zip › jof-3752183_Supplementary_Figure_S1.pdf]

Supplementary Figure S1

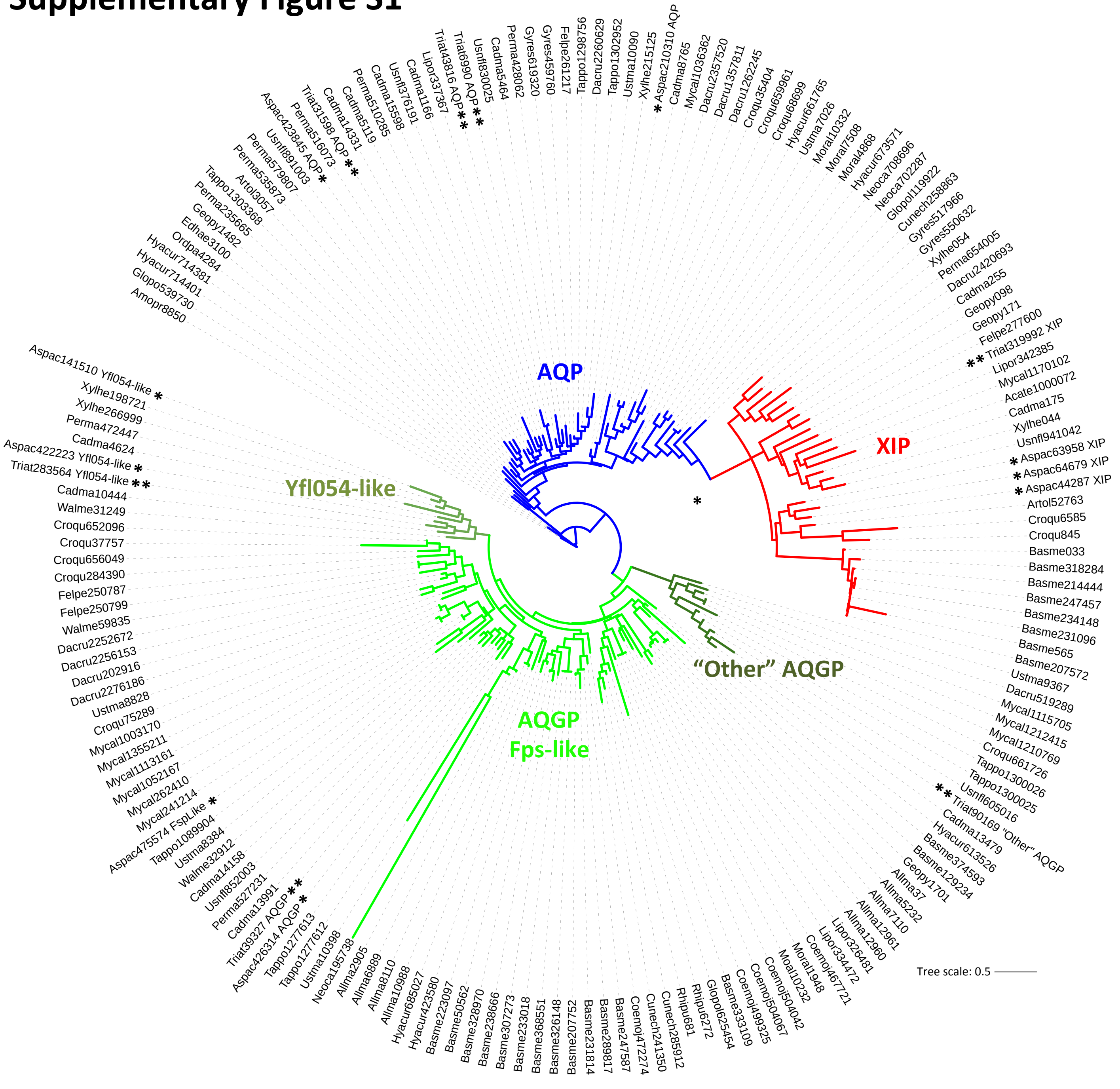

Supplementary Figure S1. Phylogenetic analysis of full-length aquaporin protein sequences from one species of each major fungal phylum detailed on JGI's MycoCosm portal. The assignment of the AQP, AQGP, Yfl054-like, Fps-like, "Other" AQGP, and XIP subgroups is predicted based on the characterized heterologous AQP sequences of *Trichoderma atroviride* [**25**] and *Aspergillus niger* [**50**] (highlighted by one or two asterisks, resp.). Species, and related sequences are listed in Supplementary Table S2. Tree Inference was done on the maximum-likelihood (1,000 bootstrap replicates).
