# Supplementary material for: Major Intrinsic Proteins in Fungi: A Special Emphasis on the XIP Subfamily
Source: J Fungi (Basel). 2025 Jul 21;11(7):543. doi: 10.3390/jof11070543 (PMC12300952; doi:10.3390/jof11070543)
Supplement: Supplementary file 1 [file jof-11-00543-s001.zip › jof-3752183_Supplementary_Figure_S2.pdf]

Supplementary Figure S2

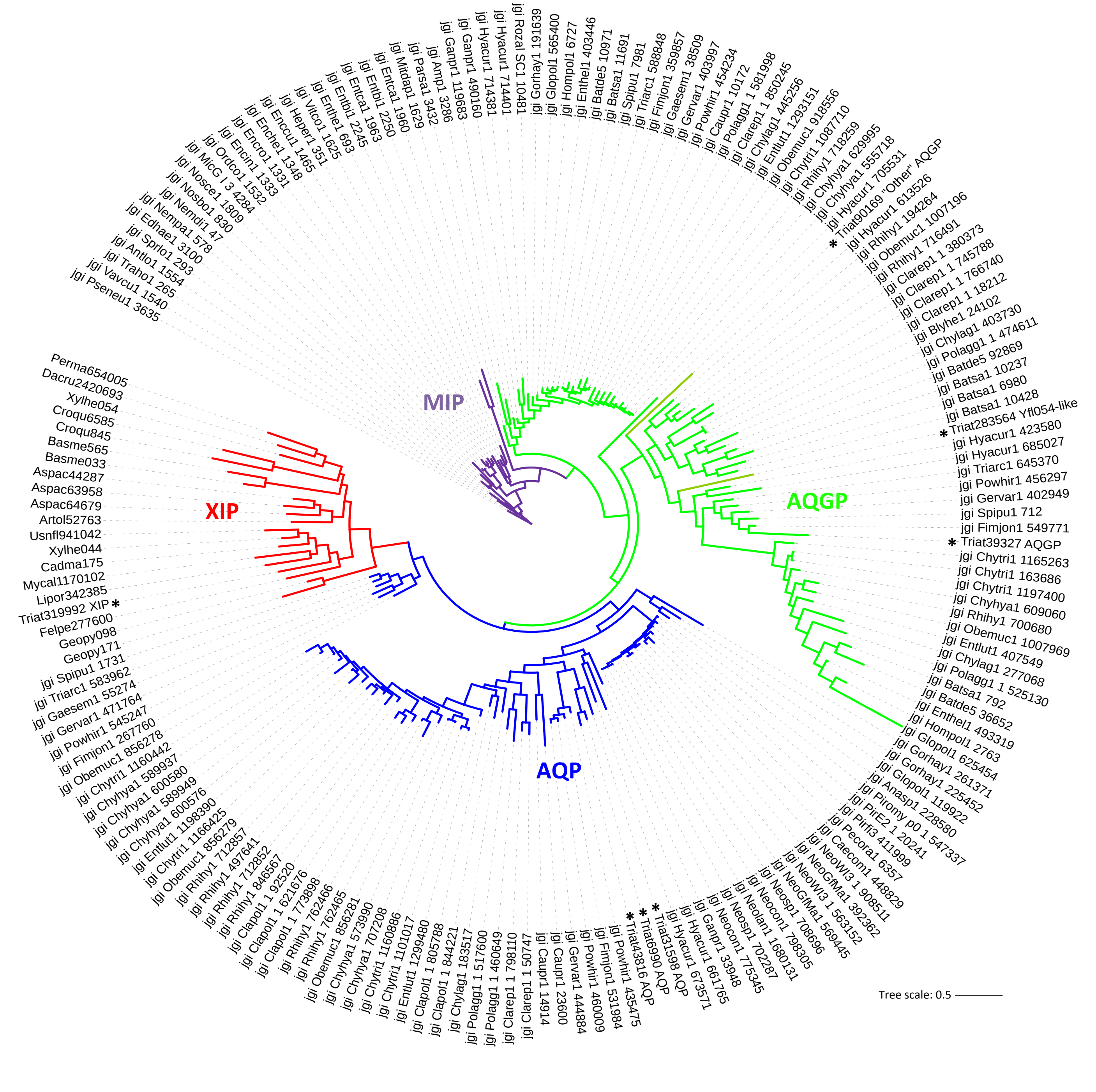

Supplementary Figure S2. Phylogenetic distribution of all MIPs extracted from the *Chytridiomycota* and *Opisthosporidia* phyla (MycoCosm portal of JGI), with one XIP member from each phylum that possesses one. These XIP are those used in the phylogeny presented in Figure 3a. All the MIP sequences of the basal phyla are available in Supplementary File S2, and the XIP sequences in Supplementary Table S1. The assignment of the AQP and AQGP subgroups is putatively predicted based on an alignment that includes the characterized orthodox AQP, AQGP, Yfl054-like, Fps-like, “Other” AQGP, and XIP sequences of *Trichoderma atroviride* (highlighted by an asterisk) [**25**]. Sequences for which it is uncertain to assign a subgroup without further biochemical and bioinformatic analysis are named MIP and colored in purple. Tree Inference was done on the maximum-likelihood (1,000 bootstrap replicates).
