# Supplementary material for: Major Intrinsic Proteins in Fungi: A Special Emphasis on the XIP Subfamily
Source: J Fungi (Basel). 2025 Jul 21;11(7):543. doi: 10.3390/jof11070543 (PMC12300952; doi:10.3390/jof11070543)
Supplement: Supplementary file 1 [file jof-11-00543-s001.zip › jof-3752183_Supplementary_Figure_S4.pdf]

Supplementary Figure S4

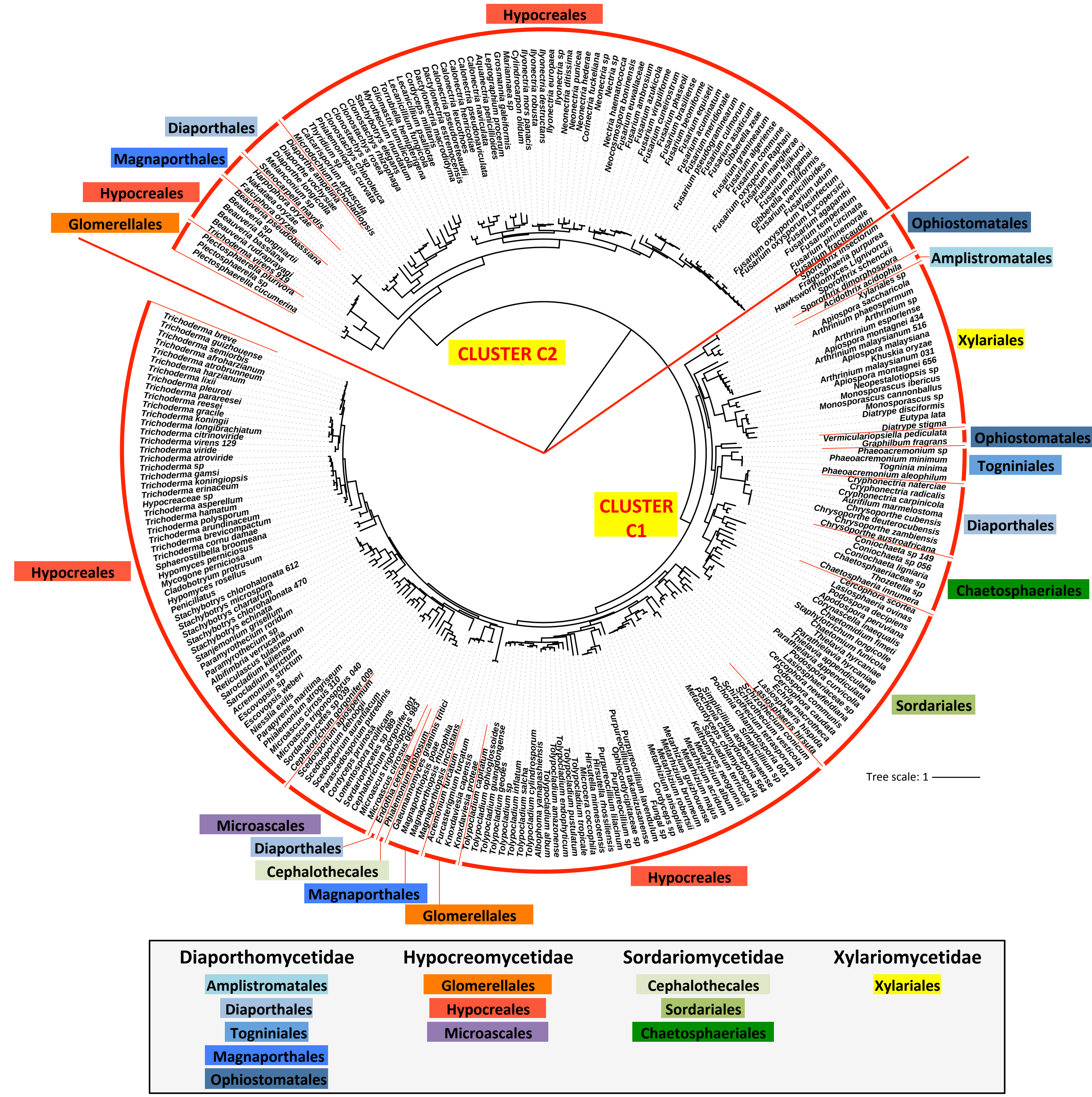

Supplementary Figure S4. Maximum-likelihood phylogenetic tree of 271 nucleotide sequences from *Sordariomycetes*. The bootstrap values indicated at the nodes are based on 1,000 bootstrap replicates. Branch values lower than 50% are hidden. The tree scale denotes the evolutionary distance expressed at the number of nucleotide substitutions per site. Clusters C1 and C2 correspond to the primary clusters illustrated in Figure 4. Accession numbers and sequences of *XIP* are provided in Supplementary Table S1.
