# Supplementary material for: Major Intrinsic Proteins in Fungi: A Special Emphasis on the XIP Subfamily
Source: J Fungi (Basel). 2025 Jul 21;11(7):543. doi: 10.3390/jof11070543 (PMC12300952; doi:10.3390/jof11070543)
Supplement: Supplementary file 1 [file jof-11-00543-s001.zip › jof-3752183_Supplementary_Figure_S5.pdf]

Supplementary Figure S5

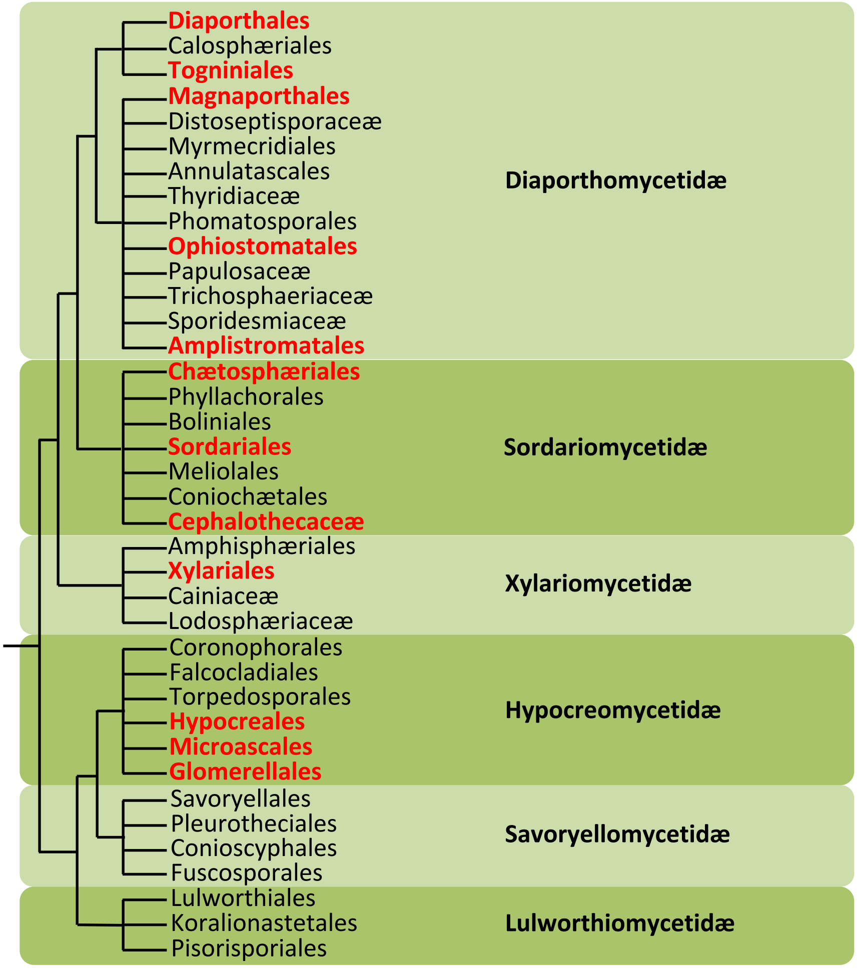

Supplementary Figure S5. Modified representation of *Sordariomycetes* Maximum Likelihood phylogenetic tree from 345 taxa (100 bootstraps), based on Hongsanan *et al* [80]. Orders containing fungal species with identified XIP sequences in their genomes are highlighted in red. Branch lengths are not proportional to genetic distances.
