# Supplementary material for: Major Intrinsic Proteins in Fungi: A Special Emphasis on the XIP Subfamily
Source: J Fungi (Basel). 2025 Jul 21;11(7):543. doi: 10.3390/jof11070543 (PMC12300952; doi:10.3390/jof11070543)
Supplement: Supplementary file 1 [file jof-11-00543-s001.zip › jof-3752183_Supplementary_File S1_Material_&_methods.pdf]

Supplementary File S1 - Material and methods

Major Intrinsic Proteins in Fungi.
A Special Focus on the XIP Subfamily

VENISSE Jean-Stéphane, BRONNER Gisèle, SAADAOUI Mouadh, Patricia Roeckel-Drevet, FAIZE Mohamed, FUMANAL Boris

Supplementary File S1 - The materials and methods used to generate the new data that supplement this review

Fungal XIP retrieval

The fungal XIP gene subfamily was investigated using previously identified XIP sequences from selected fungal species [**25**,**50**,**51**] as initial queries. Searches were conducted using BLAST tools: tBLASTn and BLASTp [**81**], with stringent parameters, applying an *E*-value cutoff of 1.0×10^{-5}. These searches targeted several standard databases from the National Center for Biotechnology Information (NCBI; *http://www.ncbi.nlm.nih.gov/*), including the Core Nucleotide (core_nt), Nucleotide Collection (nr/nt), Whole-Genome Shotgun Contigs (wgs), Expressed Sequence Tags (est), Transcriptome Shotgun Assembly (TSA), and Genomic Survey Sequences (gss), as well as MicoCosm resources from the Department of Energy's Joint Genome Institute (JGI; *http://www.jgi.doe.gov/*) [**32**].

Each putative retrieved MIP sequence was thoroughly examined for the presence of characteristic AQP motifs -including the NPA motifs, the ar/R selectivity filter, and Froger's positions (FPs)- as well as predicted transmembrane topology. Analyses were conducted using InterProScan (EMBL-EBI; *http://www.ebi.ac.uk/Tools/pfa/iprscan/*; accessed on 5 May 2025) and the NCBI Conserved Domain Database (https://www.ncbi.nlm.nih.gov/Structure/cdd/wrpsb.cgi; accessed on 2 July 2021). Percentages of amino acid identity were calculated using "Align Sequences Protein BLAST" tool on the NCBI resource.

Each putative retrieved MIP sequence was validated by predicting the presence of the two canonical "NPA" motifs, and the transmembrane topologies using InterProScan (EMBL-EBI;

http://www.ebi.ac.uk/Tools/pfa/iprscan/; accessed on 5 May 2025) and the NCBI Conserved Domain Database (*https://www.ncbi.nlm.nih.gov/Structure/cdd/wrpsb.cgi*; accessed on 5 May 2025). Concomitantly, motifs were identified by multiple sequence alignment and performed using MUSCLE (*https://www.ebi.ac.uk/Tools/msa/muscle/*; accessed on 5 May 2025) [**82**]. Incomplete sequences showing conserved regions were manually assembled for downstream analysis. Sequences that could not be reconstructed to their full-predicted length were excluded from the analysis.

All newly identified XIP candidates were specifically annotated for this study using a standardized gene nomenclature format: genus (*three-letter code*) - species (*two-letters code*) - gene name (locus), or alternatively, MIP nomenclature (subfamily x;y). In cases where multiple XIP sequences were identified within a single species, each member was distinguished using the last three digits of its corresponding protein or gene accession number.

Characterization of biochemical features

Amino acid (AA) sequences were obtained *via* translation using Expasy Bioinformatics Resource Portal (*https://web.expasy.org/translate/*). Key protein features, including theoretical isoelectric point (*p*I), molecular weight (MW), the grand average of hydropathicity (GRAVY), and instability index, aliphatic index were computed using the ProtParam tool (*https://web.expasy.org/protparam/*). Conserved domains such as NPA motifs and the ar/R filter were manually annotated based on multiple sequence alignments with heterologous XIP described in previous work [**25**,**50**,**51**].

Transmembrane regions were predicted using TMHMM (*www.cbs.dtu.dk/services/TMHMM/*) [**83**] and SOSUI (*http://harrier.nagahama-i-bio.ac.jp/sosui/sosui_submit.html*) [**84**], and were manually refined when necessary using comparative data from heterologous fungal XIP sequences [**25**,**50**,**51**]. Subcellular localization was predicted using WoLF PSORT (*http://wolf-psort.hgc.jp*).

Homology-based tertiary structure prediction

Homology-based prediction of protein tertiary structures were performed using the Phyre 2 protein modeling server (*http://www.sbg.bio.ic.ac.uk/phyre2/html/page.cgi?id=index*; accessed on 5 May 2025) [**85**], employing the intensive modeling mode. The resulting structures, provided in PDB format, were subsequently analyzed using the Mole 2.5 server (*https://mole.upol.cz/*; accessed on 5 May 2025) [**86**] to predict transmembrane pores and assess various biochemical properties of the pore-lining residues. Default settings were applied, with heteroatoms excluded for the modeling process and pore merging enabled. Channel modeling parameters were as follow: cavity probe radius, 5 Å; cavity interior threshold, 1.1 Å;

channel origin radius, 5 Å; channel surface cover radius, 5 Å; channel weight function, Voronoi scale; bottleneck radius, 1.2 Å; bottleneck tolerance, 3 Å; and maximum tunnel similarity, 0.7 Å.

Phylogenetic analysis

Protein or nucleotide sequences of aquaporins genes were aligned using MAFFT 7.221 and fftns speed-oriented method [**87**]. Multiple sequence alignment was trimmed using trimAl 1.4.1 to reduce errors/uninformative sites in alignments. Automated1 option was run to determine dynamically the optimal method for trimming the alignment between gappyout and strict method. Sequence-based phylogeny was then inferred by maximum likelihood methods using IQ-TREE 2.4 [**88**]. Evolutionary model selection and parameters were determined by ModelFinder [**89**] implemented in IQTREE. The best-fit model selected according to akaike information criterion scores was then selected. The reliability of node in maximum-likelihood tree was estimated by ultrafast bootstrap test (1,000 replicates). Tree, rooted at mid-point, was visualized and annotated using iTOL 7.2 (*https://itol.embl.de/*) [**90**]. The files used to construct the phylogenetic tree, along with the file containing the color code corresponding to the different fungal phyla, are provided in Supplementary Files S2 and S3.
