# Supplementary material for: Major Intrinsic Proteins in Fungi: A Special Emphasis on the XIP Subfamily
Source: J Fungi (Basel). 2025 Jul 21;11(7):543. doi: 10.3390/jof11070543 (PMC12300952; doi:10.3390/jof11070543)
Supplement: Supplementary file 1 [file jof-11-00543-s001.zip › jof-3752183_Supplementary_File_S4.pdf]

Supplementary File S4 - MIPs from the *Chytridiomycota* and *Opisthosporidia* phyla (MycoCosm portal of JGI), along with XIP sequences from one representative fungal strain per phylum where XIP sequences are present. The assignment of the AQP and AQGP subgroups is putatively predicted based on an alignment that includes the characterized orthodox AQP, AQGP, Yfl054-like, Fps-like, "Other" AQGP, and XIP sequences of *Trichoderma atroviride* (highlighted by an asterisk) [**25**].

>Croqu845*
MLSTLRSACLEGSASLIMVWFLGIFMISLSTANLGIHGSAYLAISVAIAVALLVHILGPLT
GGHINPIVTLSTCITGLTPPTKGMAYMSAQMTGATIGGILLVISLGPRSTAIENYGCYF
NPSPSFNVIHASVFEFMGTFAIISMMYGFGINAGATITQGGPKLSPLLNGITLGVYVF
AASGFAPENSYLGTVGFPARCWATSVGTFKFKATDWIYWIPSILASIAHGLAYRFLNP
FGEPKVGTILGHDAELGSSREPKVIIQIDGPQISKTDFAASASPRREKAFDGPQIGPR
RRSIYT
>Croqu6585*
MELLPIWKASIFEGLGSLILIWFIGIASPVFTLNSPVGVGAGLAFLYGSAVALIMFALKPI
TGAHINPFITLSHLLVGTEKSLFRGGAYILSQMIGAVIGGGFVAGALGSDAVALSNGG
CVVDSGNTGMFKPQQAVALEFMAALVLLVLAHSKFSQSQPHSTSLTPFLYGLSVGLL
NFCTSGFAPHAGYIGSFGFPNVCFGLSMGIMKFQPVHWVFWIPGFAAVILHGAIYRF
LKPYDSQNDSENSEMTGHKNIIVPFPSSVFQRDQKGSGQGFETFGNSSVV
>Mycal1170102*
MPESPGILTEKALEANSVRSPRDSKAVNNLSLRIDPPFTMTDFPGSFVRIVQEGQPS
FVPRRSHTLVAYFTHGWTSAEIWKAALIEFWAVACMTYIGGLIGITVMSFGTNIVAPY
VGIITVFLLALWISAAAPGSGGHINSTITFAVLLTRLMSFSRGVLYLAAQLAGSALAGG
LLRASFGLERAIEVSGGGCKLARVAGSVSPTQAFVIEACCSTALLFVAFGIGLDPRQ
QMLYGPALGPVSVGLIVGLIVFATAGLAPGYPGALMNPNRCFAFAVARGDFQDQWI
WWTGPALAGPIHSFLYTLVPPFAFIRLWTFANTHAFFADIIGDIALSILEDNLLNSFKAP
NHGESEGLNICASAT
>Dacru2420693*
MAEPTGKIPNFKSASDTYIAQPPHARAPLLQDRPFAGRLGGNQEFNIAPNDPNYET
VKDHTPDASPMFSWRESFDLRAFGNAELWRQSLFEGFAISLQVFLTGLIAIGLAPAIT
ETSIGALFPSIFGGVTNAIIIPLFIFAVGPVSGGHLNPTITMTTFACRLATFPRTVLYVLF
QVAGATVGGFLIRAGLGVKTDVVPGCYIDPSQVTPGQAFVLETMTTLNLVILAVGVG
LDPRQRGVFGPAFGPILIGAALGITSFGSGFMKAGYSGASMNPARCFGLMAQSGRF
TYHYIHWLGDMTAVMIIGFFHYTIPPYKLQREIP
>Felpe277600*
MSNAKGLRQRTAVSVPAPREVFRGSLSASCRNESIALKRRISRLRTILGFFGTGWTE
RGLYRAGFTEMVAVLSICYLSALLSVTFGQYGIYAPNWVGITNVILVSLFIYANAVPSG
GHLNPLISFSTMLTGLTPFPRAVIYMIAQTIGGAIGGGLIRGTFGLGELNGLYHGGGC
FFDHTAEDTISRGQAFLIEMMSCFILLQLAYGCALDPRTTKAIGPILGPFFVASAFGLIT
FATSGLSPHFAGASMNPARCFAFGVASGNYQSEIYLSPFSALNVAPMVILVRTSGRI
NPHELGVFRGPGIQNGRSRRAYLARIST
>Artol52763*
MSVAIEANPKRDEGAGEKMETTRHRTIRHSKSVPEPLRAAHIHTTPFAARLGGNQE
FVLDRADPKNAAVLEDIPDAATHMTVKQALDLRGFRQAILWKAAAVEGVGTMLLVYA
TDWSTLSPAAYPPPPDPASESGVFSTASFLGPLVGGVTSAIFIAMYIFCFGAVTGGHL
NPLITIATFFTRLTSLPRAILYVSFQIIGASLAGLLIRASYDSREFKVGGCWLNPAEISV
SSAFTNEFSASLVVLFMAFGVGLDPRQRQIFGPSLGPIFVGLAVGTTAFSMAFTRPG

YGGAAMNPARCFGAYVGSSFPTWHWIHWVATIAASVFHGIVYYMVPPWGGFAGKH
ERQLVSDEEK
>Aspac63958*
MDATEAKPTIQPFTGRIGGNQTLVVDRHDPDNAALLKRVPDAAPMMSIKEGLQPGG
FGELDLWKFGFIECVGTMLNVFITAWASIKPTITTTTTPTTAAGIYATTTFLGPTIGSLV
NWLTLTLCIFTFSNVTGSHLNPTITLGTFFARLITLPRAVIYLAAQTLGGTLAGLMLRA
GYGSPNFAVGGCTIDTSLVPMREAFLLEFVFCLLLVFWSFGVGLDPRQGRLFGAAL
SPFLVGMALGTVSWASAFTRVGFGGAGLNPARCFAVYVATGFPGYHWIGWVAALA
AAVGHGVVYFCVPPWSASDVRV
>Aspac64679*
MAVPYLPEYEQEETVMPPATPHHLREHGLEPANSPFAGRIGGNQDFVLDRSDPKNL
AELDRVPDAAPCMNAQEIFDPRGFLSPDLWKFAMLECVASMLNTFISAWVTTHPYA
AMTASTAASGVYGTVTFFSPTFGGLTNLLLTPLLIYTFSPSSGGHINPLITLATFFARII
SFPRAVLYILGQTLGGALAGFALHAAFGDRNITVGGCYIDTTQVPVTSGLVIEFFACLII
VWLCFGVALDPRQAKVFGPAVGPWLVGVVVGVITWGTSFTRVGYIGASVNPARCF
GAYVASEFPGYHWIHWVGPLAASVAHGLVYFVDPLWKDPRAKS
>Aspac44287*
MVPSIRTGQYGTCSQDADKNLERLEIKPTVQPFAARIGGNQSLVLDPNDESNAELLK
RVPDAAPLLSIKKTFSLKAFTDISLWKLSLVECVGSMMFIFITAWLSAHPPSLKESTR
GNTAGVYATSDFLGALVGGISNWLFLTLLVYTFAPVSGAHLNPLITMASFFAQLISFPR
TVLYIAGQTFGGALAGWMLRTAYGSRDFVVGGCNIDSELVQTDQAFVIEFVCCLLLV
FLIFGVGLDPRQSKVYGTALSPWLVGLSLGVISWASAFTREGYAGACLNPARCFGV
YVGSHFPTYHWINWVSPVAASIGHAIVYNIVPPWSAEN
>Perma654005*
MGSESDIELGNTEVSKGELRQRKKRKLSDSGPPVATRPFVGRIGGNQEFTVSPDAS
DFTRVTSKAPDAAANFTWQQSFNLQGFKDPELWKEATIEGIGACAQTYIGGLYSMG
LAPALTATALGPITPAVFGSIATAMLIVLFIFAGGPVSGGHFNPLITLATFTTRLSTFPRT
VLYVSCQCVGGVVAGWILRGTLGLSAEAFRSAPGCFFDSELVTPGQAYGLETMTAF
VLVFIAFGVALDPRQRDTLGPALSSILVGMAIMLCTFASGIARPGYSGAAMNPARCLG
LMSAAEKFDYHYVHWAGAFTATGVNGFLYWLIPPYKNSKN
>Usnfl941042*
MNPVEPERRERLTPATVANFPGSLAANQLGESLETTSYPQRKGRLANWVAYCREG
WEDPGIWKSAFVETLGSFLLCYLNGMIDTTITNFGTPQAPAYAGVVNIFLLTLFIMAFA
PGSGGHINPTITFSTMIVGLTGFSRAILYMGGQLLGAGLAGGVLRGAFGAERAIEFK
GGGCFRNPGTIAAEQALLIETISSFALLALAFGVALDPRQRELFGPLGGPFAVGCSL
GLVSFATAGLVPGYTGASMNPARCFAFAITRHDFTDQWIWWVGPFCGSWLLAITYS
VAPPYHSGTIPPRQ
>Cadma175*
MEDLISDSRGRLIPTQATQFPGSFAPDLRRDSRRYVKSTRWERTVFQYLTDGWDDI
NIWKSAFIEFVGETALCYLSGMIDTVIGNFHTTQPAAYVGVTNVFLISLFIFAMAPSSG
GHVNPVITFATMTAGLTGFSRGVLYLIAQTFGAATAGALIRGSLGGKLATAYQGGGCL
LDTKVLEVGQAYLIESMTTFIMLFLAFGVGLDPRCGQMFGPKMGPLLVGCVLGLTAF
STIGIAEGFPGANMNPARCFAFAVARKDFRHQWVWWAGPITGTMAQTIVYHIAPPY
HREKALERAASQVQSSPGKV
>Xylhe044*
MQKEKHISAMEQKNAVTEDSHVSDHGQVDVPSDIANFKGSFVRNGLGAQHEPKIV
PANKGQVAQWNAYLLDGWDCPGLWKGAFIEFIAATLLIYLSGLIDTTLTNFDTVIIAPF
VGISNIFLLALFIMAAGPGSGGHINQTITFATLLTGFTGFSRAILYMTAQTAGAALAGG
VLRGAYGKARIEKYHGGGCFIDPNTVSSGQALLIEVVSSFTLLFLSFGVGLDPRQAR
LYGAYIGPLAVGCVLGLVTFASAGIVPGYTGASMFPGRCFAFAIARGNFSNQWVWW

VGPIGGSFLQAFTYWVAPPYHSAPVPDAARERKETMA
>Xylhe054*
MATIEQEFIPTRLRRASSSTYSYSTAFYDPPYAGRLGANQEHAVASDDPEYAEIVART
PDAAPFLTWREALLDWKGFGEVGLWKAAVWELWGTMMLVVLTGAVSIGLGRISSP
YIYHSTIIPALIGSAATTIILPLLIFAAAPVSGGHLNPLITISTFFAQISTLPRTLLYVVFQM
LGGVIGASFLRAGLGRRLNPPETGGCTIHSEISYGSAFALELIFATTLLFLAFGTALDP
RQNKSFGPALAPMLVGLAFGICGLASGLLRAGYTGASLNPARCFGLMSASSFTHRF
NTYHWIHWIADVLAAGLNALVWALVPVFRRKI
>Lipor342385*
MSSSEKAADSNGELRQRRVPLRTTRKNESDNAQFQGSFAAGTRQEQWTVVKEPG
TWNTICHYVTYGWNKKTVWKSAFIEFIGTAFLAYITAMTSITVVNFQSPFLPAYVGVVI
AIYISLFIYATAPSSGKCGHLNPMITFSTLLAGLIEFPRAVLYILAQTGGAALAGGMIRG
CLGEATTEKYRGGGCFLNTSTADSLSIGQAFLLETIFSLICIFMAFGVGLDPRQAQLF
GPSIGPAMVGVTVGVLIFTSAGLGFAGFSGPSLNPGMCLAYSVARRDFENQWIFWI
GPTAAAFVHAAIYRTVPPFRLVQISDD
>Geopy171*
MTSQNFTQENKSIQPIPRSFSKFRSQDNSIPPTSERIVDIPNFKEDLKYNYDRPVSC
GSTNSGATLIPTINSRFAAANSTNDDAIHIDMPKKKTNDLQDIEMYSFENDEPKEEYL
SLREMFDKKELLKIHVWRQACAEFVGTLCLEFWIGAVVVGTLVSDFTLKPFLIGFGH
LPILTLLILVIGPISGAHINPLFTFATMFTRLMSVPRGLVYIAFQIAGGTVGALLLKAVTS
SDYIEKTNLGLCNFDSKKINAGDAMLAEAIFDFGLLMVLFLTAFDPYSKKNFSPKVAA
FIIGSTFATLAWLSGGINSGWGGASLNPVKCFAYSVALSDFSDHWAFWVAPLLASSV
YALIYNILAPYAPLSSTRSRRNKTVRA
>Geopy098*
MPNKNQSSLDVADGFRFEERIPEDPVPIKQHGIIEEPVSTINLKSQERSMTEVSIDSV
VTLTSDFPKDKNKLQMISIPEEIKKTCVIDIPSLKNTTVPVEEEFLSLRAMCDYRPLYTI
NFWRQVCVEFLGSLLLQFFVGAAIVGTIVSGFPFPSLLIGFAHLPIIAVLIIAIGPISGGH
INSLITLATMFTRLMSLPRGLIYIIAQLFGGIMGAFLLKTITSEKFTKQTKLGLCNFDTR
EISPIGAMIAEATFDFAIILMVFSVVFNPHCKKNFTLKEVAIIIGSAFGLLVWLSSNLNLG
WAGASLNPVKCFAYSVVVSDFAHHWTFWVAPILASMLYGLIHNIISPFQNLF
>Basme033*
MESSRLSPPDGFVSQGATELRPHETALNVGVPLSNEHLASLEQHITRSRQASLNAIP
HRKSLSGVPTPEFHPDVIADEDHLSYEQMFDWSEFRNPAIWRSGVHECFLMALYVL
VSCGVTVWAVQVGGPVVSLDVGLGVFVTLTLYIMGSASVSGAHFNPCITWTAVFTR
LCSVPRAIIYLGMHCIGAMLGGAMLRAACGEAVTEATKLGSWGFKPEIVSVGQAFC
LEFFFSWSYVWLTWGTAIDPKQQKVFGPVVGPICVGLALGLNIYVSGGLTDGYGGV
GANPAKFFGVAVASGNFQEHWIPWIAPLLACMVHGCMYWVAPPHHEAEKRKKD
>Basme565*
MSATDANQNEAMTTVMVPVANETTGPLSLHVSHCRQASLKPSPHKKSLSRQPATD
DHKRKGSDADHLTFEQMFDWSELLDFSIWRSGIYVCFLMMFYVLMSCGVTVWAIRI
GGPVVSLDVGLGTFGTQTLLALSATAIPGSQLNTMATWTAVGARLCSVPRGLIYMF
MQCVGAILGGAALRASCGHIVTEATKLGSWGYNEEVVTMGQAFCLEFFYSWAYAW
LTLGIGLDPKQRKVFGPVIPGTCVAIALGLNIYVSGGLTTGYIGVGGNPGKYLGVAVA
SGNYQQHWIPWFAPLCASIVHCSLYWIASPYHEYVSS

>Triat283564_FspLike
MCHEAIGGSLWEDRLPHAGIDETISSSRQNVRGSQEDTERGSLRRRSRTSRRSTF
RSVAPLTTAGQGTQFNLAGPSESQQLRSAHEPFVHPGYGDLNPSYEQPNNTKPIW
SLAKPLPRVVRPGMVPTKNELLESCINAELPAENSQKLGLDVDPNELEKGRIDKSAD
VRKMAAQVTDARVQRENNFIKTILASDAESASGDVAQLVQTRSSQRRATITRPPVQ

SPLDTVEESVSDHLSTASKKRRSRESQRLDEAEGEIDLGPDELIEGNRSLETLRIDQ
EAYPEDLHPLVQNLVEDEIHNNHTVWSVVRTHHREALAESLAVFIQLTVGFCADIAVT
VANAGNPNTTAWAWGFASMMGIYISGGVSGAHLNPTITVMLWFFRGFPKRKMPEY
FLAQFIGAFCACFAAYGVYYVSIQHYVSSGTGTDQEIMNCFVTNQRNPYINVPTALF
TEFVGTMCLTVVVLALGDDQNAPPGAGMNSLIIGLVIVCLTISFANQTGAALNPSRDF
GPRLALLALGYGSELFTNPYWFYGPFAGTLMGSFTGAFLYDFFIFTGGESPVNYPL
DRTQRALRKSGMKWRRRLHLTPKEEEDKVV
>Triat90169_AQGP
MTTRELNRLHTHDLELGTTEATQKHVPRNVVPPRRISQRRLDFEHRRPRWLRECIA
EATGVFMYVLPGIGAIATFTLNAASPIGASAFGSLFAIGFAFALGIAFAIITCAPTSGGH
FSPAVTICLCIWQGFPLKKVPYYIFSQLVGGFLAALVLMGIYHQQINEMKELLLAAGK
PLVANGAPASILCSFPNPDQSMGYVFFTEFICDCFVGFIIWAAIDPANPFISPAVAPLMI
GLAYAAMAWGFGDVTLTMNMARDLGPRIVAAIFFGKEAFSYKNYSPIAILVSIPATMV
SSAFYEFVFRDSVSVIQSGHAVHEDGDEALVRHITRTTTVEGIDERRGDYKS
>Triat39327_AQGP
MSPSLEEEQLAWSRIREYCMDAFSEFFGTMVLILFGDGVVAQVVLSGSTKGDYQSI
SWGWGIAVMFGVFVGGKSGGHLNPAVTFANCLYRGHPWRKLPVYALAQLLGAMV
GAAIVYGNYKSAFDAFEGGAGIRTVTGPTASAGVFCTYPAPFMTRTGMFFSEFIASS
ILMFCIFALVDPNNVNAGHMMPLALFFLIFGIGACFGWETGYAINLARDFGPRLVSYM
IGYGHEVWSAGGYYFWIPMVAPFCGTAFGGFLYDVFLYTGNSPINTPMLGLTRLFR
PRKNVWSNTRPSAIDTKV
>Triat31598_AQP
MDSLRIFKQRSVSGDNNRNRDRFHPSTIQKHLIASAGEFVGTFFFLWFAYAGSMQYI
KQATLSPPSGGISDTTVFFIAHVYSFSLLVNVWAFYRISGGLFNPAVTLGMCLAGTLP
WVRAAFLVPAQIIASMCAGGLARCMFPGDLAVANSVLSSDTSIVRGLFIEMFFTAFLV
FVVLMLSAERSKDTFIAPIGIGLALFVAMLAGTSYTGASLNPVRSFGCAVATPSFPGY
EWIYWLGPFMGALVAAGFYRFVKLSHYEEANPNREETDHPTGDQP
>Triat43816_AQP
MPANRIHTLSKRYEKALVIGVGEFCGTFMFLLLSFMGAQVALENNPAADGHKLEPAT
LLYIASSFGTALAVNVWVFYRVTGGMFNPAVTLGLVLVGAVKPLRALIILPTQIIAGIAA
AATVSALLPGHLDVTNSLGGGTSTAQGLFIEMFLTAQLVLTVYFLAVEKHRATFLAPV
GIGVSVFIAHLAGTNFTGTGINPVRSLGPAVVTGRFEGYHWIYWLGPCLGALLSFAV
YSLLKALEYQIANPGQDAGDPETANEEALAVEEMGTEGLAIVRSRTRSGSAASEAR
YKRDVAMRRGSAVDGSAVSAADAV
>Triat6990_AQP
MRRPSASRDTVRNELVVVFGEFYGTFMFLLMSYIGTQAAIDNNSPGNPEAPLFPFS
LMYIAASFGTALAVNVWVFYRVTGGLFNPAVTLGLVLVRAITPLRGLLVFPTQIVAGIA
AAAVTDALLPGPLLVANKLSSGTSISRGLFIEMFLTAQLVITVYFLAVEKHRATFLAPLG
IGLAVFIAHICGTNFTGTGINPARSFGPCVVTSFTGYQWIYWAGPFMGALLAFAVYSI
LKWLEYHNANPGQDDDSNVKRNPAGFAITSNDRQYSSSTTGSKPHTPTGHNPRDS
GIAQTNSAPQGFQAV
>Triat319992_XIP*
MEPLQEKPLPADRERGTTQARAASSAVSLSNSSTPAATHKMDLAAFDGSFAPLVRP
QAVRLTPWYRRRDYFVGQWFEPALWRSAVVELIATCCQVFVSGQIVATISTYGTPQL
GAYIGISNLVLIATFIYAVAPASGGHMNPTITFAAVLTGLCSVPRGLLYLVGQTAGGALG
GGILLGIWGKERAIAVRGGGCWYDPSQANPGQIYLNETFASFVLLFLAFGVGLDPR
QAALFGPRMGPALVGASLGLVSFSTSGIIPGYAGAQMNPAKCFGNGIARMDLSYQW
IYWFGPAVAGIMMGILYNLIPPHHAELCKRKSREMSREMTSDSMAERAEASVIASA

>jgi|Parsa1|3432
MPKIDAHYMRRVAAEFLVTYLFIFTICCNGLNDARSGGVGGDVRSGVSTGFAAVALI
YCFGGLSGAHFNPAVTVGAMVGQ
KMDFLTGSIYIGVQTLAGMAAVASLIALFPEAKGATLKALQLSVPEGVSTWQVVAME
FVLSFMLVMVIYGSAMGLKTVIT
DTDIESQDEKAELISINKMRLNFAPIAIGLALGYLCFLGGSISGGAFNPSRATAPVILE
GKFTGLWMYWIGDCAGGAAAA
LVYNYVLTN*
>jgi|Rozal_SC1|10481
LAKALLGEFLATFIFLFVVMATGVNVYRLGGDAVGIIIGGVGTGFTAVAVIYSFADVSG
AHFNPAVTFGTMVMGKTSWIK
GAGYIGVQLFAAVIATAFLAVVFPEKGNVVFSEVLVSVYKDTNVINAVVTEFILSFILVY
VIFAVAFDTVDSTNAAPIVT
GNALKDRSIGRNLTIYTTSGSTKAGFAPIAIGFTLGFLCFIGGSTSGGAFNPARAFGP
AILTGKFDLHWVYWLGDFAGAA
FAGFIQSFLAHKPKQASGSARI*
>jgi|Gorhay1|191639
LIRAIAGEGLVTFLFMFVVMAISVNNGRQSTPENLVLGCLGTALVSVALIYSFADVSG
AHFNPAVTFATMVTGKTSVRKG
LGYIGIQLFATVFATLALMAVFPGVAGQSAAAIPAYLTVDVNNEAHLAQAFFMELILTFI
LVYVIFATAFDTVDTKATKI
HADGTVSGGQNMTIYTTSGNTKAGFAPIAIGFTLGFLTLIGGTVSGGAFNPARVFGP
ALVSGNAWDNMWLYWIADFAGAA
LAGHAQAFFAHEAQQHSAAIKTNKAVVKSAPQ*
>jgi|Gorhay1|261371
MSSPRQFENQPIAEQGPKPRRQGRHGSHDPHNEDVSEYANFTVDPDDFAAGIAEF
IGTWFYTFIGLVAIQSAVTNGFTSP
NTPLTPASTLLISIGFGFAWVICVGMAAPMSGGHINPAITIGVYCTGRMPPIRALIYIAF
QITGACVGAALAEVVTPGGF
LGYNEVNPNLNVGFTVLAEILFTFILVATYLSTEFDSEMDLGFTPLFTGLSILVGYLGL
VPIDGGSMNPARSFGASAVAK
NWEAHWIYWVGPIVGAILAVLLRNGFAKTAKRTRSSTRTSAW*
>jgi|Gaesem1|55274
MNRRNPMHILLQPMRGTKNVPFARIWRAVLAEAIATMFFVFFVGASVEIPAKVNKSQ
DLNAGSIFTALTQGLTIIAVVAA
TARLSGGHINPAVSFCLMWTRAMPLATAMSYIAAQCVGAIAGSALFYACIGDLHSTL
GATTPHMINTFQACLIEFMITSM
LLFVVLGTAVQGSAAEPGMKPMAPIPIGFSIVVGVLLAGNLTGGSMNPARSLGPAVV
SGTWTKQWVYWVGPLLASIVVGS
LYKLLFLSAPVSKSLVKDLGPSEITVHGGRAMSEAEFIDRTADDEGLVTNDPAYRGT
GQGMGQQDGYAFSSRLPLGCSRW
LIPIF*
>jgi|Powhir1|454234
MSELHKLTDMPGNPSSPRGGQVSVDIERQGLTQNAANNDDDDEDAPRPIDTLNDA
PGYFSWNPLDWIKEFQGEFEEFSWA
MTEKRRVLLRAVFGEGLCTFLFLFVVEAVAVNNGRSENDTNLTLAAVSTAFVSIALIY
SFADVSGAHFNPAVTFATMVTG
KVSLRKGLAFMFIQLVAAIFSVVALLIVFPGPHPGYASIPASIVVSVHSTAHLGQALAM
EIMLTFILVYVIFATAFDTVD
TTNAVKVKTKGDKKAAARDEAVGRNLTIYTTSGNTKAGFAPFAIGFTLGFLGLIGGSV

SGGAFNPARVFGPALLSGNWSH
HWIYWIGDFIGAALAGWAQHLFAHEATQTSASQKTKKGAAQALESERAAAIAS*
>jgi|Powhir1|460009
MSGHHEINIHDPDQRNLHPMRSDHTLSPSGGRSGGGSQRRNDHSRKQYSPIVRD
ALAASVETLGTFLFLFAAYGAVNAAS
AAAIAEGGASGVVGVAGTMMIATSFGLSLLVVAWALYRVSGGLLNPAVTVALVISKAI
TPRRGAMFVIGQLIGALAAGAV
VEGLFPGPFTGANTLKNGISITQAFFLEIILTALFTIVVLMLAVEKSKHTYLAPIGIGLAL
FLVHLVAVPYTGCSVNPAR
SFGASVFEGEWNNHWVFWLAPILGGVLASLFHMAIRRIDYESLNPGQDAAHDGEK
QQETKEVEGTRERD*
>jgi|Gervar1|402949
MSSALDKQASNETVVDMSNEQTSPAPHAVNNAKVTDHVVEQRGEHVDEYLRYDS
QHASALYKLKHRYREELAEFFGTFIM
MIFGNGVCAQVSLHGGKNGEYLSISFAWGFGVLFGILAAGGISGAHLNPAVTIMNAM
HSGFPWRKVPGFAAAQILGAFTA
AAVVFADYRSAIRAFDGGVRQVTGPQATAGIFATYPQPYLTPAGAFFSEFIATAILAIG
LLSIGQIKSADRPKYYAPVAV
AFLVMAIGMALGAPTGYCLNPARDLGPRLLTLVAGYGTETFSTASYYGCWVTIFGPIL
GAIFGGYVYKGFLDYESVELET
KENKTV*
>jgi|Gervar1|403997
MTELQKLPGSPRGTPGSGHAAIDIERQGLTAPEDDDDAPAGPIDTLNDAPGQFSWD
PRDWIKEFQSDFDEFSWQMTEKRR
VLLRAVFGEGLCTFLFLFLFTVEAVSVNNGRTTADTNLTLAAVSTAFVSIALIYSFADVSG
AHFNPAVTFATMVTGKVSLRK
GLAFMFIQLIASIFSVVALLIVFPGPHAGYSSIADSIVVSVDSSAHLGNAFAMELLLTFIL
VYVIFATAFDTVETKAVKV
KAGDKKGKKAEDEAAGRNLTIYTTSGNTKAGFAPFAIGFTLGFLGLIGGSVSGGAFN
PARVFGAAMVSGNWSHHWLYWIA
DFMGAAIAGWAQHLFAHEATQTSANVRSKTGAAVENAKQEQLQASAPNH*
>jgi|Gervar1|444884
MHSDHTLAPAAGQGQRRNDHPRRQHSPIVRDALAASVETLGTFLFLFAAYGAVNAA
STAAAAEGGPPGVVGVAGTLMIAT
SFGLSLLVVAWALYRVSGGLLNPAVTVALVISKAITPRRGLMFLFAQVIGALVAGAVVE
GLFPGPFTGANALKNGISITQ
AFFLEIILTALFTIVVLMLAVEKSKHTYLAPVGIGLALFLVHLVAVPYTGCSVNPARSFA
ASVFEGTWDEHWIFWLAPIL
GGVLASLFHMAIKRIDYESLNPGQDAAHDALDAGTEPLPRSMTVDAGR*
>jgi|Gervar1|471764
MHPFRDRQPTSLNHNAGGNGDVVIEDVPPSVTTAETTAAATRRPSLSQKAYNKAR
HAANFVAKDISGPKVPRPRFARAVG
AEFLGTMFFVFAVAGSVVIPANLGLTSGGAADKAASLLVTAFTQGLALAAMISATGPM
SGGHLNPAVTTSLMVVRTIPIA
RGFAYIGAQFLGAMCGAGIFKAVIGVDETGTLGATVPQVGSGQAWGMEFMITAVLIY
TVLGTAVHGGANGIVKALAPLPI
GFAVIAGVLIGGTVTGGSMNPARSFGPAVASGTWTRHWLYWLAPLSAALIVGLVYKA
FFLSTSITHSQARRAGLLPSNNV
ITSPATGDGQLRASGAGGPAMRSAETVINMPPTEAYYRPEENLSEADLIAHTGEDEI
EAIA*

>jgi|Fimjon1|531984
MSSPDRHHEINIQDSDQPRRLQPGSSEHTLSPAGRNRQFRNDAPKHNYSPFMRDV
LASSVEALGTFLFLFAAYGAVNAAS
AASAADGGKDATGGVAGVLMIATSFGLSLLVVAWALYRVSGGLLNPAVTVALVISKAI
TPRRGAMFVAAQLVGALVAGAL
VQGLFPGPFKGANTLKNGISIAQGFFLEVILTALFTIVILMLAVEKSKHTYLAPVGIGLA
LFLVHLVAVPFTGCSVNPAR
SFGASVFEGTWDDHWVFWLAPVLGGVLASLFHLGLKRLDYESLNPGQDASRESE
KKQESREVEHAHGGGAGRV*
>jgi|Fimjon1|549771
MSLESQISKDTVVDVAEHDVINHVKLSPLVRLRRQMREELAEFFGTFILIIFGNGVCA
QTSLHKNTGEYLSINFGWAIGV
LMGIYVSAGISGAHLNPAVTIANAVHSNFPWRKVPRYCAAQVLGAFMGAAIVFANYH
SALDAFDGGVRQTTGDLKTAGIF
ATYPQAYLTTVGAFFSEFITTAVLMIGLFAIGESRHADRPKSHSAIAVALLILGLGICLG
APTGYAINPARDFGPRLLTL
VAGWGTDPFTVSNYYFWIPIVGPITGGIFGGFVYQAFTEYDNAPILWTKSSVRRLLP
NARICTHNMYAVYIR*
>jgi|Fimjon1|267760
MTKFAKSISTLLEPMPGATNVPRARKWRALVAEFIGTMLFVFFVAGSVAVPSGLKAD
PVASLLFIGLTQGLALAVFVSVS
ANISGGHLNPAVTLALLVARAVSPITALLYIIAQMLGAVAGAGLYLATMGTAHADHLGIT
TPTNFDGGNVYLFEFILTSV
LMLVVFATAVHGSIQGHVVLAPIPIGMTLVIANFLAANFTGASLNPARTFGPAVVASAY
SDVAFAWREQHLYWLAQLSAA
VVVGVLYKMIYLSAPATKAQVREAGLLGNSAATVQGMSEAELIRRSARDEAPAIIDIP
DIPEQTSPRSTRNDSLPTSASP
LVHTHPRSALAHTVVHIDSVTETAAAGLPRRTSIPPMVGVGTQRQTKDALTETAAAG
LPRRTSIPPLVGVGAQRQTKDAL
PVTSAGSARSRMPSAGIDRSVIDYSRAREVVRLSFGAEEIRV*
>jgi|Polagg1_1|460649
MASSSTLPTDTVSTGPKQHSVLVKELIQAGGELFGTFLFIFLAFATIQASLPVQGPLF
NLFVALGFGVGLLLAIALTYRI
SGGVLNPAITIALAALRLFSLRKSALYIVAQLLGAMAATGLTSLLFPGPTKGANVIVNP
AFTAVQVVFLEAIATCVLVAS
VLFLAVEKSRVTFLAPVFIALTVFLDHIILIPIDNASLNPARSFAASLTTNTWDSHWIFW
VGPILGGLIAAAIYKIFKVA
RYEQLNPNQDASE*
>jgi|Triarc1|583962
MGTVNVPFSRVWRAVVAEFIATAFFVFFVAGSVQTPTIVSSTSSIPVSPANFVITGFT
QGLAILCLVATVANISGGHLNP
AVTACLMVVRNMPFLTGCMYIVAQILGGIVGAAFYKACLMSNAGTLGSTVASDVMN
PFRTILTEFFITSMLLFVVLGTAV
HAGTRHTGIKPLAPIPIGFAIAIGVFLAGPLTGASMNPARSFGPAVVSGTWKWHYAY
WVGPLVASLVVGCLYKIIFLSSP
ITMDQARAAGIDVGNPSSATLEGDHLAPGTMHWQGGRSISEAEFIARGASDEHVVE
IPPQHTEMRTSA*
>jgi|Triarc1|645370
MSLKSQQQQQQQSMSIHDRKSVVSQDTLDTRRRPPSSTWRQWTGHWMHILRQE
RAEFLGTLVMLLFGDGVVAQVVLHEGK

GDYLSINLAWGFGVLFGIYATGGISGAHLNPAVTLANAVFNKFPWRKVPSFMLSQIL
GAFCAAALVYLNYQPAIAAFDPS
LTTTGLKSTAGIFATYPQPYLSTAQAFFCEFFATALLIVGLFSIGESRSPAAPSSHGPL
ACGFLVMAIGMAFGAPTGYAM
NPARDFGPRLFTLVVGYGTAPFTVSNYYFWIPIVGPLLGCIAGGLAYSCLLSFPPPPP
KSLVICDLDAETCHPLP*
>jgilGlopol1l565400
MTASTGFTEIKDTNEISPTRLENGAASSPDAEFSEVPPDELWSWNPVDWYHGIKKEI
AEFDFQMTEKKRVLIRAMFGEGL
VSFLFLFIVMATAVNNGRQENPENLVLGCISTAFASVALIYSFADVSGAHFNPAVTFAT
MITGKVSVKKGFGYIGVQLLA
SIFATLALMAVFPGVGGNGPFSIPEYVVVDVDAQTHLANAFFMELILTFILVYVIFATAF
DTGVKVQADGVKDDHSGNNL
TIYTTSGNTKAGFAPLAIGFTLGFLGLIGGTVSGGAFNPARAFGPTLLSGKGWKNHW
IYWVGDLIGAGLAGFAQSNFAHE
AQQHSANLKTKGKVV*
>jgilGlopol1l625454
MPYSLAIRCLSEFLSTAVFICLGNGVVANELLAATKGQSMGFGFVSIGFGMAVGFAA
TMFGHISSSMNPALIVAAWVKGS
LPFSEIWPLMFSAIAGAFFGQVLVFLLYLPHYNVVPQLEDFETITESVNVLKEHQSDG
ESALRLSSHKHSMASLKNNDKS
TKENEIYLAAVEADQAKKLSTCSTRPAIDAPVYNFICEFIGTFILSFGGSLIDDRLTIDP
DTLTTSIYSRGLSPLITSFF
IMTLVMVLGGPTGFSANPARDLGPRIAHALLPINGKGPSEWRYSLISNSAVFCGAIV
GALAYKLFRSTVY*
>jgilGlopol1l119922
MSPTQEINIEAGNNQTKKFEPKELLGDFVAAIAELIGTTFFIFLSLTAVQSAVTLGSTN
AGSLSASVILLIATAFGLSLM
VSIAMVAHISGGHLNPAVTIALAAFGLVSIPRAAMYIVAQCIGATLGAVFTIMVTPGPLL
GYNAVNPAVGQSGAIVAEIL
LTFVLVITVFMTAVEGKMNPGVAPMFIGLSVFVIHLAGIPIDGTSVNPARSFGAALISG
KWADQIVFWLGPIIGGLLAAG
VYKFFKVFA*
>jgilEntlut1l1198390
MKLLIQSFTECLGMFLFIFLALGGVQSALYTSGANTLAFIQIAMCFGFGLAVAIFFAYRI
SGGALNPAVNFGLLVAGVMD
PVTALAYTVAQMAGATAACGAVAGIFPGNSFKGANAVFAPTTIVQGFFLEMVLTFGLV
ITVLIMAVEKSRAAFYAPMIIG
VYVFIAHLLAIPYTNTSINPARSFGASVITGIWTDHWVFWVAPLLGGAIAGGVYRFYK
AVDYESLNPNQDATEKV*
>jgilEntlut1l1293151
MSSRFNQQTEFQQIQDDDIPIMQTSSQSEQPLSNKQLTRKESEIVVIAGDSPADDDA
PGYFSWNPIDWYKDLKKEFDEFT
WVLDEKKRVLLRAVFGEGLVTFLFLFTVEATAVNNGRQESPENLVLGALSTALCAVA
LIYSFADVSGAHFNPAVTFAVIV
TGKTSLKKGLMYIGIQLLASILATSFLMAVFPRPHDGTFSSIPSSVVLDIDPTASVANA
FFMEFILTFVLVYVIFAVAFD
TVDTSNNVKEAGAVLGKTKDNEAGKKLTIYTTSGNTKAGFAPLSIGFTLGFLGLIGGS
VSGGAFNPARVFGPAVLTGNFH
NNWIYWVGDFLGAALAGWTQHLFSHEAVQNSHSIGTKAGAGRT*

>jgi|Entlut1|1299480
MKIAIQSATEFIGTFIFLFIAFGGVQSALRSNGPEQLALNPTLAFIEISLCFGLGLASAIF
FAYRISGGALNPAVNFGLF
LAGAIDPITLGAYTIAQTLGATAACATVAFVFPGDFKGANEVFTGTSGAQAFVLEALLT
AGLVLTVLFLAVEKSRVTFWA
PMAIGLYVFTAHLVSIPYTNTSINPARSFGAAFVSGNWDNHALFWFAPLSGAALAALL
YNFYKYVEYEKFNPGQDADGTA
VTTSVNVEVKETIKETVKVLKED*

>jgi|Ganpr1|119683
MTDKRRVLVRAVAGEFLVTFLFLFVVMSVGVNFRRANPTEADATLGGISTGFTAVALI
YSFADVSGAHFNPAVTMGTIVT
GKVSVKKGLWFIGIQLSASIMATLWLQVVFPPHRGFNASEIAVVDFGDKVVIGRAFA
MEATLTFILVYVIFATAFETVDT
NNKVLVKPVADGAQKSATVNPNGAGREVTQDASVGKNLTIYTTSANSKSGFAPLAI
GLTLGFLCFLGGSVSGGAFNPARV
FGPAIVTGNWNNHWVYWIG
>jgi|Chylag1|183517
MSYFAKHTPAENNTNGASSTSVAVPVDQPHSAPLLPSTAADRAALTKELVQAAAEFI
GTFFFLFISYGTIQTAAGHVDKL
TTISLAFGLALTTGVWLTYRISGGALNPAVVFGLLILGKITPRKAALYVAGELLGATFAA
FIVALIFPGAWSNFGGANTV
FHPSSSIQAVLLETILTSGLVLVVLFIAVEKSKATFIAPLLIGLYVFLAHLPSITLDNTSLN
PARSFGAAVLSGYWSFHW
VFWVGPFLGASLAAGIYTFFKHFNYETLNPEQEDDHAAARIRLH*
>jgi|Powhir1|545247
MSTRLSTENAVPPHSNTLAANRQPSFKKVVTSAKSAAHFMAKDISGGPTPRMRIAR
AMCAELIGTMLFVFAVAGSACIPA
ALGETKGAGLLITALTQGLAIAALVSATGPISGGHLNPAVTTSLMVVRAISFWTGISYIV
SQCVGAITGAALFKLAIGSA
LSGTLGATVPIDTAGRTFTMEFMITTILVFTVLGTAVHGGANGVIKALAPIPIGFAVLIG
VLIGGSITGGSMNPARSLGP
AVVAGMWTEHYLYWAAPLGAAIVTGTLYKAVFLSSPITLRQARVAGILPGPTNNVGR
NPDGIHASHATVLEMGNPGMSEA
DLIHAEAIDPNPIREADLYETPAVGSSPPDHVVVDMAEIPVVRPIPIRPPAPAPTPMTE
ITEERTSATATPPPFSDYKSR
ERKPSQGGDTRHISVNTQERTSATATPPPFSDYKAHEREPSQGVDTRHISVRVSRA
QIEWAALNAQ*
>jgi|Spipu1|712
MSTLDPQESKETIVDMAPEIQDKVTHKNKSGHLAYIRHQFREELAELLGTFILIIFGNG
VVAQVVLHNNKNGEYLSINIG
WGLGVLFGIYASGGISGAHLNPAVTISNAIHKGFPWRKVPGYVIAQILGAFLAAAVIFG
NYHAALDAFDGGDRQTLGPKA
TAGIFSTYPQPYLSTVAAFFSEFLGTAILMIGIYACSEAGNSAAPDGYGPIAVSLLVMAI
GMALGSETGYAINPARDLGP
RTLTAIAGWGSEPFTAYNHYFWIPIVGPIVGGVFGGYVFRALSAYKNSDEGIY*
>jgi|Hyacur1|661765
MLYSGFLARRSAIGCSDGVHRHRAGPVNGPPPPHGLPTAPAPASGGRCGARPVLR
MAKFELKLPALFGGGGAPPAGKRAA
RKGPRLPRDLPVAKYPPGLRGKMAKEAVQGLGELIGTVLFLFCGFGALESAATIEAN

AGMTNLTVAFGFGIGLLVNCWVF
FRFTGGLFNPALTFAVFASNPRTFGWRRFIIYTAAQLAGGIAAAAMADACFPTNNVV
TVNFPDNGTTAGQATVLELIGTY
LLTSVVLMGAVEKSRATFLAPLAIGLTLFVVVLILNPFTTSAINPAKALGSAVVSGFWL
NHWVYWVGPYSGALLAVLFHT
ALRMLDYQSMNGGVDDFDFTALRPAEPGPGEDEDELIAPEADEEADAEPKARQAG
QPGKREAGGESAVGLLDDAAEA*
>jgi|Hyacur1|673571
MSFVLFGLKLTPGKWEPQHKLLKEFIQACGEFVGMAAFVFCGIGGANSALRLNNPG
NLDPNGENPVTLILNDTAVQLIAW
CFGMGLCINIYAFYRYTGGVFNPAVAFSLFAIGAMSWTKFLMYVIAEMLGATVGAAFI
RLLYPADVNGNRILGVNGVAPG
VGVWQAVGIEAFGTFLLCFVVLMMAAERNHSRPLAPLVIGLTVYACHLLLIPYTGCS
VNPARSFGAALAAVNFTYQWVFW
VGPLIGSLVAVAVYGFAKAWDYAALNGAQDEDETVERILSGQEKIAHGATVAQAADF
AEGECRLWRGRTQDGQRVQPHLR
SPGEPRRPRRG*
>jgi|Hyacur1|685027
MADQLEPAEAGGGGGRRPSFSTPTDVPLKQSGYAQDDVKDLRLDLYRAQGRTTIV
DQYGAKDGAVAQRMAAAEPVGDGKD
GPAMPLSPLPAGTMPPEADERRPSAAPTAYSHYRGWLRFRMDYKGYFGEAIGMAV
TIALALSAVAQVELAGGEKGGILSV
AFGFGFAFMFGIIVCGGVSGGHLNPAVTIVLATFRGFPWRNVPGYILAQTVGAFIGAA
VVYLIYYPAFAAFDNGCYGVPN
TNTCSKASAGVFGTYPPPPFSWYSGFINEFVGTAILVTIVFATADSKNAPPTAYGALA
VGFLVFQMVNSFNWVSGPALNP
ARDFGPRCFSAIIYGSAVFSSTSYYFWVPIVAPVLGGLFGAGLYDFGLMQDIAPTSP
AMYEFIP*
>jgi|Hyacur1|705531
MVVNWREHEWRWWITEALGTFWLVFLHAGLSIISRTDANPDPVRRLSFGGQAMGV
GLSLVLLIYSYGGLSGAQVPGYWLS
QGVGAMTAAGALWGFFGGDDAQLGSNYPEGLLKGSPERCFFLEFLLSFLLQSVALL
TASRAMSVGANAALAVGATLGVCT
LVGGPFGGGSMNPWRSFAPGILAPRYRGWLWIYCCAPLAAAPVTALVRIVFREGKV
EDGERTAATGEGETEP*
>jgi|Hyacur1|714381
MSAPQPYAKLPSGAILEMQPTPVGRSGFQAVREGDGGAAGGAPESEARSWFGGA
KTDPANFEMNEKRKVLVKAVAGEFLV
TFFFLFSVMSAGVNSMRSGNGEAVLPAIVTGFVGVALVYAFADVSGAHFNPLVTFAT
MVTGKTSVKKGLAYIFVQLLASV
GATLWLRVVFPAPVVAGAPTAAQLAVVSVPPGSNPFNAFLMELTLSGILAYTVFATAL
DTVDTTNRIAFAPSAPLDQSVG
RNLTIYATTGNSKAGFAPIAIGLCLGALCLMGGSSSGGVFNPARAFGPALVSGRWEY
QWLYWIADLSGAAVAALVQSLLA
HKAVQSSGEATAGSS*
>jgi|Hyacur1|714401
MAALEENRRLLNDPADSAATFPAEAPKRRLGWGLDEPGLAKAVLGEFVVTLLFLFA
AMANGVDGARSGGRDALSGAVVVG
FIATALIYSFADTSGAHFNPLVTTALLLRGKVTVGKAAAYIGVQLIAAVVATLLLLVVFPA
ASEGFPSAAQLAVVAIPRG

SSVGRALLMEIALSAILAHVVLATAAFPADKAKAPFAPLAIGLTLGALSMLGGTSSGG
AYNPARVFGPAVVTGTFDGHWV
YWVGDLAGAAAGAGLQLLIMSLA*
>jgi|Hyacur1|423580
MSSADGAKVEAVPMPLEGAKDSTAVAVPTTLAPKASRTATSTAETRGGTAAAFVNQ
YGDKDQVREILQHPQRVPTRTPTI
GALELPPATEIVPKPRTYLAKFKYAIAPWKDLIAEFFGTMVLIIFGCGVVSVVVLSGGA
NGDSNTIYWAWGWAVMMGILV
SGGYSGAHLNPAVTLSLFVWRGFPARKVVPYWGAQLLGAFTGAAVVYWNYYPAFV
HFNGVTNGGAFVVNGFQTGTQVPTA
GVFATYPADFINNGNAFVSEMIGTALLLFVIFATGDAKNAAPTAYGALAVGALIMALG
MSWGWLTGYALNPARDFGPRAF
SAIVFGPKVFQAYGYYFWVPVVGPLVGAVVGGGLYEIFLMSEADEA*
>jgi|Obemuc1|856281
MSKHRSFATKLGLQSFAEFLGTFLFLFLSFGGVQSALKTNSKTLEENPTIAFIQISLAF
GFGLATAVFFAYRISGGALNP
AVNFGLFVAGVMDIFTLTAYTLAQVAGATAAAAAVAFAFPGDFKGANELFDGVSYAQ
GFVIESILTAGLVLTVLFLAVEK
SKITYWAPFQIGVYVFVAHLLAIPYTNTSINPARTFGAAIVTGNWNSHISLFWAAPLA
GGALAGGIYRFFKLAGYETLNA
DQDSDGCKSVVAVKEE*
>jgi|Obemuc1|856278
MSSIQSTVISETHKITPVPSIAGGNRGVSKAVAISPAIKKLLIQSATECFGMFIFLFLAL
GGVQSALRTNSADELALNPT
LAFIEIAMCFGFGLSAAVFFAYRISGGALNPAVNLGLLVAGVMDPLTFVAYTVAQTIGA
VIACGIVSAVFPGDFKGANQL
FDGVSTGQGFFLELILTLGLVLTVLFLAVEKSKITFYAPMMIGTYVFIAHLLAIPYTNTSI
NPARSFGASVAAGVWNDHW
LFWVAPLGGGALAGAIYRGYKYVEYETLNPGQDADH*
>jgi|Obemuc1|918556
MSSRFNQHSEFKQLNDDDIPSLPPAGGSVQKETAIVVAAVPEPDTFDDAPGYFSWN
PIDWYKDLKKEFDEFTWEMDEKKR
VLLRAVFGEGLVTFLFLFIVEATAVNNGRQEVPENLVLGALSTALCAVALIYSFADVSG
AHFNPAVTFATIVTGKVSLKK
GLMFIGIQLIAAILSTSFLMVVFPRPHDGSFHDIPSSVVLDIDPTAQLVNAFFMEVILTF
VLVYVIFATAFDTVDTGNKV
KVAGGGAAKDNEAGKNLTIYTTSGNTKAGFAPLAIGFTLGFLGLIGGSVSGGAFNPA
RVFGPAVLTGNFKNNWIYWVGDF
LGAALAGWTQHLFAHEAVQNSHAVGTKANAKNN*
>jgi|Chyhya1|555718
MSSSFKQHSDFKQLNDDDIPIIAGSDAVKETAIVVTAEPPADSFDDAPGRFSWNPYD
WYLDLKKEYDEFTWEMDERKRVL
LRAVFGEGLVTFLFLFIVEATAVNNGRQENPENLVLSAISTALCSVALIYSFADVSGAH
FNPAVTFATIVTGKVSLKKGL
MFIGIQLLAAILASSFLMIVFPHPMQGSYRDIPSSVLVNLNPGVPLVHGFFMEVILTFV
LVYVIFATAFDTVDTSNKVKV
AGASKDSEAGKNLTIYTTSGNTKAGFAPIAIGFTLGFLCLIGGSVSGGAFNPARVFGP
AVLTGNFSNNWVYWVGDFLGAA
LAGWTQSLFAHEAVQNSHAVGTKNNAK*
>jgi|Chyhya1|573990

MSRVSTKTSSAAPFQHSFAAKVVIQSLAEFLGIFVFLFLSLGGVQSALATNSAEQLAL
NPSFAFLQISLCFGFGLAVAVF
LTYRISGGALNPAVNFGLLVAGIMDPITFIAYTIAQVAGATAACAVVDITFPGDFKGAN
QVFAGVAIEQAFLLESILTAG
LVLTVLFLAVEKSKITFFAPMLIGIYVFIAHLLSIPYTNTSINPARSFGASYVSGNWDNW
DIFWSAPMAGAATAALIYKF
FKVLSYESLNGDQDSDVAVVVVKTKTSGDKVKAN*
>jgi|Chyhya1|589949
MQRSRVGKLALQAATEWLGMFLFIFIALGGVQSALKTNAADLATDPTLAFVEISLCF
GFGLATAIFFCYRISGGALNPAV
NFGLLVAGVMDVKTAFVYTVAQLLGATVACGAVSAIFPGDFAGANQVFDGVGTIQAF
LLEAILTFGLVLTVLFLAVEKSR
ITFLAPLIIGIYVFIAHLLAIPYTNTSINPARSFGASVVSGSWKDHWVFWVGPLSGGYV
AGLVYRLYMIADYTELNAGQD
ADSQ*
>jgi|Chyhya1|589937
MFIFIFISLGGVQSALKTSAASLATNPTLAFIEISLCFGFGLATAIFFSYRISGGALNPAV
NFGLLVAGAMDPITMVAYT
VSQCIGATAACGAVAAIFPGKFAGANQVFPGITVTQAFFLELILTFGLVITVLFLAVEKS
KITFFAPMMIGIYVFIAHLL
AIPYTNTSINPARSLGASIITGTWDNHWIFWVAPLCGGAIAGLVYRFYKIVGYETLNA
GQDADHVV*
>jgi|Chyhya1|600576
MRFAFPRKTTPHTSNQTSTTSTNNMAQTDAAKLAIRSATEFLGMFIFIFIALGGVQSA
LKTNAASLALNPTLAFIEISLC
FGFGLATAIFFAYRISGGALNPAVNFGLFVAGAMDISSAVAYSVAQMAGATAACGAVS
LIFPGKFAGANQLFDNTTIAQA
FFLEMILTFGLVLTVLFLAVEKSKITFYAPMMIGIYVFIAHLLAIPYTNTSINPARSFGAS
VITGMWKDHWIFWVAPLCG
GALAGGVHRFYKMVEFEKLNPGQDAARIV*
>jgi|Chyhya1|629995
MSSSFKQHSDFKQLNDDDIPIIAGSDSVKETAIVVTAEPPADSFDDAPGRFSWNPYD
WYLDLKKEYDEFTWEMDERKRVL
LRAVFGEGLVTFLFLFIVEATAVNNGRQENPENLVLSAISTALCSVALIYSFADVSGAH
FNPAVTFATIVTGKVSLKKGL
MFIGIQLLAAILASSFLMIVFPHPMQGSYRDIPSSVLVNLNPGVPLVHGFFMEVILTFV
LVYVIFATAFDTVDTSNKVKV
AGASKDSEAGKNLTIYTTSGNTKAGFAPIAIGFTLGFLCLIGGSVSGGAFNPARVFGP
AVLTGNFSNNWVYWVGDFLGAA
LAGWTQSLFAHEAVQNSHAVGTKNN
>jgi|Chyhya1|707208
MSKTKRSQNSSMPRISTDVLPAPYQHSFAAKVVIQSLAEFLGIFVFLFLSLGGVQSA
LATNSADQMALNPSLAFLQISLC
FGFGLAVAVFLTYRISGGALNPAVNFGLLVAGIMDPVTFIAYTIAQVGGATAACVVVDI
TFPGDFKGANQVFAGVAIEQA
FLLESILTAGLVLTVLFLAVEKSKITFFAPMLIGIYVFIAHLLAIPYTNTSINPARSFGAAY
VSGNWDNWDIFWSAPMAG
AATAALIYKIFKAAGYESLNPDQDSDVAVVAVVKTKSSGDKVKTN*
>jgi|Enthe1|493319
MPYSGPYSLGTRCMTEFLGTAMAIFLGDSVIANELLPQTKGHGMGFGWIATSFGMS

FGVSIIMFGYASAHINPAMALALW
LIGRLSTGDFFAIAAAEIAGGFIGAVGMYITYLPHFRTVPEPKSNDEDSPLLRTRDTID
ASALRIASYNTKSSPSEPPRD
FAQRLNEAKYYLTSENTDPEQLFNILSVGTLDLAGVEVAFPKTDLEAAPSAAAVTAAS
PANADQPHRPIQRRHSIQVAEM
QRRLRQLESSLDSTSAATLPSGDAATQAPIVMQVGDDLNVKPTTKQQQQQQPAK
RVAVAEPSTATLSRMTRSQALQKAA
IKADQAAKLSAFCNRPAIYLPIHNYLVELIGTLVLILGALLLDDRFASAKPAAAAILGSEA
ATLVFENGLAPFLIAIFIQ
VCILSLGGPTGFTANPARDLGPRFAHWVLPIPGKGKSEWNYSLYVNAGALSGGALA
GLLYMGIKQVRDNHL*
>jgilChytri1l1087710
MSSRFNQTSEFKQLNDDDIPVIAGSTSASSPTKDHEAIIVAPSVDEAETFDDAPGYF
SWNPIDWYKDLKREYDEFTWTMD
ERKRVLLRAVFGEGLVTFLFLFIVEATAVNNGRQENPENLVLGAISTALCSVALIYSFA
DVSGAHFNPAVTFATIVTGKV
SLKKGLMFIAVQLLAAIVATAFLMVVFPHPMTGNDSRLLDIPGSVVVNIDPSAPLVNA
FFMELILTFVLVYVIFATAFDT
VDTSNKVKVAGAANKDSEAGKNLTIYTTSGNTKAGFAPIAIGFTLGFLCLLGGSVSG
GAFNPARVFGPAVLTGNFSNNWV
YWVGDFMGAALAGWTQHLFAHEAVQNSHAVGTKGNSS*
>jgilChytri1l1101017
MTLPRKNRSSSPPMVKTSSSATFSFPVKVALQSFAEFLGTFLFLFLSFGGVQSALAT
NAEQLEINSSLAFLQISLCFGFG
LTAAIFFTYRISGGSLNPAVNLGLMVAGIMDPFTFFAYTVAQVAGATLACTAVDFAFPG
DFKGANQVFAGVRLEQAFVIE
SILTAGLVLTVLFLAVEKSKITFLAPLLIGIYVFIAHIISIPYTNTSINPARSFGAAMVTGV
WENHDIFWSAPFAGAACA
ALIYKLFKIVGYEQLNAGQDSDGKAVARRLSGDKVTKATKEE*
>jgilChytri1l1160442
MFLFLFLSLGGVQSAIKTAGTTLAVNPTLAFIEISLCFGFGLASAIFFSYRISGGALNPA
VNFGLLVAGVMDPITFVAYT
IAQCIGATIACAAVAVIFTNNDFKGANQLFPGVSVAQGFFLELILTFGLVITVLFLAVEK
SKITFWAPMQIGIYVFIAHL
LAIPYTNTSINPARSLGASIIAGIWDNHWIFWVAPLCGGALAGVVYRFYKFVDYESLN
AGQDADHNV*
>jgilChytri1l1160886
MFLFLFLSFGGVQSALATNAEQLVINPSLAFLQISLCFGFGLAIAIFFTYRISGGALNPA
VNFGLMVSGIMDPFTFVAYT
VAQVAGATLACTAVDFAFPGDFKGANQVFAGVRLEQAFVIESILTAGLVLTVLFLAVEK
SKITFLAPLFIGIYVFVAHII
SIPYTNTSINPARSFGAAMVTGVWENHDIFWSAPFAGAACAALIYKVFKIVGYEQLN
AGQDSDGKVVVRRVSGEKVAKIN
KEE*
>jgilChytri1l1166425
MSQSDVTKLSIRAATEFLGMFIFIFLSLGGVQSALKTHAAELSINPTLAFIEISMCFGF
GLATAIFFSYRISGGALNPAV
NFGLFVAGAMDAATALAYTVAQLAGATAACWIVSLIFNNNDFKGANQLFAGVTIAQG
FFLEVILTFGLVITVLFLAVEKS
KITFYAPMMIGVYVFVAHLLAIPYTNTSINPARSFGASIVTGIWKDHWIFWVAPLVGGA

LAGLVHRLYKFVEYEDLNPGQ
DAAEKLV*
>jgi|Clarep1_1|50747
MCGSLPEPKSVKPADLKSSSYTPIATTRRLGNFQALALQSLGEFLGTFFFLFIGLGSV
PAAAKYDHPAASFITISLAFGL
GLAISVFIFYRISGGVFNPALSVALFATGHFSLVKAGAYIVSQLAGAEVAALAVKGLFS
AGEGVTYSLANKLNGIGPIQA
IVAEALLTAPLGLLVYFLAVDKARVTFLAPFFIGLYVATAHLVLSPYTGTSLNPARSLAG
SLVSAEFPDHYVFWAGPILG
ALLSAGIYKLYSHFDYTQLNGDQDAESDGAIRLPA*
>jgi|Clarep1_1|798110
MPSNEASSSSYVHPAATTRRLTEFQALLLQGLGELLGTFFFLFIGLGSVPASLKYDQ
PAASFLTVSLAFGFGLVIAVFIF
YRISGGVLNPALSVALFATGHFSAIKAGIYIAAQLIGAQLAAHAVKGLFPVPELDFYSL
ANKLNGLNAFQGIVAEAILTA
PLGLLVYFLAVDKARVTFLAPLFIGLYVATAHLVLSPYTGTSLNPARSYAGSVVSGDW
SDHYVFWLGPILGALLSSGIYK
LYTHFDYVQLNGDQDADAPAGSIRLPA*
>jgi|Clapol1_1|92520
MMPAPVGDVKTSGSMPRSTTPTKQQHHQPQRQQRPLAVTLALQSLAEFYGMFVFI
FLSLAGVQSAVFQGPAPTALLQISL
VFGLALTTAVWLTFRISGGALNPAVNLGLLVAGAMDPVKAVAYTFAQCAGAVAACAAV
AAVIPGSHGKAFFGANAVQAAS
DGSAACSPAQAFVLEAVLTMGLVLVVLFMAVDKHRATFMAPLMIGLYVFIAHLISIPYT
NTSINPARSLGASVVSGTWND
HWIFWAGPFTGAVVAGLIHRFYKAVDYESLNPGQDADEAEMV*
>jgi|Clapol1_1|621676
MTPLVTQSLAEFYGMFLFIFLSLAGVQSAVFQGTATTTALLQISLVFGLALTTAVWLTY
RISGGALNPAVNLGLLVAGKM
DPVKALAYSVAQCAGSVAACAVVAAVFPGSHGKAFFGANAVQAAADGSAVCSAGQ
AFAMEALLTMGLVLVVLFIAVDKHR
ATFIAPLVIGLYVFIAHLISIPYTNTSINPARSFGASVVSGTWNDHWIFWVAPMTGAAV
AGLIHRFFKALDYDSLNPGQD
ADSEFLTV*
>jgi|Clapol1_1|805788
MFKHHHTTTTNNGNTPSTTEHVTYQTVVTSTTTGASGAGGYSSHSHPHEPLPHGG
PVPVIAPHVYTPPSDLTKELVQAGG
EFVGTFFFLFIAYSAIQASVKHQEHFVLSISLAVGLGLTCGIWFAYRISGGALNPAVVF
ALYLLQKITQRKAALYVAAQV
LAATVAAFVVSAVVPGSWGGHFKGANQVFAGTSLLQAFFLEALLTAGLVLLVLFIAVE
KSKATYLAPLIIGLFVAIAHFA
SIPYTNTSLNPARTFGSALIAGNWNAHWLFWIAPLSGAALAAGVYKVFKHYHYELLN
LGQEDDHAPGAIALP*
>jgi|Rhihy1|712857
MLGMFIFIFLALGGVQSALYTNNDAARALNPTLAYIEISICFGFGLAAAIFFAYRISGGA
LNPAVNFGLFVAGVMDPITL
VAYTISQSLGATLACAVVSQVFPGTFKGANQVFAPTTTAQAFFLEVVLTMGLVLTVLF
LAVEKSKVAFWAPMMIGIYVFI
AHLLAIPYTNTSINPARSFGASVVSGIWNDHWLFWVAPLVGGAIAGIIYRLYKAVSYE
TLNPGQDADH*

>jgilRhihy1l718259
MSSRFNQNSEFQQLKDEDIPVLGGSSPVKGDAIVVVPDVPAPDDDAPGYFSWNPI
DWYKDLKKEFDEFTWTMDEKKRVLL
RAVFGEGLVTFLFLFIVEATAVNNGRQEVPENLVLGALSTALCSVALIYSFADVSGAH
FNPAVTFATIVTGKVSLKKGLM
FIGIQLIASIIATAFLMVVFPRPHDGSFHDIPSSVVLDMDATALVVNAFFMEVILTFVLVY
VIFATAFDTVDTGNKVKVA
GAAGQKNNEAGKNLTIYTTSGNTKAGFAPIAIGFTLGFLGLIGGSVSGGAFNPARVF
GPAVLTGNFRNNWIYWVGDFLGA
ALAGWTQHLFAHEAVQNSHSVGTKANAQKQQN*
>jgilRhihy1l762465
MTHNSVEDDIAIAQQTTAAVKFVVKNWGFATKLAIQSFTEFLGTFLFLFIAFGGVQSA
LKTNASALETNPSVAFIEIALT
FGCGLAAAIFFTFRISGGALNPAVSFGLLVAGVTDVFVFTAYTVAQVIGSILACVLVAVA
FPGDFKGANQLLDNTTFVQG
FIIETVLTMGLVLLVLFLAVEKSKITFWAPMMIGIYVFTAHLLAIPYTNTSLNPARTVGAA
VVTGIWDHHFTLFWFAPFT
GAAIAGGLYRFYKVIDYHTLNPGQDSDGSVSENFEEILEQKLEKSKFLRMFFKKDE*
>jgilRhihy1l762466
MTASPTLAFVEISLCFGFGLAAAIFFSYRISGGALNPAVNFGLFVAGVMDIFTLTAYTIA
QVSGAVAACTLVDIAFPGNF
KGANQLLNETTYVQGYIIESVLTAGLVLTVLFLAVEKSKVTFLAPLMIGIYVFVAHLISIP
YTNTSINPARTVGAAVVSG
AWDYHFTLFWFAPFTGAAIAGGLYRFYKAVDYETLNPGQDADGSNVNVVENISIQQ
TVVVEEDAKVKEE*
>jgilRhihy1l846567
MAKEHSKFTKLALQSATEFLGMFIFIFLSLGGVQSALKTNDSAARALNPTLAYIEISM
CFGFGLAAAIFFSYRISGGALN
PAVNFGLFVAGVMDPLTLIAYTIAQSAGATAACGAVAAIFPGDFKGANQVFAPTTTAQ
AFFLELILTAGLVLTVLFLAVE
KSKVAFWAPMMIGIYVFVAHLLAIPYTNTSINPARSIGASIVSGIWNDHWLFWVAPLA
GGAVSGLIYRAYKFVEYETLNP
GQDADHSPIDDSKA*
>jgilEntlut1l407549
MRSTWRLQYAETVSATILTRLLARPMELAVGLIRSWRCLQILRYKYILLRPHHNSNCT
AGNILMPYSGAYTLGARCLTEF
LATFVAISMGEGLLANELLPTTKGHALGFGFVCLGFGMSFTVAIQMFGYASAHLNPA
MCLALWIRDKLSFTDFLALSVSE
MAGGFCAGVFVYLMYMPHFKTLPEAQGSQEENLLRTRDQLDPQALRLASYSTKPN
YSRPATTEKPATFTQRLAEIKYYLL
NQTPNTDEVLDHLIGGTYALHGSEVPFPTGTESVSGDAEIALPRISQDAKALKRRHS
LQVADMQRLLRKMEKELAGSAPY
DDAATNVAMDSSQASGSASGVVVVPKVQKKITREEALGRAAILADQASKLSVFATR
PAIYLPLHNVFVETMCTFFLIFGA
SLIDNRFDMISNEANAQAGNIDIGAFLVGMMIMALILGFGGPTGYAANPARDMAPRL
AHFVLPIPGKGSSELVYGVLIAV
GALIGGAIAGGLILAVENIPVQA*
>jgilChyhya1l609060
MYTGSYSLGTRCLTEFLATFCAIGFGEGIIANEVLPTTKGHALGFGWVAFGFGMSFT
FAILIFGFASAHLNPAMLLSLWI

RDEISAGDFFALSAAEMCGGFCASVFVYLVYLPHFRTVPEPSGTQDENLLRSRDHI
DPSALRFASYSTRSHAFSNSNTLT
SHTGPKKSLGQRLADARYYLLAENFDTDPETVITHLIGGTYALHGAEVPFPSGTESV
GRNTPVPAGTADLEAPVQFIPGK
ASLKRRHSLQVADMQRLLRKMERELESKESPTFVNANPPNSAAGDLLLPSTVPGK
SSDVPAPASNISFRNSLETNHRIQP
KLSKQDAIYRASIIADQATKLSVFATRPAIFLPIHNFFVEMIGTAALIFGASLIDSRFKMI
KDPQIQQAENISLKPFLVG
MYIMVLVLGLGGPTGFAANPARDMACRLAHLVLPIKGKGSSEMWYGMVINTGMLVG
GALGAGFVIAVDQIRGWTIQ*
>jgi|Clarep1_1|18212
MSTSATASDAAAAAEAAIRGAINPVISSRPSTPPSVPLSHTTASSPPLSPTAPSSGQP
LSTLHQDMPPPDYNEAVRSGTS
NTSAAPSAPPLSAFQSPPARPPAAFSSTSPTVGRDQPILASDFPSPPAFTYPIPSST
QQRSVDGAMSRMSSNSSDHRSAP
PSGVFPPRSQPLSHPPVHMTSSLGSSQPSTVSFLAESTTIPNRIPSEDDIPGSRRSA
APNSHLPNSHAQALPQQNSLPQR
HSITINSLPNAPASQSARSIPNRGFRGFANGILRETASSLTSLSNAIGGRTVASEPDAT
VLRRMPSAGNRGAILPTHSGD
GPSERPLRSIVFQDALNGRSTNNVSRNITSADFRRSTAVEPLLRDQDSEIASTIGDS
HGRLDVGSSAPRNGTIDPIPTRA
QLQLQDVNMSRQFSHSTVRLGGSQYKRNELRKGYSTTRLSTIRIGHAPTSWRHRL
RPLLAEMCGTFLLVLFGTGTIASLV
LSDALFGLWELGAAWGFGTALAIYSTASISGAHLNPAISFAMALFKNHTAFTWVQFIT
YSISQLIGAMLAATLNYELNYQ
FLESFESRMAITRGSPQSMRSAMIFNCYFPNPQVQGVDGKFTDEDLARVAQLVSPL
LAFLAEALGAAVLVFVVFCINDHN
NTAVPRGSEPFFIGFTVSVLIAVLGPTTQACINPARDFGPRLVATAAGWGSVALPGPN
NGFWVYLAGPMVGAILGGGLFF
LVIKKVSMPHSLLLDDCLSFGCSTGR*
>jgi|Clarep1_1|766740
MNPVISSRPSTPPSVPPSHTTAASRPLSPTTPSNGQQSSPLHQDIPPPDYNEVFRT
GSSNTSAAPSAPPLSAFQSPPARP
PAALSSTSPTVGRDQPILASDFPSPPAFTYPIPSTQQRSFDGAMSRMSSNSSDHRS
APQSGVFPPRSQPLSHPTGQVTSS
SGSSQPSTVSFLAESTAIPNRIPSEDDIPSSRRLGVSNSHPPNSHAQLLPQQSSLPQ
RHSITINSLPNAPASQSSRSIPN
RGFRGFANGILRETASSLTSLSNAIGGRTVSSEPDATALRRMPSAGNRGAILPTHLG
EGPSGSERPLRSIVFQDAPNGRS
TNNVSRNITSADIRRSTAVEPLLRYQESEIASTIGDSQGRLDVGSSAPRHGTIDPMPT
RTQLQQAHLQDVNMSRQLSHST
VRLGPQYKRNELRKGYSTTRLSTIRIGHAPTSWRHRLRPLLAEMCGTFLLVLFGTGT
IASLVLSDALFGLWELGAAWGFG
TALAIYSTASISGAHLNPAISFAMALFKNHTSFTWVQFVTYSISQLIGAMLAATLNYEL
NYQFLESFESRMAITRGTPQS
MRSAMIFNCYFPNPQVQGVDGKFTDDDLARVAQLVSPLLAFLAEALGAAILVFVVFC
INDHNNTAVPRGSEPFFIGFTVS
VLIAVLGPTTQACINPARDFGPRLVATAAGWGSVALPGPNNGFWVYLAGPMVGAIL
GGGLFFLVIKKDDEETEWVD*
>jgi|Rhihy1|700680

MGYTGAYTLGQRCLTEYLATFCAIGFGECLLANELLPNTKGHACGFGWVSFGFGM
SFTFAIQMFGYASAHLNPAMCLALW
VRGQLDFTDVLALSASELAGGFTAALFVYVMYMPHFRTIPEAHPPSQDDNLLRTRD
HIDPQALRMASYSTKPQYSTRPSQ
GPKTLAQRIADARYYLLAENFDRDPEGVIEHLIGGTYALHQAEMPFPTGAESMSRR
GSSTLMRESTTTAATAADAENGAV
SFIPGKNELKRRHSLQVADLQRLLRKMERELEYTTPAISDLRDQAAGDLAVPVKAAS
TSSQQSPSGSPHITFKNVQEAFK
LGIKPKVSREEALGRAAIAADQATKLSVFATRPAIFLPIHNFLVELLGTFFLIFGASMID
ARFDLIADPAVNQGSLIFIK
PFLIGMYIMMLILGLGGPTGYACNPARDFMPRLAHYILPIPGKGSSEIWYGVVINVAA
LLGGILAGGIFMAVDKIHEMH*

>jgi|Caupr1|10172
MTEKRRVLLRAIFGEGLCSFLFFFIVMACAVNNGRQEQPENLVLGCIATAFCSVALIY
SFADVSGAHFNPAVTFATLVTG
KVSINKAFAFITIQCLASVLATAFLMVVFPAPYGSSKTADRWDIPATVTVDIEASTHPV
NAFFMEMALTFILVYVIFATA
FDTVNTEGTPAVVGEDGVENGGRVGTKLTIYTASGNTKAGFAPLSIGFTLGFLGLLG
NSVSGGAFNPARVFGPAMLSGNW
SNHWLYWIADFLGAALAGWTQHFFAHEATQDSASSKARIKAKVVA*
>jgi|Caupr1|14914
MGRELLQALAELIGTFFFLFTAFSGAAAARRQSSLTGASGAQGGDSDYMTAAISFGF
GLMVACWVTLRISGGALNPAVVL
ALALVRAMPLRKAALYIVAQICGGFLATGLVTALNPGSFLGANALTPGVSRAQVMFLE
AIMTALLCLVVLFLAYEKHSST
MLAPTIIGLTVWVSHIVLIPYDGCSINPARAMAGALGQHNWHDMWIFVLGPFIGSFIA
AGIFAFFKHFKYEQLVNPYADI
DALYLHQVADPRGQRDHVLVAGRDPDGPPGC*
>jgi|Caupr1|23600
MGFLSATTGGLPTWSPKTDLDARGEPLHSPYSLYEKRYSDVAGGYAACDNGHGDI
DYADVVPDSSLRKDLFQALAEFVGV
TLMMFTTWMANSAAARQNESTGEGQSYMTGSIGSAFGAAMACFCVMPISGAALN
PAVVVSLVVAGIMPLRKAGLYIVAEV
LGGIMAAWMTIVFTPNTFIGANVVAYGVSRLQAFCLEVLMTAFLCLVVLALVTDKGPT
MAGPVIIAIFVWLAHLDLIPFT
GCSMNPASSIGGAIGEGQWANLWLFIFGPSIGGVVGSAIFMFFKYHGFARVENPFS
DRPISVV*
>jgi|Hompol1|6727
MSDQPKKSNFAEVPNDIEAAPLQGFDEIKDDGDVVPESERFSWNPYDWYLGIRKEI
EEFSFTIDERKRVMFRAIFGEGLV
TFLFMFIVEGTAVNNGRQSNPENLVLGALSTALASVALIYSFADVSGAHFNPAVTFAT
VITGKVSVRKGLAFIGIQLLAS
VFATLFLFAVFPGVNGNGPSSIPSFLVVNIDSDAHVAQAFFMEVILTFILVYVIFATAFD
TVDTKGVKVATAPGAANKDQ
NAGANLTIYTASGNTKAGFAPIAIGFTLGFLGLIGGTVSGGAFNPARAFGPTLISGQG
WGHHWIYWIGDFLGAGLAGWAQ
HLFAHEAVQHSASVRTKKGAMEAAVLAK*
>jgi|Gaesem1|38509
MSDFQKLSDDHTVDMPEGHASSSDAKHTEDPAILATEDDEIAPFDDLNDAPGQFS

WNPVDWYSEFQNDWKEFSWVMTEKR
RVLFRAVFGEGLCTFLFLFVVEAVSINNIRQKQAEGLVLAAVSTAFVSIALIYSFADVS
GAHFNPAVTFATMVTGKVSLR
KGLAFMGIQLFAAIWSVVACMIVFPADVNAGFKSVAENIIVDVNPEAHLFRAFCMEIIL
TFVLVYVIFATAFDTVDTSNE
VKVKEKNAAGGEKQISPVTAAADKSVGRYLTIYTTSGTTKAGFAPFAIGFTLGFLGLI
GGSVSGGAFNPARVFGPALLAN
KWNHHWLYWSGDFIGAALAGWAQHFFAHEAVQQSGTSAKTKKGAARKQVETAQT
*
>jgi|Batde5|10971
MFRAIFGEGLVTFLFLFIVEATAVNNGRQANPENLVLGALSTTLASVALIYSFADVSGA
HFNPAVTFATVITGKVSVRKG
LAFIGIQLLASILSTLFLFAVFPGVNGGGVWNIPEFLVVDIDSSAHLAQAFFMELILTFIL
VYVIFATAFDTVDTKGVKV
ATAGGEKNKSSGNNLTIYTTSGTTKAGFAPLAIGFTLGFLGLIGGSVSGGAFNPARAF
GPTLISGQGWTHHWIYWVGDLT
GAALAGWVQHFFAHEAVQHSASVRTKTGALEAAAAERVALNANIANSQAI*
>jgi|Powhir1|435475
MRDVVAATVEALGTFLFLFAAFGGVNASQTAAGGDSPGAAGAMMIATSFGLSLLVV
AWALYRVSGGLLNPAVTVALVISK
AITPRRGAMFVLAQLIGAICAAAMVEALFPGPFTGANSLKNAISVTQAFFLEMILTGLF
TLVILMLAVEKSRGTFVAPVG
IGLALFLVHLVAVPYTGCSVNPARSLAASIFEGTWSDHWIFWVAPVLGAVLASLFHLG
IKTFDYETLNPGQDAQNDGEKQ
WVTREVEADSDV*
>jgi|Powhir1|456297
MALDERQTSKDTILEMNAEEADIKGNMVEPIVDQKGEHREEIVHTTRFGVLTRLRRR
YREELAEFMGTFIMIVFGVGVVA
QVVTHPGGSKGEYLSINLSWGLGVLFGVYAAGGISGAHLNPAVTLGVAAHCGFAWK
KVPGFIIAQMLGAFCAAAVVYLNY
RDAISAFDGGVRTITGDTSTAGIFATYPQPYLSTGGAFFSEFFGTAILAIGLRSIGEAK
SADAPKGHSPIAVALLVMGIG
MSLGSETGYAINPARDFGPRLFTSIAGWGGDVYTAANNYAWIPIVGPVLGAQAGHLI
YGFFAEYKSVDSKAVTV*
>jgi|Spipu1|1731
MKIQNLVSPRRDNTSLPTNGNTNGDGIPNEVQGQGRDPMTILKMPMRGTVHVPRS
RIWRAVVAEFIGTAFFVFFVGGSVQ
TPAIIRNEAPATAADFVITGFVQGLAILCLVASIAGISGGHINPAVTACLMVVRSMPVIT
GIMYMIAQFIGAIVGAALFK
ACVASRGGDLGATVPTWWINPFQTILIEFFITSMLLFVVLSTAVHAGVTSSGIKPLAPI
PIGFAVAIGVFLAGPLTGGSM
NPARSFGPAVVSNTWRYNYAFWVSPFLASVVVGILYKIIFLSAPISQEQAHNSGLEFA
PSHVTLDMGSATPEERALASRP
TGISEAEFINRSASNEDICRVPLGVGTAVMRSTGQGVTEQSLHSSPLTGVERAV*
>jgi|Fimjon1|359857
MSEYETQKLSNLGPSSPSRGMASQGLGQDYSVDIERRGLTSVLPDDDEIAPFDDLN
DPPGKFSWNPMDWVKEFQQDYKEF
SWAMTEKRRVLLRAVFGEGLCTFLFLFIVEAVTVNNGRQENPENLTLGAVSTAFCSI
ALIYSFADVSGAHFNPAVTFATM
VTGKVSIRKGLAFIGIQLIASIFSVVALLIVFPGPHAGYSSIPASIVVDIDSTAHLGQAFA

MELALTFILVYVIFATAFD
TVDTSNAVKVKAKGAGQKDESVGRNLTIYTTSGNTKAGFAPFAIGFTLGFLGLIGGT
VSGGAFNPARAFGPALLSGKWSN
HWVYWFGDFIGAGLAGWAQHFFAHEATQTSADQKTKKGAERERAEQAAV*
>jgi|Enthe1|403446
MLRAIFGEGLVTFLFLFIVEATAVNNGRQANPENLVLGAVSTAFASVALIYSFADVSGA
HFNPAVTFATVITGKVSVRKG
LAFIGIQLLASVFATLFLFAVFPGVGNSSANSIPAFLVVDIDSSAHMANAFFMELVLTFI
LVYVIFATAFDTVDTKGVKV
LANGQKEKDSSAGANLTIYTASGNTKAGFAPLAIGFTLGFLGLIGGSVSGGAFNPAR
AFGPTLISGQGWKNHWIYWVADL
TGAGLAGWAQHLFSHEATQHSAAVRTKKGASELAGVSSGEA*
>jgi|Polagg1_1|474611
MSSVVESGSEESETPVVEIDMGESDPSDSHVSCKIRWGLWTHKVKRKLTYYGLAIQ
LSWPHFQPYVAEFLGTLFLVLLCI
GSLSSLVLIGALKGIWELAVVCGTALGLAIYGTAYVSGAHLNPAISLTMAIWQYRTGFT
FKKALLYWVAQLLGSIAAGFL
NYALFNALIAEYEQLNNIHRGQSGSELSAMIFVNYFPNPGYYPQYYTPTTNASSASI
NGPLLTLLSPANALFCEAFGSGM
LSFMAFALTDPDNRAMPRKELAPYFIGLTLTCLIAVIGPITMASFNPARDLGPRFVAAA
LGWGTVAFPGSRIGFWVYILG
PFIGAPIGGGIYLLLLGKRPRSTRSRRLTHSARERQVHYDDE*
>jgi|Polagg1_1|517600
MPTYTTTTTTSSSSTLPTDPFNAPPRFSALTKDLLQAGSELLGTFIFIFVSLGAVQAAI
KTQGDAFNLTVATAFGVALTL
AVSITYRISGGVLNPAITFALYLLNLFELRKSLLYATAQVIGASLATGAVALLFPGDFKG
ANQILDDKITVLQVIGLETL
LTALLVLVVLFLAVEKSRVTFLAPVFIGFTVFVAHLVLIPYDNTSLNPARSFAGSLAAGI
WADHWVFWVGPLLGGAAAAG
VYKFFKYVDYEELNPNQDASSNEHKRIILV*
>jgi|Polagg1_1|525130
MSATYTLGERCLTEFLATTMVMFVGLSVIANEILPGTKGHGMGFGWIAVGFMAAFIIP
LIIFGYASAHVNPASCMALWVM
GKISGWEFLALSASEMAGGFLGSMLMYLFYLPHFRTVPEPSGTHEQNLLRTRDHY
DPSVLRFASYSTKSGLNSTKSLKER
LKEARYYLTNSDLDGRPDQVLDILSSGTYHLHRMEVPFPVNESPNEESAISGLGQT
GKALRKRHSLQVADMQWQLRKLEA
ELAASTNAPVPEEDPNRTARPSEVVPPVRAKTTDDIVAVPASTKVVHDHSRESSSVT
TKEDAIYRAMIIADQNAKLSVFA
TRPSIYLPIHNCFAEAVATCALMLGAILIDNRFSEITGETGLVYSNGLSQFFIGLWIQTL
VLGLGGPTGLAANPARDLAP
RIAHMLLPIPGKGSSEMFYGLLINAAALVGGAVAGLLATAILLIKH*
>jgi|Polagg1_1|581998
MSGFQKMDDFNEVQNYTVEIGDRQPLAAASPTNNDDLALARTETRDNDLEDHPGP
LSLNPVDWVSAIRQDIKDWSWEMTE
RKRVLLRAVFGEGLCTFLFLFTVEATVVNNSRQETPENLVLGCISTAFVSVALIYSFA
DVSGAHFNPAVTFATIVTGKVS
VRKGLAYIGIQLLAGIIATAYLMAVFPKPYDSGYLSTPLAVVVDINSQAKIINAFLMETTL
TFILVYVIFATAFDTVDTT
NSIKVAGDGKDTNVGKNLTIYTTSGSSKAGFAPVAIGFTLGFLGLLGGTVSGGAFNP

ARVFGPALLTGSWNNHWIYWVGD
FLGAALAGFAQRQFAHEAVQSSHAIQTNKANK*
>jgi|Triarc1|588848
MTELQKLTSPGGHTSVDMERRGLTGADPFPLDDDEVAPFDDLNDAPGKFSWNPVD
WIREFQRDYSEFSWAMTEKRRVLLR
AVFGEGLCTFLFLFIVEAVTVNNGRQEQPENLTLSAVATAFCSIALIYSFADVSGAHFN
PAVTFATMVTGKVSLRKGTFS
FKVLGLLVPKRLRPFFSGLAFISIQLVAAMFSVVALMIVFPGPHAGYSSIAASVVVDID
DSTHVARAFFMEVMLTFILVY
VIFATAFDTVDTSNQVKVKPKKGANGVVAPVAKDESVGRYLTIYTTSGNTKAGFAPF
AIGFTLGFLGLIGGTVSGGAFNP
ARAFGPALFSGHWDHHWVYWGGDFLGAALAGWAQHFFAHEAQQHSGASAKTKT
GAADEVKNQETEDRERSAPM*
>jgi|Batsa1|11691
MVDFKKSEFSEIESHPIDIETSPAPETSDMSIVPIEERFSWNPYDWYLGIRREIQEFS
FTIDEKKRVMFRAIFGEGLVTF
LFLFIVEATAVNNGRQANPENLVLGALSTSLASVALIYSFADVSGAHFNPAVTFATIITG
KVSVRKGLAFISIQLIASVI
ATLFLFVVFPGVGGNGVTSIPSFLVVDIDSSAHIVQAFFMELILTFILVYVIFATAFDTVD
TKGVKVNTGNGERDQTSGN
NLTIYTTSGTTKAGFAPLAIGFTLGFLGLIGGTVSGGAFNPARAFGPTLISGQGWTHH
WIYWVGDLTGAALAGWAQHLFA
HEAVQKSSAVRTRTGAIEDARAAAALAKSASISTPINNKSA*
>jgi|Anasp1|228580
MKQYEYSDDAKSNLIKDCVSALGEFIGTTFFVFMGIASVNSAFHAMETRNVDNIAFM
TDITNSTVVNRGYPSGPSPSALI
QIAFGFGFGLMCMVWAFYRISGGHLNPAVTLGLFCSGAVPPIKAVLYIIAQLCGGML
GALFAKALFPTPHDGVTRGANGI
ADDKSVTAGFFLELILTFLLVYVVHMLALEVNNARNVAPFVIGAIVFVGHLIAIPITGTS
MNPARSFGASVVSGEWDDHW
VFWVAPLVGGILASIVYRIFSFFNIQALNGNQDHTTAMEYENKAIAINSTESLPH*
>jgi|Ganpr1|33948
MTASSDLLNPTAFLFAPTHPRTPYSEGVVELLQALGEFIGTWFFVYTGLAGVNAALS
VTATASLDPAAVLSIACAFGFGL
AINVSLWYRVSGGSLNPAVTLGLVLIGKLNVRKAALYIVAEVLGGVVAAAFVNALYPG
GVIGVNKRADGLSVAQAVFIEA
FGTFLLISTVFLTAVEKSRITFLAPVLIGLVVFVLHLILIPYTGCSVNPARSFGPAVVTGD
WKDQWIFWIGPVLGAVFAS
VHYYFITKINYRVFNPDQDSDGLDLVAKSHLANGDAKKAHVASMPSLGANASCETV
V*
>jgi|Ganpr1|490160
MIQEEHIPFDWKNPATWLHAINEYYNDSSRVLLRAVTGEFLVTFLFLFVVMAVGVNF
SRTNISTADATLGGVSTAFVAIA
AIYSFADVSGAHFNPAVTFGTVVVGKTSGIWYIAVQLLASVCATLWLMLVFPVIDGVK
ASDLAIVDVGADASLTRAFFME
LTLTFILVYVIFATAFETVDTSNKVIVNGKESKVGQNLTIYTTSGDSKSGFAPLAIGLTL
GFLCFIGGSVSGGAFNPARA
FGPAVITGVWNNHWLYWVGE*
>jgi|Chylag1|403730
MDSVFTTNQSHHDYVPPEVKVRIHPETLSESLEEKSTSTLGATSSGEKNVTSTAPKT

QQHFTIIIRAMRKLYYFANAYYP
MFQPYFAEFVGTFLFVFLGIGSVSAIVLVGALKGVWEMGIVWGISLAISIYCTASISGA
HLNPAISVTMARCGLYIISQF
LGGLLAGAVNLSVWKDFITEFERENNLVRDILGVRDQPCTTPHIFSPWLAFSVEMFG
TGILTFLAFAVTDPSNKSITRKE
LAPLIIGLTLTLLICLFAPLTQACFNPARDLGPRFIAALAGWREIALSPSTSWVYTIAPLL
GGPCGAAIYLFGLERRITD
DSVVLD*
>jgi|Chylag1|445256|gm1.6697_g
MSEFSEVQSQYVDVEAAEPIDESEIIIAAPKGESDAPGRFSWNPLDWYKEFKQEFE
EFTWTFDEKKRVLIRAVLGEGLVT
YLFLFIVMATAVNNGRQEQPENLVLGAISTAFASVALIYSFADVSGAHFNPAVTFGCIIT
GKTSIKKGLLYIGIQLLAAI
FATLSLMAVFPRPKDGFFKDIPSSVVVSLDGSAHIVNAFFMEVALTFILVYVIFATAFDT
VDTSNSIKVNGKASKDKGVG
KHLTIYTTAGNTKAGFAPLAIGFTLGFLCFVGGTVSGGAFNPARVFGPALITGQFRHH
WIYWFGDFIGAALAGWTQSFFA
HEAVQHSHSIATKQGGQN*
>jgi|Obemuc1|856279
MSKERSQFVKLGLQSATEALGMFIFLFLSLGGVQSALKTNSAAELAANPTLAFIEISL
CFGFGLATAIFFSYRISGGALN
PAVNFGLFVAGVMDPLTLGAYTIAQMIGATAACGAVDAIFPGDFKGANQVFPGTTITQ
AFFLELILTMGLVLTVLFLAVE
KSKITFYAPMMIGVYVFIAHLLAIPYTNTSINPARSFGASVVAGVWNDHWLFWIAPLV
GGGVAGAIYRGYKLIEYESLNP
GQDADHV*
>jgi|Obemuc1|1007969
MGYTGIYSLGTRCLTEFLATFMAIGMGLGMIANELLPNTKGHAFGFGFIAFGFSMSF
TAAICMFGYSSAHLNPAMVLSLW
VRGKLDFTDFLALSASELAGGAAGALFIYLIYLPHFRTVPEARASNTDDLLLRTRDQI
EPSALRFASYNTKPHYASKTTP
DSKKTLAQRIADARYYLLNESFDEDPEGVVEHLIGGTYALHGAEVPFPTGKESLSRK
ASVPALEKGESSNTIVAAGGIKR
RHSLQVADMQRLLRKFERELGGNEPSVHFDTTAGDLAIPARNASPEPEIEASGAAA
GKGELAKINREEALGRAAIAADQA
TKLSVFATRPAIFLPIHNFLVEMLGTAFLIFGASMIDARLEMITDAGFKNGTMPTMAPL
LIGLYIMVLILGLGGPTGFAA
NPARDFAPRLIHYILPIPGKGDSELWYGVIINVGALLGGALGAGMFMACNSIHN*
>jgi|Chyhya1|600580
MSRQQNNKVTKLAVQSFTECLGMFIFIFISLGGVQSALKTSSAELAVNPTLAYIEIAM
CFGFGLATAIFFSYRISGGALN
PAVNFGLFVAGAMDPITLVAYTVAQCLGSTAACAAVSAIFPAKFAGANQVFPGISIVQA
FFLELILTFGLVLTVLFLAVE
KSKITFFAPMMIGIYVFIAHLLSIPYTNTSINPARSLGASIITGTWENHWIFWVAPMSG
GAVAGLIYRFYKMIGYETLNA
GQDADHVV*
>jgi|Chytri1|1165263
MPYTGSYTLTARCLTEFMGTFCAISFGDGVVANALLSSTGGNGMGAGWIAFGFGM
AFTFPIMMFGYGSSYFNPAMALAGL
MRNEISVRDYFALAASQMVAGFCAGIFVYLLYLPHFQKIPEVSMSLEDALPSLAPAN

KVTLQNLLGNSSKVGDASAKSSE
LAEAHTIIAADQGKKLGVLCTRPAIFMPIHNFFVELMGTATIVVGANLIGSRMSMITSS
ADMQALGIALQPFLVGMLIMT
LILAMGGPTGFAANPARDMGPRLAHMLLPIPGKGTSEFSYGIMINIGMLLGGVLGGA
LIMAMDQVRFWTL*
>jgi|Chytri1|1197400
MPYTGNYSLATRCLTEFVATFFAIGFGEGIIANAELPSTKGHGMGFAWISFGFGMAF
TFAIMMFGFASAHLNPAMVLSLW
IRNELSAGEFFALAASQMAGGFCAAIFVYLVYLPHFSTVPEPSGSQEDHLLRTRDYI
DPSALRFASYSTRKVVASPAGRV
SSQRKTFSQRLGEAKYYLLAQNFDDAPETVIEHLIGGTFALHGAEVPFPTGIETVND
AADAETQPVQLGALKRRHSLQVA
DMQRLLRKMEKELSTSENVGAPLNHTSTYGIDSATPQRASVLDLAAPAAALRRSPA
FLTDIHEIRASDTTVGVAPANSNT
DNKVVFQNSVQSMSNMSVTAKLPKEEALDRAAIAADQATKLSVFATRPAIFLPIHNFF
VEMIGTAVLIFGASLIDSRMSM
ITNTPELQALGIALKPFLVGVFIMVLVLALGGPTGFAANPARDMAPRLAHLLLPIPGKG
TSELWYGLVVNIGMLAGGALG
GVLVIAVDQIRTWTI*
>jgi|Chytri1|163686
MPYTGSYSLATRCLTEFVATFCAIGFGEGIIANAELPSTKGHGMGFAWIAFGFGMAF
TFAIMMFGFASAHLNPAMVLSLW
IRNELSAGEFFALAASQMAGGFCAAIFVYLVYLPHFSTVPEPSGSQEDHLLRTRDYI
DPSALRFASYSTRKVVASPAGRV
SSQRKTFSQRLGEAKYYLLAQNFDDAPETVIEHLIGGTFALHGAEVPFPTGAETVND
TADAETQPVNLIPGTSALKRRHS
LQVADMQRLLRKMEKELSTSENATPQRASVLDLAAPAAALRRSPAFLTDIHEIRASD
TTVGVAPANSNTDNKVVFQNSVQ
SMSNMSVTAKLPKEEALDRAAIAADQATKLSVFATRPAIFLPIHNFFVEMIGTAVLIFG
ASLIDSRMSMITNTPELQALG
IALKPFLVGVFIMVLVLALGGPTGFAANPARDMAPRLAHLILPIPGKGPSELWYGAVIN
IGMIAGGALGGVLVIAVDQIR
TWTI*
>jgi|Clarep1_1|745788
MDLPSMETVASPSAHLPYERIASGCLTPMSESPTEVDCICQRHPNSPPLKCAFPPG
LSPVDYNDSPKQQPSSPQRPTWLL
SKLLSLNALTSRRNPLIRAFCGELVGTFLMLWWGYSAVYTAVIGDAMKGLWQVAST
WGFAIAVSIYVSAPFSGAHLNPAF
SIANALMYADFGWTKCAVYIVAQFMGSSAAAFLNLALWGPAIRRFEAANNIVRGSPE
SIRSAMIFGEYFPNPDIFKPHQG
ESLADTSTLITPMTAFFAEALGTAIIGIVSTALGDTQNTSIARGFEPFMLGFAIACNIVAI
APLTQAGFNPARDFSPRLA
AWAGGWGTDIAFSYWWVYVFGPIVGAVAGVAVYECFFRKVDQDASTADGDERGA
R*
>jgi|Clarep1_1|850245
MSDFSEIPDQDYSAIDVDNGGASDRDTLAPIIVAAPPPPKIDDDFNDKITTLSWNPAT
WIQDIKEEIRDFSWQMNEKRRV
LIRAIAGEGLVTFLFLFIVEAVQVNNGRQENPESLVLSAVSTAFASVALIYSFADVSGA
HFNPAVTFATIVTGKVSIRKG
LAFIGVQLFSAILATLFLMAVFPKPKDTGYLSIPASVVVSLDASAKKVNAFFMELILTFIL

VYVIFATAFDTVDTSNAAP
VSSPSDATKPHSGEHSSGEPSQPAPKVSKDVGRYLTIYTTSGNTKAGFAPLSIGFTL
GFLCFLGGTVSGGAFNPARVFGP
ALLTGNWDNHWLYWLADLTGAALAGWTQSLFAHKAVQSSGSIKTNKHT*
>jgilClapol1_1l773898
MTRTVATSLLLQSLAEFYGTFLFLFLSLAGVQSAIFQGPAPTFVLQIALVFGLALATAV
FLTFRISGGALNPAVNLGLLV
AGKMDPVKAAAYTVAQSAGAVAACAAVAAAFPGSHGKAFFGANAVQIDPATGAPVC
TVAQAFAIEAVLTAGLVLVVLFLA
VDKQRVTFLAPLLIGLYVFIAHLIAIPYTNTSINPARSLGASVVAGVWSDHWIFWAAPL
AGGAVAGLVHRFFKAVDYEKL
NQGQDADSDGPSLA*
>jgilClapol1_1l844221
MYKHATSSYTNNSSNGGSSHSTTAAATPDPASSPIPSTSDRVPLLGRRPQFIPPKHS
ELTNELVQAAGEFVGTLFFLFIL
FAAIQASLLHEEHFLLSISLSAGFALTSAVWLTYRISGGALNPAVILALLLVQKISFRKA
GLYLIGQLLGSTTAAFFVSF
MAPDAWGREFQGANKVAEGTSLLQAFCLEAVLTAGLVLVVLFVAVEKSKATFLAPLVI
GLYVIAAHLVAIPYTNASLNPA
RTFGSALISGNWSDHWLFWLAPLSGASIAAGLYKAFKLFNFEVLNPHQEDDHEPLG
YI*
>jgilRhihy1l194264
MDQARKYLAEFLGTFIMVFFAVTANSSGPIAGALVGLGQVAAVNGLGVTLAIYTTADI
SGAHLNPAVTLVFAVWHSFPWS
NVAGYFVAQFAGAICAAGLNYGLYSSTIARFEAMSGIVRGAAGIGKECIDLWTVLSKS
RCFPIPVESSPEHIASLQSFVS
PGLAFLTEMVGTAILLFVILSLIDKKNTSISPHIVPVLIGCTVTSLISVLAPITQAGFNPV
RDFAPRLVALAVGWGQIAI
PGPRNGFWVYILGPIVGAQLGAALHYVLYKATSVSLRYPP*
>jgilRhihy1l497641
MFIFIFLALGGVQSALKTNAADLAVNPTLAFIEISMCFGFGLAAAIFFSYRISGGALNPA
VNFGLFVAGVMDPLTLIAYT
ISQMIGATAACGVVSVVFPGDFKGANQVFAGTSITQAFFLELILTAGLVLTVLFLAVEK
SKVAFWAPMMIGIYVFIAHLL
AIPYTNTSINPARSIGASIVSGIWKDHWIFWVAPLAGGAIAGGIYRGYKLVEYETLNPG
QDADHSPVDTSRV*
>jgilNeocon1l775345
MKQYEYGEDSKSNIIKDCISAIGEFVGTTFFVFMGISSVNSAFHSMQTKNLDNIAYIQ
DVTNSTVVNRGEATGADPASQI
QIAFGFGIGLMCMVWAFYRISGGHLNPAVTLGLFCSGAVPPVKAVLYIIGQLCGGML
GALFAKILFPKPYDGITRGANGI
GDDKSVTAGFFLELILTFLLVFVVHMLALEVNNARNVAPFVIGAIVFVGHLIAIPITGTS
MNPARSFGASVVANQWDDHW
VFWIAPLCGGILASICYKIFSFFNIQALNGNQDHTTAMVYDNKAIPVNSVESLPQ*
>jgilNeocon1l798305
MKQYEYSGEDSKSDIIRDCISALGEFVGTTFFVFMGISSVNSAFHSMQTENVDNIAF
LKDVTNSTVINPGYATGASPASQ
LQIAFGFGVGLMCMVWAFYRISGGHLNPAVTLGLFCSGAVPIVKAVLYIIAQICGGML
GALFAKILFPKPHDGVTRGANG
IPDGRSVTAGFFLELILTFLLVFVVHMLALEVNNARNVAPFVIGAIVFVGHLIAIPITGTS

MNPARSFGASVVANQWDDH
WVFWIAPLVGGILASICYRIFNYFNIKALNGSQDHTTAMEYGNNPMAINSTESLPH*
>jgilRhihy1l712852
MSDFLVSKETVGETSVSYSTQAVGIEVQKVSAVETLDLTQLLIKCITEMLGMFIFIFLS
LGGVQSALKTNANELSVNPTL
AFIEIAMCFGFGLSAAIFFSYRISGGALNPAVNFGLFVAGVMDPITLAAYTIAQMAGAS
IACGIVSVVFPGDFKGANQIF
AGTSVAQAFFLELILTAGLVLIVLFLAVEKSKITFYAPMVIGTYVFIAHLLAIPYTNTSINP
ARSFGASIASGIWNDHWL
FWVAPLLGASLSGALYRFYKFAEYHTLNPGQDADH*
>jgilNeolan1l1680131
MKQYEYGEDSKSNIIKDCISAIGEFVGTTFFVFMGISSVNSAFHSMQTKNLDNIAYIQ
DVTNSTVVNRGEATGADPASQI
QIAFGFGIGLMCMVWAFYRISGGHLNPAVTLGLFCSGAVPPVKAVLYIIGQLCGGML
GALFAKILFPKPYDGITRGANGI
GDDKSVTAGFFLELILTFLLVFVVHMLALEVNNARNVAPFVIGAIVFVGHLIAIPITGTS
MNPARSFGASVVANQWDDHW
VFWIAPLCGGILASICYKIFSFFNIQALNGNQDHTTAMVYDNKAIPVNSVESLPQ*
>jgilNeoGfMa1l392362
MRQYEYGEDSKSDIIRDCISAIGEFVGTTFFVFMGISSVNSAFHSMQTRNVDNIEFL
RDFTNGTVVNPGHATGADPASQI
QIAFGFGVGLMCMVWAFYRISGGHLNPAVTLGLFCSGAVPIVKAVLYIIAQICGGMLG
ALFAKILFPKPHDGITRGANGI
AGDKSVTAGFFLELILTFLLVFVVHMLALEVNNARNVAPFVIGAIVFVGHLIAIPITGTS
MNPARSFGASVVANQWDDHW
VFWVAPLLGGILASVCYRFFNYFNIRALNGSQDHTTAMEYENKAMAINSTESLPH*
>jgilNeoGfMa1l569445
MKKYEYGEDSGSNTIKECISALGEFVGTTFFVFMGISSVNSAFHSMQTKNLDNISLL
RDFTNGTVINNGYASGADPASQI
QIAFGFGIGLMCMVWAFYRISGGHLNPAVTFGLFCSGAVPIVKAVLYIIAQICGGILGA
LFAKILFPKPHDGVTRGANGI
PDGRSVTAGFFLELILTFLLVLVVHMLALEVNNARNVAPFVIGAIVFVGHLIAIPITGTS
MNPARSFGASVVANKWDDHW
VFWVAPLAGGLLASICYRIFSFFNIQALNGNQDHTTAMEYDNKAIAVNSVESLPH*
>jgilNeoWl3_1l563152
MKKYEYGEDSGSNTIKECISALGEFVGTTFFVFMGISSVNSAFHSMQTKNLDNISLL
RDFTNGTVINNGYASGADPASQI
QIAFGFGIGLMCMVWAFYRISGGHLNPAVTFGLFCSGAVPIVKAVLYIIAQICGGILGA
LFAKILFPKPHDGVTRGANGI
PDGRSVTAGFFLELILTFLLVLVVHMLALEVNNARNVAPFVIGAIVFVGHLIAIPITGTS
MNPARSFGASVVANKWDDHW
VFWVAPLAGGLLASICYRIFSFFNIQALNGNQDHTTAMEYDNKAIAVNSVESLPH*
>jgilNeoWl3_1l908511
MRQYEYGEDSKSDIIRDCISAIGEFVGTTFFVFMGISSVNSAFHSMQTRNVDNIEFL
RDFTNGTVVNPGHATGADPASQI
QIAFGFGVGLMCMVWAFYRISGGHLNPAVTLGLFCSGAVPIVKAVLYIIAQICGGMLG
ALFAKILFPKPHDGITRGANGI
AGDKSVTAGFFLELILTFLLVFVVHMLALEVNNARNVAPFVIGAIVFVGHLIAIPITGTS
MNPARSFGASVVANQWDDHW
VFWVAPLLGGILASVCYRFFNYFNIRALNGSQDHTTAMEYENKAMAINSTESLPH*

>jgilSpipu1l7981
MTELQKISSPTTDYAVDIERRGLTSVAPDDDEIAPFDDLNDAPGKFSWNPLEWIKEF
QQDWREFSWAMTEKRRVLLRAVF
GEGLCTFLFLFIVEAVAVNNGRQEQPENLTLGAVATAFCSIALIYSFADVSGAHFNPAV
TFATMVTGKVSLRKGLAFISI
QLFASIFSVVALLIVFPGPHPGYSSIPASVVVDIDSSAHLAQAFFMEVILTFILVYVIFAT
AFDTVDTSNEVKVKAKGAG
GSAAAQADDKSVGRYLTIYTTSGTTKAGFAPFAIGFTLGFLGLIGGSVSGGAFNPAR
AFGPALLSGHWNNHWVYWLGDFI
GAALAGWAQHFFAHEAVQHSGASAKTKKGAENERVEEAQKAARAGDLS*
>jgilBatsa1l792
MPYSGPYSLATRCLTEFLGTTLAIFLGTSVIANELLPLTKGHSMGFGWVATSFGMSF
GVAIMMFGFASAHLNPSMTLALF
IIGRLSFTDFLALSCAQLAGGFVGAVAMFLVYMPHFRTVPEPQPDQTQSALSTHLLR
TRDAIDPSALRFASYNTKSSASA
PPKNMADVLRDLRYYLTTQTGDTQQVFDLLSLGTLDLAGVEVAFPSDSTLSVPNTF
NNGIDPIKPLKRRHSIQVAEMQRR
LRHLEAAMTTNPHVRPHNTSSTHTTLFKAESVELDELTGSHTFHQSRSSAVKPHPP
SMVAVHTRIDTDTMAGNSSPTLPQ
PDIIDQRNPLNHNSHKKRSTAALEVVSPPTLTHMETVHRASTIAHQAAKLSAFCNRP
AIYLPLHNILVEVIGTIMLVLGA
LLLDQRFSSTTTAQGAADLVSIRTTELLFHNALAPFLIAVYIQVCILALGGPTGFTANPA
RDLGPRLAHWLLPVPGKGGS
EWNFCLVVMTGTLLGGCLSGVLFLGLGNITM*
>jgilChylag1l277068
MGYTGPYTLPSRMLTEFLGTFLAIFLGLAIVANELLPSTKGHGMGFGWIAFGFGMAF
TVAIQIFSYASAHINPAACIALW
VRGDIDAVDFFALSAAEMAGGFAAACMVYLLFLPHFKTVPETAALSPEDRLLRTRDD
IAPSALRYASYNTKSSPSGAATL
NQRLNEAKYYLSNEAFDDAPEKVMEHLVGKTYMLHGIEVPFPKEDDEEQGAKPER
KIKRRHSLQVADMQRRLRMAERELS
FGPSDDIHLRPTSATTIDAETHTDAPVEPTTSVAAPSKSRAVDTLRRTLNRQASKSSI
VTFEAREAALARAATAADQATK
LSVFATRPAIYLPIHNFFVEMLGTAMLIYGAFLIDDHLRGAAAEAGVLVSPGTAGTLKL
VLDASFSPLLKGFYIMVLVLG
WVDQLVLLQTLQEISHLDLHITCSRFLGREVQSGTMG*
>jgilCaecom1l448829
MKQYEYSDDSKSSLIKDVISALGEFVGTTFFVFLGISSVNSAYESMYARNAQEIQNIA
AVSNATIAHNGDAPKSGLDPAS
QIQVAFGFGFGLMCMVWAFFRISGGHLNPAVTLGLFCSGAVPPIKAVLYIIGQCCGA
MLGALFSKAIYPTPTEGPFRGAN
GISDDKSRTAGFFLELILTFLLVFVVHMLALEVNNARNVAPFVIGTIVFVGHLVAIPITG
TSMNPARSFGASVVTGEWDD
HWIFWVAPLCGGILASVFYRIFSFFNIQALNGNQDHTTAMDENRAIAINSTESLPH*
>jgilNeosp1l702287
MKQYEYGEDSKSNIIKDCISAIGEFVGTTFFVFMGISSVNSAFHSMQTKNLDNIAYIQ
DVTNSTVVNRGEATGADPASQI
QIAFGFGIGLMCMVWAFYRISGGHLNPAVTLGLFCSGAVPPVKAVLYIIGQLCGGML
GALFAKILFPKPYDGITRGANGI
GDDKSVTAGFFLELILTFLLVFVVHMLALEVNNARNVAPFVIGAIVFVGHLIAIPITGTS

MNPARSFGASVVANQWDDHW
VFWIAPLCGGILASICYKIFSFFNIQALNGNQDHTTAMVYDNKAIPVNSVESLPQ*
>jgi|Neosp1|708696
MKQYEYSGEDSKSDIIRDCISALGEFVGTTFFVFMGISSVNSAFHSMQTENVDNIAF
LKDVTNSTVINPGYATGASPASQ
LQIAFGFGVGLMCMVWAFYRISGGHLNPAVTLGLFCSGAVPIVKAVLYIIAQICGGML
GALFAKILFPKPHDGVTRGANG
IPDGRSVTAGFFLELILTFLLVFVVHMLALEVNNARNVAPFVIGAIVFVGHLIAIPITGTS
MNPARSFGASVVANQWDDH
WVFWIAPLVGGILASICYRIFNYFNIKALNGSQDHTTAMEYGNNPMAINSTESLPH*

>jgi|Hompol1|2763
MVYSLGTRCTTEFLATALAIFLGDSVIANELLPGTKGHAMGFGFVAIGFGASFGVAIM
MFGYASSHMNPSMALALWVIGR
LNSSEFFSIAACEIAGGFIGALAMYLTYLPHFRTIPEPHIDDDGVHHLLRSKDTIDPAA
LRIASYNTRSSPSHPRGNFKD
RLREARYYLTAQDGDTDQVLKILSIGTLDLAGVEVPFPSNDVEQPMLDVLILGALLLD
DRFSQIKNALASVDASTSSGTG
GQIAQLVFDNGLTPFFVSLFIQLCVLALGGPTGFTANPARDLGPRFAHWILPISGKGH
SEWNYCAVVFAGNILGGIIGGS
LFLAIQKVHGGI*
>jgi|Gorhay1|225452
MQKQSPLTEDIKTALAEFLGTLFFIFLSLTCVQVTVGGTHDLFTNMTTNDQMLTFFLH
QKSILLSIAAAFGFGLAVCIAF
TAPISGGHLNPAVTISFLALGEIKPLRALFYIVAQCLGATAGAAFARLVSGYSVLYGVN
APATGISEISAVCCEALLTFV
LVYTVLTTAVDATVSKGLAPLYIGLSVFVIHISSVFIDGTSVNPARSFGASLVAGKWDS
HWVFWVGPVVGGLVASGFWKL
FKSI*
>jgi|Batde5|36652
MPYSGKYSLATRCLTEFLGTALAIFLGDSVIANELLPLTKGHSMGFGWIATAFGMAFT
LSILMFGYASAHVNPAMTLTLF
IINKLSFVDCICLILSQLAGGFMGAVAMFIVYLPHFRTIPEPPASTACGSDTNANVLLR
TRDAIDVDALRVASYNTKSSA
SASKSFSDILKEAKYYLTSQSSDSKHVFNLLSLGTLDLAGVEVAFPDDPLKASSTTDL
ETPQNQHRPLQRRHSIQVSEMQ
RRLRRLEASMAPSASSTALTRTQTIFKSDSASVHTAHSSVVSPCTHSEDNLASKPAV
IVCSSATPTDSLANVKDARFAQN
SARFNAILANKSSSDSNLNPTSSNSHPTNKRDRHLDFQVLGDLKTTHAEALHKAAIR
AHQAAKLSAFCNRPAIYLPIHNF
TVEVIGTTMLCLGALLLELRFSMAATAIIATNTIPAETVELVFHNGLAPFFIGIFIQTCIFA
LGGPTGFTANPARDIGPR
FAHWILPVPGKGASEWNFCGVVTLGTFSGGVVAAVLYMGLSTIP*
>jgi|Batsa1|6980
MTPGITHDIEIVTDPTNRTSYNRRLSKTCGSPSDYGHKICRASVGTAEMEDIEEDKP
KTQSNRSKLEGSLRSFYRRHPVL
VYHIRTFAAELVGTFLLVAVGTGAVASAVLTGALKGLWQVAVVWGCGIAMSIYMVGHI
SGAHLNPAVSFVMAVFGHHTGF
KWTSLIVYVVAQVLGSFIAGTVQLAIWEPFILLFEIRQGIIRGEQPGCMRSGMIFGEYF
PNPDMFIQTSPNPGGGVTAFG

YDLVSVSRAMTVEAFGTGVIVVMLFTLTDRHNKGAHPAMVAPAIGATVAALTAMFAP
VTQAGWNPARDFGPRLAAALGGW
GMCAFGRDGTFWIYSVGPCIGALVGGMIYFGLLCRADEEVDAISGKSANKDL*
>jgilBatsa1l10237
MAVFTSKTSFPWARVPAYITAQVLGAALAGCVNLVIWDPLIAQFETHHSIIRNQLPGC
SHSAMLFGMSFPNPDIYLPTLP
DSASLVSPWRALSVEIIATAIHTLIVFGLCDRSNRHSVAPCVMPAAVGAMTAALISVFA
PISMAAMNPARDFGPRIAAAL
GGWGGCAFGSNGSFLVYIFGPCLGAIIGAAVYFYVLCPSDDELKEHED*
>jgilBatsa1l10428
MSQTTFNAPEATLDPLQSECPDKFHNVNVISPSTIKSDHGSNIVLVNVPKEQFSNQK
TSAKLISTRAIYQSLQSQHPSFV
LQLRKFFAEFLGTFLLVSIGIGSVASAVLTGSLKGLWQVAVVWGCGVTVSIYTVGHIS
GAHLNPAVSLVMAVFGHYTGFK
WSSLVLYITAQLAGSIVAGAVQLALWSPFITLFEQRNGIVRSADPGCMRSGMIFGSYF
PNPDMFPLNAADASKGIVSSGL
GLVSVPRALMTEALGTCILLLVIFALTDRRNKNKSIHEALVPPTIGATVAVLISMLSPITQ
AAFNPARDLGPRIIASLAG
WELCAFGPSGSFWIYIVGPCLGALLGGVTYFGLLYQSDEEIAYATEQNTPLAE*
>jgilPirE2_1l20241
MKQYEYGEDGKSSIIKDCVSALGEFLGTTFFVFMGISSVNSAFESMQAQNFDTINF
MRDVTNSTVVNPGIATGATPASQI
QIAFGFGFGLMCMVWAFYRISGGHLNPAVTLGLFCSGAVPPLKAILYIIAQVCGGML
GALFAKVLFPTPHDGVTRGANGI
PDGRSVTAGFFLELILTFLLVFVVHMLALELNNARNVAPFVIGAIVFVGHLIAIPITGTS
MNPARSFGASVVAGEWDDHW
VFWVAPLVGGVLASICWRIFNFLNIPALNGSQDHTTAMEYENKAIAINSAESLPH*
>jgilHyacur1l613526
MASFASAAPLAHHDRAEPHHHHHERNEATTAPAAEPLDAEPLDAGPREWRRFLLP
YAGEFLGTLTLTLFGIMAVSAGVLT
GSLVGTGQVAIVWGIGVALSIYTTASVSGAHLNPAVTFMFALFKRYSGFPWARVPGY
FLAQFLGAFVAGLLNWAMYYPYI
DLFDATNGIVKGTPESALSAMVLPQYFPNQAVYTGLRSSAPASFAFPTQAQLIGPGQ
AFFAEALGTAILCYVIVALTDRC
NTSLSQQFAPMLIGLTVTALICTLAPISQAGFNPARDFGTRVVAYIAGWGAAAIPGPQ
DAWWVYVVAPFVGAPVGAGVFF
FILRKPDEYCELKDKCE*
>jgilObemuc1l1007196
MEKRKSIVDGTKMRQYLAEFVGTFLLVFFGTASVSSAAISGALVGLGQVATVWGCG
VALSIYCVGHISGAHLNPAVTLAF
AVWDSFPWSNVIGYVVAQFLGSLCAAGLNWGLYSTSVARFEEANGIIRGAPGSEKS
ALIFGEYFPNPGTYTIPKGSTAAE
IISIQSFISVGLAFTAELVGTALLMFIILALIDPKNSSVSPKAVPALIGMTVATLISVLAPIS
QAGFNPARDFAPRIVAL
ACGWGSKVALQDGFWVFILGPIVGAQVGAALHFGLYKADFERSSKVGVAREETES
SAAAAEAGRV*
>jgilClarep1_1l380373
MNDKTLLDMNSEIDRAAASEVSYDRLDGPTPSPDTDDVRPSSRPRRQRPRTHWAL
SMITRRLAFFVTKRNPLFRAFFGEV
IGTFLLVWWGLSSVSTAVIGGALSGLWQLASTWGFGVAIAIYIAAPFSGAHLNPAFSI

ANAVLYPDFGWMRCGVYIVAQF
IGALAAGFLNLALWNPAIVRFEASNNIVRGTPESIRSAMIFGEYFPNPEVFKPHEGEL
FSQTRALMSPVGAVFIEALGTA
IIGFVVTALSDSPSSIVRGFEPFMIEPQLSGSLSPLLATPQAPLSAASNPS*
>jgi|Rhihy1|716491
MKTALRTYFAEFVGTFIYVFFALLQNSAGSIAGGLVGSDGAVIDGLGVTLAIYCTGYIS
GAHLNPAVTIALAAWASFPWS
NVVGYIAAQFIGALCAAGLNYGLYSSTVSRYEELNGIVRGSPGSERTAMIFGEYFPN
PASYIIPAGASPEILATLQAHVT
PGLAFASECVGTAILMLIILCLIDKKNASISPPLIPLFIGATVFAIVAVLAPISQAGLNPTR
DFAPRLVALAAGWGKIAI
PGPQNGFWVYIVGPIVGAQVGAAFHYVLFKFGVNDEFALPSGRVEDEHHELKSV*
>jgi|Pecora1|6357
MKQYEYSDDSKSDIIRDFISALGEFVGTTFFVFLGISSVNSAFQSMQARNKDNIEFM
QDVTNSTVVNPGYATGADPASQI
QIAFGFGFGLMCMVWAFYRISGGHLNPAVTLGLFCSGAVPPVKAVLYIIAQVLGGML
GAFFAKVHYPKPYDGITRGANAL
PDGRFASGFFIEIILTFLLVFVVHMLALEVNNARNVAPFVIGTIVFVGHLVAIPVTGTSM
NPARSFGANVVTGQWDDWWV
FWIAPCIGGILASVCYRIFSFFNIQALNGNQDHTTAMEYENKAIAINSTESLPH*
>jgi|Batde5|92869
MRVKSEPEIVAVSQNIDDSVETPRQSVGIETVIISQGVECEFQSLASLQLNKRLALAS
RRRFVAELIGTFLMLTFATGAV
SSQVLTGALKGIWQNAVVVGIATTCAIYLSGSTSGGHLNPAITLAMATFGCKTGFQW
NRVPSYILAQILGGILAGIANLF
IWNPIIVQFESRNNITRNQLPGCTHSAMLFGTYFPNPDIYKPGSPDAINLVSPWRAF
GVEVFATAILAFVIVGLCDRGNR
PTIHPAIIPPAIGAIVAALISVFGPITMAAMNPARDLGPRIAAAIGGWGECAFGGDMSF
LIYTFGPCIGSILGAAVYLTF
LYHSDEELKDRNHKSTAIMTTTSNQLPLASDLPEKLDSETLVEHSAEVVSLEVVTEH
LGFAPVHFIDDMIDRVNSLLYKS
MAKLEELVSVELGKGIETDRGMASIETLFESCVDKRFDRFEVFGLRNVFAVQPDLA
MQLTAFQECNVDITLEQELALDAD
IDVLRKQLLAGKLQEKDKYLDNVISHLNIFGDQLVKLESIAVDNKIYPLSEKLVELSDKI
ATLSSVTDQVHVRTTHPIMR
EKICDSDRRMQLLQSEIAMHIKKRRHIAEVQEGLAMGKGNKANGSFSNMSAWDVT
REYRDAMSIGSVKEMEAFKENVLL*
>jgi|Pirfi3|411999
MKAYEYGEDGKSNIIKDFISALGEFVGTTFFVFMGISSVNSAFESMKTQNYDTINFLR
DVTNSTVVNPGIPTGATPASQI
QIAFGFGFGLMCMVWAFYRISGGHLNPAVTLGLFCSGAVPPVKAVLYIIGQVCGGML
GALFAKALFPTPHDGITRGANGI
PDGRSVAAGFFLELILTFLLVFVVHMLALEVNNARNVAPFVIGTIVFVGHLIAIPITGTS
MNPARSFGASVVAGQWDDHW
VFWVAPLLGGVCASIVWRIFSFFNIPALNGSQDHTTAMEYENKAIAINSTESLPH*
>jgi|Blyhe1|24102
MYKPFVAEFLGTFMLVMFGIGSVSAVVLIGALTGLWEIAIVWGFGISLAIYATASVSGA
HLNPALSLTMAIWHNRTGFTF
TRAAGYIIAQVLGGIAAGCVNLSIYEPFITDYESRTGIIRGEAGSERSAMDGALAGTLL
GPTHALFVEAFGAGVLAFMAF

ALTDPGNRAIPRKELAPCFIGLTAASLIALLSPITQASFNPARDLGPRIVAALAGWGSI
AMPGARSGFWVYIVGPCIGAP
IGATVYLLLLEKRREMPSLEDGEPTR*
>jgilPiromy_p0_1l547337
MKAYDYGEDGKSSIIKDFISALGEFLGTTFFVFMGISSVNSAFHAMQSQNYDMINFM
RDVTNSTVVNPGIATGATPASQI
QIAFGFGFGLMCMVWAFYRISGGHLNPAVTLGLFCSGAVPPLKAVLYIIAQVCGGML
GALFAKILFPKPHDGVTRGANGI
PDGRSVAAGFFLELILTFLLVFVVHMLALELNNARNVAPFVIGSIVFVGHLIAIPITGTS
MNPARSFGASVVSGQWDDHW
VFWVAPLLGGILASVCWRVFSFFNIPALNGSQDHTTAMEYENKAIAINSTESLPH*

>jgilMicG_I_3l4284
MAGEVLKGVQSAFGEMFASFVFGFAVYSALLGSALSLQSAASVVIALTVGFSGVGVI
YSFCDVTVAHFNPAITLTAILTG
KLGIIRGLGYILAQYIGFILAVCALLPCSPLEYKATLDVIRPKPADFGGDNLNIFWSEFF
FTAILVHIAFAVGVNPYKPK
VDVDGNFVNPEEDEPVDRRVTAPLCIGLTLGFLAFLGLATSGGVFNPALLFAPVIMS
NTWTKFWIYCTAEYSGGLIGGLL
QVFVLYKISY*
>jgilEncro1l1331
MARETLKTLQSMFGEMVASFVFGFAVYSAVLGSTVSQQSAARVIVGLTVGFSAIGIIY
SFGDVTIAHFNPAITLTAILTG
KIGIFHGLGYILAQYVGFMLAVCALIPCSPIGYKETLNIIRPAPSSFGGDNLNVFFTEFF
LTAIFVHIVFAVAVNPYKPK
VDTDGKFVDPDEKEPVDRRITAPLSIGLTLGFLAFLGLASSGGAFNPGLTFAPVIMSN
TWTHFWLYFGGQYLGGFVGGLL
QVFVLYKLSSN*
>jgilEncin1l1333
MAKEALKTLQSMFGEMVASFVFGFAVYSAILGSSISQSSADKVIVGLTVGFSGIGVIY
SFCDVTIAHFNPAITLAAILTS
KIDVLQGLGYMLAQYIGFMLAVCALLVCSPVEYKETLDTIRPGPTDFGATSLNVFFAE
FFLTAIFVHIVFATAVNPYKPK
VDTEGKFVDPDEKEPVDRRITAPLCIGLTLGFLAFMGLASSGGAFNPGLTFAPMAMS
NTWSHFWIYLGGQYLGGLTGGLL
QVLVLYKLSSD*
>jgilEnccu1l1465
MTRETLKTLQSTFGEMVASFVFGFAVYSALLGSALTEQSAARVIVGLTVGFSGICVIY
SFCDVTVAHFNPAITLAAILTC
KLGVLRGIGYIVAQYIGFILAVCALLPCSPVGYKETLNIIRPTPSPFGGDNLNVFFTEF
FLTAILVHVAFATAVNPYKPK
TDTEGKFVDPDEEEPVDRRITAPLCIGLTLGFLAFLGLASSGGAFNPGLTLAPVIMSN
TWNHFWAYFAGQYLGGFVGGLL
QVLVLYKLSF*
>jgilEntca1l1960
MNYRNYECFVAEFMASLIFSIVANFAIVTNVANAAVAIALAKMSIGYSFEHLTLRHVNP
AVTVACALIGLLHFKSAFVFI
IFQIIGFICGSGITRLLFGKEYLRVYETTQPTSSVRIIALEFGVSFLLALVVIENLVYAKG
LIQYNRMGTTKSYRRNKHA
LPFSIGAITGTGSFISSRAEGGSFNPAFIFATFLVTNKYSFFWEYMVGDFLGSAAGAL

VVRHLLH*
>jgilEntca1l1963
MNIRTKLTCQKLFAEFLCSLIFGFAVYSAVLNTKASENPAPSTAVGLTVAFSSIALIYTF
CDHCASHFNPAITIAALVTG
KLDLALGIGYVIAQLLGFFIASLLAVLCFPYGYSKTLDLITPGAXVYSAVLNTKASENP
APSTAVGLTVAFSSIALIYTF
CDHCASHFNPAITIAALVTGKLDLALGIGYVIAQLLGFFIASLLAVLCFPYGYSKTLDLI
TPGAVSDEISDHNIFWAEFI
LSFILVFVAFEVGINAVREPGVTLFVGETQIDRSKFAPLTIGSTLGFLAFLASSTSGGA
FNPGIVFGPSIAGGNFEYFWQ
FVVAELCGGLLGGLVQVFLLFK*
>jgilNemdi1l47
MALTFNWTIGRSLLAEGLCTALFAYAVYSVVTGTAISDSLATQNVAVGLTIAFSSVAIIY
AFMDVTIAHFNPAITLAAIL
LGKLPIWMGLGYIIMQCAGAMLASAAMLLCKPGSSQSLLALTRSTVAPDATIGNAILT
EMILTGVLTYVAFSVAINVFNP
PTQIKIEVPDEVEDNDKLRVVTSQAPNRSAFAPIAIGFTLGFLSLLGGGSSGGVFNPA
ITLPPVLFSGVWYNVWVYWVGQ
FVGGFVGAFLHVVIFAKSI*
>jgilNempa1l578
MGLTFNWIVLQSFLAEGLCTALFAFAVYSVVTGVNITPCADGVANIAVTLTIALTSVAIIY
AFMDITIAHFNPAITLAAI
VTGKLPIFMGLGYIIMQACGAMLGSAVMLLTKPGSSSELLGYTRSTLGPNASIGNAIL
TEIILTGILTYVAFSVAINVFN
PPTQVKIEVPDEVEDNDKLRVVTSQAPNRSAFAPIAIGFTLGFLSLLGIGSSGGVFNP
AITLPGTLFSGVWRDVWVYWVG
QFVGGIGGALLHVWIFAKTV*
>jgilEdhae1l3100
MGLTFNWKQVQSYIGEMAASFVFGFSVYSAAISSSLTDSMCGPVIVGLAVCFSSIAII
YTFADITLAHFNPAITFSAIIF
GKLIWYKGAIYIISQCLGFMIAAAVVLGCYPSTVRNKLEIIRPKKVDDDVTKGNLICTE
MFLTGILTFVAFQVAINVYKK
PKYIKSEEEKLLPEGAEDHIDIEDSKPDTTILAPLVIGLTLGFLAFLGFSSSGGVFNPGL
VWAPVLFSGDWYDSWAYWVG
EFTGSLVGAAIQVFILARMY*
>jgilAntlo1l1554
MGTFETYQKYLLPYLAEMACSFVFGFIVYAATISQAQTLSAAGQVIIGTAIGFSSVALI
YTFCDMTLAHFNPAITFSAMV
FGHIPVIRGLTFICAQLCGFMIASVVVLGCFSGSSYTLLNIIRPKRAFDEVNAGNIICNE
AVLTGILVFVVFSVAINTFH
EPDLDEETMKMKVCDKRTEKK*
>jgilEntbi1l2245
MNTSTKLICQKLFAEMLCSCIFGFAVYSAILNTKASNSSISSTTVGLTVCFSSISLIYTF
CDHSVAHFNPAITIAAICTG
KLDILLGIGYVIAQLIGFILATLLTVVCFPYGYLKTMEFIASARISDDISTVNLFFTEFILS
FILVFIAFEVGINAIREP
GVTLFVGIKQIDRSKFAPLTIGITLGFLAFLASTTSGGAFNPGIVWGPAIMGGNFDDFV
IYIISELSGGLLGAFIQVFLL
FK*
>jgilNosbo1l830

MVSRNILKTQAILGEMFAAFVFGFAVYSALVGTSQTENTSASIIIGVTLGFAGVAVIYSF
CDVTIAHFNPALTLTALLTG
KIEIIMGFFYILAQFIGFILAALAVVACFPGAYRDKLDIMRPKFVYTDTRDGTVFASELF
LTAILVYVAFAVGINPYQSP
KDEEGAPLDPDEEIAFGRKITAPIAIGFTLGFLGLCSLSSSGGAFNPALVFAPCLLNG
RWTHSWVYLLAEFAGGIIGGLL
QSTIFYKGTHTIKDIDKSFTAIVGPNGSGKSNVIDSILFVLGFRAKKMRHQQVTDLIYS
DGQRESEAFVELIFNKFRVKR
SINLNKSTKYFLDNSETTSSNIIKIMMEEGVDMEFNRFLILQGEIESIAMMKPKESSSK
DGLLEFLENIIGTSSYKKEIE
DVQNEIKNLEQLKTDAQTSLNFIKKDYDHAEILKNQNEKAVKEKIDFLIKNNELFNLKN
ILLTQKKIEAEDLKNKFQMNR
KFKINK
>jgilOrdco1l1532
MAGEVLKGFQSALGEMFASFVFGFAVYSALLGSALSLQSAASVVIALTVGFSGVGVI
YSFCDVTVAHFNPAITLTAILTG
KLTILRGLGYILAQYIGFILAVCALLPCSPLEYKATLDVIRPKPADFGGDNLNVFWSEF
FFTAILVHIAFAVGVNPYKPK
VDVNGNFVNPEEDEPVDRRVTAPLCIGLTLGFLAFLGLATSGGVFNPALLFAPVIMS
NTWTKFWIYCTAEYAGGLIGGLL
QVFLLYKISY*
>jgilEnche1l1348
MAGETLRKIQSLLSEMVASFIFGFAVYSAILGSTIAQQPAAKVIIGLTVGFSAIGIIYSFS
DVTIAHFNPAITLAAILTG
KMGILCGLGYMLAQCVGFILAVCALLVCSPVGYKETLNVIRPAPAPFGADNLNVFFT
EFFLTAILVHIAFAVAVNPYRPK
VDTDGKFVDPDEKEPVDRRITAPLCIGLTLGFLAFMGLVTSGGAFNPGLTLAPVIMS
NTWQHFWLYLGAQYLGGLAGGLL
QVFVLYKLSSN*
>jgilNosce1l1809
MTRKWIKKLQSYIGEFFASFIFGFAVYTSIIGSAQTGQSAGPIIVALTIALSGVAIIYSFC
DITVAHFNPAITFSAMCFR
RLPFFGGIFIIIFQVAGFIIAGLASVAVLPGKYKNKLEIARPKRVADNVSRGRIFGTEFFL
TAILVYVAFAVGVNPYTPP
KDEHGDQLDPDEGLTEGRKITAPLAIGFTLGFCALLGIASSGGAFNPGIVLSPMILTG
TWDFWWVYLLGQFSGGLLGGGL
QRFLLYKIF*
>jgilVitco1l1625
MRINKAFLQSLLAECVCTFIFGYAIYSTSLNVKGPEVTSSDVFVPLAVGFSGIVVIYTF
LDHTICHFNPAITLSAILTFK
LPIIAGLCYIIAQEIGFILAACVAKVNFSLGWKETMDLISPGRVNPDVSNTSLFFTEFTL
TAILVFVAFENGINSRRNPE
VSLYGDKPQVDRSIVVPLTIGLTLGFLAFLAGTTSGGAFNPGLIFAPNLLGNTWNSDA
WEYYVGEFTGGLLGALIQVWLL
FK*
>jgilSprlo1l293
MVLTFPWRKLQSYLAEMAATFIFIFVVYSAVLTASMDELNGGPILEGLSVGFVTAAIIY
SFVDITIAHFNPAITFAAICL
GKLPIVTGITYIIFQLLGSMIACAVVLGCFPQPVSDILDIIRPSGVDSEVTTGEIMCTEL
FLSAILTFVAFAVAVNPYKS

PKYEDPEAEQLLSRPEDIKPDRSPFAPLVIGLIILVLALVGASTSGGAFNPAIVWSPVL
FSGVWNDSWKYWIAEFVGGVA
GGAVQVLILSRS*
>jgi|Enthe1|693
MQVSKKLIAQRVFAEFLCSCIFGFAVYSAILNTKSEEVSVSGTTVGLTVGFSGIALIYT
FADHSVAHFNPAITISTILTG
KIEIAMGICYVCAQLIGFLVASLLVVVCFPYGYSETLELITPAKVTEDLSTTSLFFTEFIL
SFILVFVAFEVGINAVREP
GVTLFIGEPQKDRSILAPLTIGLTLGFLGFLASTTSGGAFNPGIVFGPAILGSNYSDFW
VYIVSELSGGLLGALVQVFLL
FK*
>jgi|Heper1|351
MNILTKKSQSYFAEFLCTSIFGYCVYSAVASSKIDSYLSSTAVGLTVGFSGIVLIYTFVD
LTVAHFNPAITLAAICFNKL
DVIDGLVYILMQVLGFIFAALLIILSFPENDESVLNYIQTLRVSKEVNNLNLFVVEAILTFI
LVFVAFSVAINSKRDSDK
SLYGDEELPDRSIVAPLTIGLTLAFLAFVAPTTSGGLFNPGLAFAPSILIGDFEDVYVYII
AEFLGGIIGGFLQVFVFYK
*
>jgi|Vavcu1|1540
MPLSIQYSKIQSYIAECACSFIFGFTVYSAILTTTLNGIVAGPILIGLSIGFVSVVIIFLFVD
VTLAHFNPAITFAAVIF
RKVPIISGFFYIIAQGIGFMIASLVVQGCFPGSFRPLMNIIRPKQADDATTGEVICIEMF
LTGILVFSVFAMAANPYKKQ
KEERDKRELQIDPSESKIPDRSMFAPVVIGLTLGFLGYLGGSTSGGAFNPGIVWAPV
LFSGHWGDSWKYWVGEFVGGFGG
ALIQVILFYPFY*
>jgi|Traho1|265
MPLSIQYSKIQSYVAECACSFIFGFTVYSAILTTTLKNMVAGPILVGLSIGFVSIVIIFLFV
DVTLAHFNPAITFAAIVF
RRIPIISGFFYIIAQGIGFMIASLVVQGCFPGKFEPLMNIIRPKRVNDSTTGEMICIEMFL
TGILVFSVFVMAINPYKKQ
KDERDQRELHIDPSESKIPDRSRFAPIVIGLTLGFLGYLGGSTSGGAFNPGIVWAPVL
FSGHWGDSWKYWVGEFVGGCVG
AFIQIILFYPFY*
>jgi|Mitdap1|1629
MSSSSSSKVFLALRCVVGEFLVTFIFLSVVYAMLINTSRATDPVIAGTVIPGLVVSMVA
TAIIYSFADVSGAHFNPAVTM
GFIISGKMHPVKGVAYILAQLSASIAAAAFMFLIFPNNVAGWPKIAQVIPAVPPKSSPL
ATVLMMEFYLTFLLVYVIFAT
AVDIPAPPALRRLKNHFAPIAIGLTLGFLCYLGGSSSGGAFNPARVFGASVISGNFSS
HWVYWVGDLLGGSFAALLHTFV
FARKIGILSP*
>jgi|Entbi1|2250
MQYKYFKSYLAEFMASFIYGIAYNFAVITKVESIALVMLGVKCSIGYAFENLTLKHCNP
SVTLGCTFIGLLNIYTGIIYV
LFQIAGFICSAGIARLLYHDKYIIVYQKMQAENSVRIFLLEFFVNLLLGIIIIENIVYATGLI
QINKHYTTLSYQRTKHA
LPFSIGLTSGVGAFIASVASGGVFNSGIVFSVYLITNHTYNHFAAYICGEMLGSIAAAF
LVTYCIV*

>jgi|AmpWSBS2006_1|3286
MASVKKYAQIYVGEFLATFFFLFAAQAAAYNEKLLSSPILSALVVAIAGTGVVATFGTIS
GAHFNPAVTIGAIVGGRIGI
VSGIVYIVLQVLAGVLATTTLCALYPADFETVAKNLVVAPPSTETEDVIRALVMESVLT
FILVFVVYSVALGLPSEKERH
EGKHGGIVTEEDEPPSEERVARKNSLAPLAIGFTLGFLVPIGGPASGGAFNPARVTG
PALLAGEYNGVWIYWVGGIIGPA
IAAICWVFLFF*
>jgi|Pseneu1|3635
MPTSFRISYVQTYFAEFICSFIFGFTVYSAIVLTTLKEMAAGQILIGVAIGFVSISIIWAF
CDITVAHFNPAITFATVIF
CKLPIIKGIFYIIAQGLGFMLAAVVVLGSFPGNWRQIMNIIRPKPADGVVTGEVICIEIFL
TGILVFIVFCSAVNSYKQQ
KDKKNLEQLGENTSDIPDRTMFIPLIIGSTLGFLGLLGGSTSGGAFNPAIVWAPVLFS
GVWRDSWKYWVGQFVGAFGAGV
IQMVVFYPFN*
