# Supplementary material for: Major Intrinsic Proteins in Fungi: A Special Emphasis on the XIP Subfamily
Source: J Fungi (Basel). 2025 Jul 21;11(7):543. doi: 10.3390/jof11070543 (PMC12300952; doi:10.3390/jof11070543)
Supplement: Supplementary file 1 [file jof-11-00543-s001.zip › jof-3752183_Supplementary_Table_S1.pdf]

Supplementary Table S1. Set of XIP sequences.

Taxonomic phyla [Phyla number]	fungal organism	User name for this work	Protein Id	Web resource
Pucciniomycotina [I]	**Cronartium comandrae**	CorcoI	596144	JGI
	**Cronartium harknessii**	Corhal013	JAVIAN010000007.1	NCBI
		Corhal008	JAVIAN010000013.1	NCBI
	**Cronartium quercuum f. sp. Fusiforme**	Croqu845I	649845	JGI
		Croqu6585I	684658	JGI
	**Cronartium ribicola**	Crori133I	AWVX01018133.1	NCBI
		Crori064I	AWVX01056064.1	NCBI
	**Cronartium ribicola Cypress**	Crocy129I	1044129	JGI
		Crocy696I	819696	JGI
	**Melampsora aecidioides**	MelaeI	MWRI01041652.1	NCBI
	**Melampsora abietis canadensis**	Melab479I	MWRH01003479.1	NCBI
		Melab876I	MWRH01021876.1	NCBI
	**Melampsora allii populina**	Melal142I	1308142	JGI
		Melal459I	1815459	JGI
	**Melampsora americana**	Melam101I	1054101	JGI
		Melam562I	410562	JGI
	**Melampsora x columbiana Clatskanie**	Melco417I	551417	JGI
		Melco202I	712202	JGI
	**Melampsora laricis**	Mella062I	1794062	JGI
		Mella798I	1814798	JGI
	**Melampsora lini**	Melli368I	212368	JGI
		Melli536I	201536	JGI
	**Melampsora medusae deltoidae**	Melde443I	887443	JGI
		Melde642I	757642	JGI
	**Melampsora medusae tremuloidae**	Meltr181I	1061181	JGI
		Meltr185I	1119185	JGI
	**Melampsora occidentalis**	Meloc843I	MWRL01005843.1	NCBI
		Meloc257I	MWRL01023257.1	NCBI
	**Phakopsora pachyrhizi**	Phapa268I	6634268	JGI
		Phapa251I	8686251	JGI
	**Puccinia coronata avenae**	PuccoI	PGCI01000008.1	NCBI
	**Puccinia graminis f. sp. Tritici**	PucgrI	PGTT_08211	JGI
	**Puccinia hordei**	PuchoI	RDRW01000216.1	NCBI
	**Puccinia sorghi**	PucsoI	LAVV01012517.1	NCBI
	**Puccinia striiformis f. sp. Tritici**	PucstI	PELB01000609.1	NCBI
	**Puccinia triticina**	PuctrI	AZRP01004910.1	NCBI
	**Uromyces appendiculatus**	UroapI	GACI01002254.1	NCBI
	**Uromyces viciae fabae**	UroviI	JNCO01016183.1	NCBI
Agaricomycetes [III]	**Botryobasidium botryosum**	BotboIII	AYEP01000646.1	NCBI
	**Ceratobasidium_anastomosis**	CeranIII	153017	JGI
	**Ceratobasidium sp.**	CerspIII	1268080	JGI
	**Flagelloscypha sp.**	FlaspIII	1517871	JGI
	**Mycena alexandri**	MycalIII	1170102	JGI
	**Mycena filopes**	MycfiIII	1227459	JGI
	**Mycena leptocephala**	MycleIII	2158262	NCBI
	**Mycena metata**	MycmeIII	1316736	NCBI
	**Mycena polygramma**	MycpoIII	1315397	NCBI
	**Mycena vitilis**	MycviIII	985602	JGI
	**Piloderma byssinum**	PilbyIII	340448	JGI
	**Piloderma croceum**	Pilcr880III	JMDN01001880.1	JGI
	**Piloderma croceum**	Pilcr057III	JMDN01001057.1	NCBI
	**Piloderma olivaceum**	PiloIIII	571376	JGI
	**Piloderma sphaerosporum**	PilspIII	867689	JGI
	**Ramariopsis corniculata**	RamcoIII	CAYFCV010000012.1	NCBI
	**Rhizoctonia solani**	RhisoIII	g3936.t1	JGI
	**Stereum hirsutum**	SteHIII	169569	JGI
	**Thanatephorus cucumeris**	ThacuIII	fgenesh1_pg.1_#_258	NCBI
	**Thelephora ganbajun**	ThegaIII	3168519	JGI
	**Thelephora terrestris**	TheteIII	1106481	JGI
	**Tricholoma saponaceum**	TrisaX	QLOJ01013304.1	NCBI
	**Tricholoma sp.**	TrispX	QMFE01024132.1	NCBI
	**Tulasnella sp. 419**	Tulsp419III	1510334	JGI
	**Tulasnella sp. 425**	Tulsp425III	1898559	JGI
	**Tulasnella sp. 427**	Tulsp427III	465444	JGI
	**Tulasnella calospora**	TulcaIII	21817	JGI

**Dacrymycetes [IV]**	**Dacryonaema rufum**	DacruIV	2420693	NCBI
	**Unilacryma unispora**	UniunIV	958340	JGI
**Tremellomycetes [V]**	**Apiotrichum porosum**	ApipoV	NW_021167125.1	NCBI
	**Cryptococcus sp.**	CryspV	BCLC01000005.1	NCBI
	**Cryptococcus terricola**	CryteV	810440	NCBI
	**Fellomyces penicillatus**	FelpeV	277600	JGI
	**Goffeauzyma gilvescens**	GofgiV	JANGFI010000446.1	JGI
	**Saitozyma podzolica**	SaipoV	RSCD01000014.1	NCBI
	**Saitozyma sp.**	SaispV	BCLC01000005.1	NCBI
	**Solicoccozyma terricola**	SolteV	MATT01000344.1	NCBI
	**Trichosporon asahii var. asahii**	TriasV	CBS 2479 EJT51195	JGI
	**Trichosporon brassicae**	TribrV	BCJI01000006.1	NCBI
	**Trichosporon chiarellii**	TrichV	55848	JGI
	**Trichosporon coremiiforme**	TricoV	JXYL01000023.1	NCBI
	**Trichosporon faecale**	TrifaV	JXYK01000002.1	NCBI
	**Trichosporon guehoae**	TriguV	BCJX01000001.1	NCBI
	**Trichosporon laibachii**	TrilaV	BCKV01000001.1	NCBI
	**Trichosporon montevideense**	TrimoV	BCFV01000004.1	NCBI
	**Vanrija humicola**	VanhuV	BCJF01000001.1	NCBI
**Pezizomycetes [VIII]**	**Choiromyces venosus**	ChoveVII	PZQU01000748.1	NCBI
	**Discina gigas**	DisgiVII	JBBBZM010000119.1	NCBI
	**Gyromitra antarctica**	Gyran137VII	JBALZX010000137.1	NCBI
		Gyran195VII	JBALZX010000195.1	NCBI
	**Gyromitra esculenta**	Gyres632VII	550632	JGI
		Gyres966VII	517966	JGI
	**Gyromitra infula**	Gyrin829VII	926829	JGI
	**Gyromitra sichuanensis**	Gyrsi216VII	JBALYS010000216.1	NCBI
		Gyrin200VII	910200	JGI
		Gyrsi043VII	JBALYS010000043.1	NCBI
	**Gyromitra splendida**	Gyrspl166VII	JBALYC010001166.1	NCBI
	**Gyromitra sp9**	Gyrsp290VII	JBALXX010000290.1	NCBI
		Gyrspl012VII	JBAMAE010000012.1	NCBI
		Gyrsp243VII	JBALXX010000243.1	NCBI
	**Gyromitra venenata**	Gyrven059VII	JBALZH010000059.1	NCBI
		Gyrven011VII	JBALZR010000011.1	NCBI
	**Hydnotrya bailii**	HydbaVII	JBALYF010003432.1	NCBI
	**Hydnotrya cerebriformis**	HydceVII	JBALZS010000607.1	NCBI
	**Hydnotrya cubispora**	HydcuVII	JBALZY010000715.1	NCBI
	**Hydnotrya tulasnei**	HydtuVII	JBALYD010000069.1	NCBI
	**Hydnotrya variiformis**	HydvaVII	JBALZC010001212.1	NCBI
	**Hydnotrya sp.1**	Hydsp1VII	JBALZE010000688.1	NCBI
	**Hydnotrya sp.2**	Hydsp2VII	JBALZD010000422.1	NCBI
	**Hydnotrya sp.2**	Hydsp3VII	JBALYM010000116.1	NCBI
	**Kalapuya brunnea**	KalbrVII	881142	JGI
	**Leucangium carthusianum**	LeucaVII	1021319	JGI
	**Marcelleina donadinii**	MardoVII	JBALXU010000144.1	NCBI
	**Tuber aestivum**	TubaeVII	CZPR01005160.1	NCBI
	**Tuber borchii**	TubboVII	1043958	JGI
	**Tuber brumale**	TubbrVII	JACCEG010000078.1	NCBI
	**Tuber calosporum**	TubcalVII	QFET01012817.1	NCBI
	**Tuber canaliculatum**	TubcanVII	892231	NCBI
	**Tuber gibbosum**	TubgiVII	1119262	NCBI
	**Tuber indicum**	TubinVII	587124	NCBI
	**Tuber magnatum**	TubmaVII	PYWC01000076.1	NCBI
	**Tuber melanosporum**	TubmelVII	1698	JGI
	**Tuber mesentericum**	TubmesVII	3585653	JGI
	**Tuber microsphaerosporum**	TubmiVII	QOVD01009807.1	NCBI
	**Tuber umbilicatum**	TubumVII	QLOI01000906.1	NCBI
	**Verpa bohemica**	VerboVII	JAMGYS010000008.1	NCBI
	**Verpa conica**	VercoVII	329707	JGI

Orbiliomycetes [VIII]	**Arthrobotrys conoides**	ArtcoVIII	JAVHJM010000005.1	NCBI
	**Arthrobotrys flagrans**	ArtflVIII	SAEB01000003.1	NCBI
	**Arthrobotrys entomopaga**	ArtenVIII	LDDX01000196.1	NCBI
	**Arthrobotrys iridis**	ArtirVIII	JAJTTS010000008.1	NCBI
	**Arthrobotrys megalospora**	ArtmeVIII	JAVHJN010000010.1	NCBI
	**Arthrobotrys oligospora**	ArtolVIII	EGX52763	NCBI
	**Arthrobotrys sinensis**	ArtsiVIII	JAJTTV010000007.1	NCBI
	**Dactylellina cionopaga**	DactciVIII	LDUC01000099.1	NCBI
	**Dactylellina haptotyla**	DacthaVIII	AQGS01000918	NCBI
	**Duddingtonia flagrans**	DudflVIII	SAEB01000003.1	NCBI
	**Monacrosporium haptotylum**	MonhaVIII	9646	NCBI
	**Orbilia ellipsospora**	OrbelVIII	JAVHJO010000012.1	NCBI
	**Orbilia oligospora**	OrbolVIII	JAABOJ010000004.1	NCBI
	**Arthrocladium fulminans**	ArtfuIX	QXWJ01000009.1	NCBI
	**Aspergillus acidus**	AspacilX	338208	JGI
	**Aspergillus aculeatus**	Aspacu958IX	63958	JGI
		Aspacu679IX	64679	JGI
		Aspacu287IX	44287	JGI
	**Aspergillus brasiliensis**	AspbraIX	56152	JGI
	**Aspergillus brunneoviolaceus**	AspbruIX	390164	JGI
	**Aspergillus carbonarius**	AspcaIX	696296	JGI
	**Aspergillus clavatus**	AspclIX	AAKD03000041.1	NCBI
	**Aspergillus costaricaensis**	AspcoIX	220245	JGI
	**Aspergillus eucalypticola**	AspeuIX	341370	JGI
	**Aspergillus fijiensis**	AspfiIX	460023	JGI
	**Aspergillus flavus**	Aspfl016IX	AAIH02000016.1	NCBI
		Aspfl095IX	AAIH02000095.1	NCBI
		Aspfl540IX	AAIH02000540.1	NCBI
	**Aspergillus glaucus**	AspglIX	10188	JGI
	**Aspergillus heteromorphus**	AspheIX	160118	JGI
	**Aspergillus homomorphus**	AsphoIX	440013	JGI
	**Aspergillus ibericus**	AspibIX	445835	JGI
	**Aspergillus japonicus**	AspjaIX	386949	JGI
	**Aspergillus kawachii**	AspkaIX	GAA90923.1	NCBI
	**Aspergillus lacticoffeatus**	AsplaIX	194256	JGI
	**Aspergillus luchuensis**	AspluIX	BCWF01000040.1	NCBI
	**Aspergillus neoniger**	AspneIX	52126	JGI
	**Aspergillus niger**	Aspni015IX	ACJE01000015.1	NCBI
		Aspni003IX	ACJE01000003.1	NCBI
	**Aspergillus ochraceoroseus**	AspocIX	3504	JGI
	**Aspergillus orizae**	Aspor602IX	AKXN01000602.1	NCBI
		Aspor395IX	AKXN01000365.1	NCBI
	**Aspergillus parasiticus**	AsppaIX	JMUG01001154.1	NCBI
	**Aspergillus phoenicis**	AspphIX	190043	JGI
	**Aspergillus piperis**	AsppiIX	150028	JGI
	**Aspergillus saccharolyticus**	AspsaIX	18238	JGI
	**Aspergillus Sclerotiicarbonarius**	Aspsc219IX	14219	JGI
	**Aspergillus sclerotioniger**	Aspsc798IX	10798	JGI
	**Aspergillus steynii**	AspstIX	10121	JGI
	**Aspergillus sydowii**	AspsyIX	11556	JGI
	**Aspergillus terreus**	Aspte055IX	AAJN01000055.1	NCBI
		Aspte140IX	AAJN01000140.1	NCBI
	**Aspergilus tubingensis**	Asptu957IX	13957	JGI
		Asptu6547IX	60654	JGI
	**Aspergillus uvarum**	AspuvIX	100080	JGI
	**Aspergillus vadensis**	AspvaIX	10190	JGI
	**Aspergillus versicolor**	Aspve917IX	12917	JGI
		Aspve116IX	36116	JGI
	**Aspergillus violaceofuscus**	AspviIX	11196	JGI
	**Aspergilus wentii**	AspweIX	10022	JGI
	**Aspergilus zonatus**	AspzoIX	16031	JGI
	**Byssochlamys nivea**	BysniIX	QEIL01000087.1	NCBI
	**Byssochlamys spectabilis**	BysspeIX	56744	JGI
	**Byssochlamys sp**	BysspIX	PNEM01000009.1	NCBI
	**Capronia coronata**	CapcoIX	XM_007725951.1	NCBI
	**Capronia epimyces**	CapepIX	XM_007734026.1	NCBI
	**Capronia fungicola**	CapfuIX	MIX6013_71_62	NCBI
	**Capronia semiimmersa**	CapseIX	PV04_07780T0	JGI
	**Chaetothyriales sp.**	Chasp022IX	JADCQX010000022.1	NCBI
	**Chaetothyriales sp.**	Chasp769IX	896769	JGI
	**Cladophialophora bantiana**	ClabaIX	Z519_07118T0	JGI
	**Cladophialophora carrionii**	Claca022IX	AOFF01000022.1	NCBI
		Claca032IX	AOFF01000032.1	NCBI
	**Cladophialophora exuberans**	ClaexIX	JANSVK010000039.1	NCBI
	**Cladophialophora immunda**	ClaimIX	PV07_11521T0	JGI
	**Cladophialophora psammophila**	ClapsIX	XM_007743202.1	NCBI
	**Cladophialophora yegresii**	Claye324IX	XM_007761324.1	NCBI
		Claye003IX	AMGW01000003.1	NCBI
	**Cyphellophora europaea**	Cypeu401IX	XM_008722401.1	NCBI

Eurotiomycetes [IX]		Cypeu698IX	JADCRJ010001698.1	NCBI
	**Dactylospora haliotrepha**	DachaIX	739116	NCBI
	**Elaphomyces granulatus**	Elagr381IX	NPHW01004381.1	NCBI
		Elagr383IX	NPHW01004383.1	NCBI
	**Emergomyces orientalis**	EmeorIX	MOWL01000049.1	NCBI
	**Epibryaceae sp.**	EpispIX	73892	NCBI
	**Eurotiomycetes sp.**	Eursp049IX	PQME02000049.1	NCBI
	**Eurotiomycetes sp.**	Eursp167IX	PQMF01000167.1	NCBI
	**Eurotiomycetes sp.**	Eursp047IX	PCFI01000047.1	NCBI
	**Eurotium herbariorum**	EurheIX	LSTL01000174.1	JGI
	**Eurotium rubrum**	EurruIX	385652	JGI
	**Exophiala alcalophila**	ExoalIX	CE43538_4773	JGI
	**Exophiala aquamarina**	ExoaqIX	AMGV01000003	NCBI
	**Exophiala bonariae**	ExoboIX	JAVRRD010000008.1	NCBI
	**Exophiala calicioides**	ExocaIX	BCHZ01000008.1	NCBI
	**Exophiala mesophila**	ExomeIX	JSEI01000038.1	NCBI
	**Exophiala oligosperma**	ExoolIX	PV06_00344T0	JGI
	**Exophiala sideris**	ExosiIX	PV11_01447T0	JGI
	**Exophiala sp1**	Exosp008IX	BCHZ01000008.1	NCBI
	**Exophiala sp2**	Exosp005IX	JALDXI010000005.1	NCBI
	**Exophiala spinifera**	ExospiIX	PV08_04213T0	JGI
	**Exophiala viscosa**	ExoviIX	622960	JGI
	**Exophiala xenobiotica**	ExoxeIX	PV05_04699T0	JGI
	**Floridaphiala radiotolerans**	FloraIX	JAKLMZ010000026.1	JGI
	**Fonsecaea erecta**	FonerIX	LVYI01000002.1	NCBI
	**Fonsecaea monophora**	FonmoIX	AYO21_05312T0	JGI
	**Fonsecaea multimorphosa**	FonmuIX	Z520_00061T0	JGI
	**Fonsecaea nubica**	FonnuIX	AYO20_09531T0	JGI
	**Fonsecaea pedrosoi**	FonpeIX	Z517_02914T0	JGI
	**Fonsecaea pugnacius**	FonpuIX	WJFF01000172.1	NCBI
	**Gymnascella aurantiaca**	GymauIX	147511	JGI
	**Helicocarpus griseus**	HelgrIX	PDNB01000140.1	NCBI
	**Incumbomyces delicatus**	IncdeIX	JACJVS010000009.1	NCBI
	**Incumbomyces lentus**	IncleIX	JACJVT010000004.1	NCBI
	**Knufia fluminis**	KnuflIX	JAKLMC010000001.1	NCBI
	**Knufia peltigerae**	KnupeIX	JAPDRN010000001.1	NCBI
	**Knufia sp.**	KnuspIX	JAPDRQ010000056.1	NCBI
	**Monascus purpureus**	MonpuIX	490511	JGI
	**Monascus ruber**	MonruIX	477925	JGI
	**Paecilomyces dactylethromorphus**	PaedaIX	JAPVCF010000017.1	NCBI
	**Paecilomyces niveus**	PaeniIX	77609	JGI
	**Penicilliopsis zonata**	PenzoIX	NW_019208919.1	NCBI
	**Penicillium antarcticum**	PenanIX	PENANT_c009G02731T0	JGI
	**Penicillium aurantiogriseum**	PenauIX	ALJY01003980.1	NCBI
	**Penicillium bilaiae**	PenbiIX	fgenesh1_pg.C_1_t20192	NCBI
	**Penicillium brasilianum**	PenbraIX	LJBN01000112.1	NCBI
	**Penicillium brevicompactum**	PenbreIX	Genemark1.C_4_t10182	JGI
	**Penicillium camemberti**	PencaIX	CBVV010000374.1	NCBI
	**Penicillium chrysogenum**	PenchIX	XP_002562918.1	NCBI
	**Penicillium citrinum**	PenciIX	BCKA01000003.1	NCBI
	**Penicillium expansum**	PenexIX	CE115384_246	JGI
	**Penicillium fellutanum**	PenfeIX	Genemark1.C_4_t10005	JGI
	**Penicillium freii**	PenfrIX	LLXE01000016.1	NCBI
	**Penicillium glabrum**	PenglIX	fgenesh1_pg.C_2_t30110	JGI
	**Penicillium janthinellum**	PenjaIX	CE143691_1747	JGI
	**Penicillium lanosocoeruleum**	PenlaIX	gm1.7745_g	JGI
	**Penicillium marneffei**	PenmaIX	XP_002149425.1	NCBI
	**Penicillium nalgiovense**	PennaIX	PENNAL_c0011G07886T0	JGI
	**Penicillium nordicum**	PennoIX	JNNR01000805.1	NCBI
	**Penicillium occitanis**	PenocIX	NPFJ01000207.1	NCBI
	**Penicillium paxilli**	PenpaIX	gb|AOTG01000150.1	NCBI
	**Penicillium raistrickii**	PenraIX	CE131021_3024	JGI
	**Penicillium sclerotiorum**	PenscIX	MJCA01000203.1	NCBI
	**Penicillium solitum**	PensoIX	PENSOL_c012G07629T0	JGI
	**Penicillium steckii**	PenstIX	PENSTE_c004G07697T0	JGI
	**Penicillium swiecickii**	PenswIX	Genewise1Plus.C_510066	JGI
	**Penicillium thymicola**	PenthIX	e_gw1.63.2.1	JGI
	**Phaeomoniellales sp.**	PhaspIX	sh1_kg.11_#_64_#_TRINITY_DN6555_c0_	NCBI
	**Phialophora attae (CBS 131958 strain)**	Phiat521IX	5521	JGI
	**Phialophora attae (AB675_9106 strain)**	Phiat614IX	XM_018149614.1	NCBI
	**Phialophora americana**	PhiamIX	JYCC01000047.1	NCBI
	**Phialophora attinorum**	PhiatIX	LFJN01000009.1	NCBI
	**Phialophora chinensis**	PhichIX	JADWPV010000024.1	NCBI
	**Phialophora expanda**	PhiexIX	JADWPR010000004.1	NCBI
	**Phialophora macrospora**	PhimaIX	JYCC01000047.1	NCBI
	**Phialophora tarda**	PhitaIX	JADWPL010000007.1	NCBI
	**Phialophora verrucosa**	PhiveIX	MSED01000002.1	NCBI
	**Polytolypa hystricis**	PolhyIX	PDNA01000007.1	NCBI
	**Rhinocladiella mackenziei**	RhimaIX	XM_013413496.1	NCBI

**Rhinocladiella similis**	RhisiIX	JAMRYE010000010.1	NCBI
**Talaromyces aculeatus**	TalacIX	439497	JGI
**Talaromyces adpressus**	TaladIX	NHZS01000476.1	NCBI
**Talaromyces amestolkiae**	TalamIX	MIKG01000012.1	JGI
**Talaromyces cellulolyticus**	TalceIX	BBPS01000035.1	JGI
**Talaromyces funiculosus**	TalfuIX	JAMADO010000012.1	NCBI
**Talaromyces liani**	TalliIX	JAKLNL010000007.1	NCBI
**Talaromyces marneffei**	TalmaIX	JPOX01000023.1	JGI
**Talaromyces proteolyticus**	TalprIX	Genewise1.C_10_t30436	JGI
**Talaromyces purpureogenus**	TalpuIX	LIAB01000861.1	JGI
**Talaromyces sp.**	TalspIX	68547	NCBI
**Talaromyces stipitatus**	TalstIX	XM_002484703.1	NCBI
**Talaromyces verruculosus**	TalveIX	LHCL01000025.1	NCBI
**Thermomyces stellatus**	Thest136IX	JBAGCR010000136.1	NCBI
	Thest038IX	JBAGCR010000038.1	NCBI
**Aaosphaeria arxii**	AaoarX	462612	JGI
**Aaosphaeria pasadenensis**	AaopaX	JAKLMB010000013.1	JGI
**Acrocalymma vagum**	AcrvaX	JAQGEP010000050.1	NCBI
**Amniculicola lignicola**	AmnliX	461298	JGI
**Aquilomyces patris**	AqupaX	gm1.14986_g	JGI
**Aureobasidium melanogenum**	AurmeX	JAHFYE010000001.1	NCBI
**Baudoinia compniacensis**	BaucoX	107398	JGI
**Baudoinia panamericana**	Baupa011X	AEIF01000011.1	NCBI
	Baupa591X	JADCRE010002591.1	NCBI
	Baupa791X	JADCRF010000791.1	NCBI
**Beverwykella pulmonaria**	BevpuX	BCHH01000021.1	NCBI
**Bimuria novae-zelandiae**	BimnzX	474745	JGI
**Bipolaris bicolor**	BipbiX	JAODYD010000014.1	NCBI
**Bipolaris cookei**	BipcoX	NRSV01000083.1	NCBI
**Bipolaris sorokiniana**	BipsoX	RCTM01000002.1	NCBI
**Bipolaris victoriae**	BipviX	AMCY02000014.1	NCBI
**Bipolaris zeicola**	BipzeX	AMCN01000071.1	NCBI
**Cenococcum geophilum**	CengeX	CE86733_2252	JGI
**Cladosporium sphaerospermum**	ClasphX	AIIA02000467.1	NCBI
**Cladosporium sp.**	ClaspX	PEGC01000012.1	NCBI
**Clohesyomyces aquaticus**	CloaqX	495163	JGI
**Cochliobolus carbonum**	CoccaX	51209	JGI
**Cochliobolus heterostrophus**	CocheX	AIHU01000206.1	NCBI
**Cochliobolus lunatus**	CocluX	48044	JGI
**Cochliobolus miyabeanus**	CocmiX	30348	JGI
**Cochliobolus sativus**	CocsaX	194140	JGI
**Cochliobolus victoriae**	CocviX	AMCY01000045.1	NCBI
**Corynespora cassicola**	CorcaX	675870	JGI
**Cucurbitaria berberidis**	CucbeX	394415	JGI
**Curvularia eragrostidis**	CurerX	JAIRCK010000004.1	NCBI
**Curvularia geniculata**	CurgeX	PQMW01000043.1	NCBI
**Curvularia hawaiiensis**	CurhaX	JAODYC010000020.1	NCBI
**Curvularia lunata**	CurluX	JFHG01002021.1	NCBI
**Curvularia papendorfii**	CurpaX	JXCC01000129.1	NCBI
**Curvularia spicifera**	CurspiX	JAODYB010000001.1	NCBI
**Curvularia sp.**	CurspX	JPSZ01000004.1	NCBI
**Darksidea phi**	DarphX	JASJFT010000186.1	JGI
**Dendryphion nanum**	DennaX	130204	JGI
**Didymosphaeria enalia**	DidenX	JAAEIZ010000236.1	NCBI
**Didymocrea sadasivanii**	DidsaX	515608	JGI
**Diplodia pinea**	DippiX	JHUM01000697.1	NCBI
**Diplodia seriata**	DipseX	MSZU01000074.1	NCBI
**Elsinoe ampelina**	ElsamX	223221	JGI
**Elsinoe arachidis**	ElsarX	JAAPAX010000007.1	NCBI
**Elsinoe australis**	ElsauX	NHZQ01000419.1	NCBI
**Elsinoe batatas**	ElsbaX	JARDAA010000044.1	NCBI
**Elsinoe fawcettii**	ElsfaX	WLYZ01000002.1	NCBI
**Elsinoe necatrix**	ElsneX	JANZYI010000007.1	NCBI
**Elsinoe perseae**	ElspeX	JAESVG020000004.1	NCBI
**Eremomyces bilateralis**	ErebiX	410647	JGI
**Exserohilum rostratum**	ExsroX	JAHALU010000013.1	NCBI
**Exserohilum turcica**	ExstuX	AIHT01001306.1	NCBI
**Friedmanniomyces endolithicus**	FrienX	JASUXR010000069.1	NCBI
**Friedmanniomyces simplex**	FrisiX	JASUXR010000069.1	NCBI
**Gloniopsis sp.**	GlospX	h1_kg.23_#_323_#_TRINITY_DN3912_c0	NCBI
**Glonium stellatum**	Glost212X	LKAO01004212.1	NCBI
	Glost555X	e_gw1.9555.9.1	JGI
**Herpotrichia sp.**	Hersp482X	543482	JGI
**Herpotrichiellaceae sp3F**	Hersp678X	507678	JGI
**Herpotrichiellaceae sp7F**	Hersp404X	646404	JGI
**Herpotrichiellaceae sp**	Hersp63X	fgenesh1_pm.32_#_63	JGI
**Jahnula aquatica**	JahaqX	443455	JGI
**Lasiodiplodia theobromae**	LasthX	MDYX01000001.1	NCBI
**Lecanosticta acicola**	LecacX	AWYC01003362.1	NCBI

	**Lentithecium fluviatile**	LenflX	CE152864_129	JGI
	**Lepidopterella palustris**	LeppaX	362984	JGI
	**Leptosphaerulina australis**	LepauX	JALRMI010001279.1	NCBI
	**Leptoxyphium fumago**	LepfuX	LSHF01000008.1	NCBI
	**Lophiostoma macrostomum**	Lopma012X	JAAGLC010000012.1	NCBI
	**Lophiotrema macrostomum**	Lopma475X	620475	JGI
	**Lophiotrema nucula**	LopnuX	642863	JGI
	**Lophium mytilinum**	LopmyX	CE21995_2882	JGI
	**Massariosphaeria phaeospora**	MasphX	586036	JGI
	**Melanomma pulvis-pyrius**	MelpuX	1840047	JGI
	**Microthyrium microscopicum**	MicmiX	456654	NCBI
	**Mycosphaerella fijiensis**	MycfiX	XM_007926717.1	JGI
	**Myriangiaceae sp.**	MyrspX	268091	JGI
	**Myriangium duriaei**	Myrdu719X	253719	JGI
		Myrdu250X	315250	JGI
	**Mytilinidiaceae sp.**	MytspX	662304	JGI
	**Mytilinidion resinicola**	MytreX	404319	JGI
	**Nigrograna mackinnonii**	NigmaX	JGVQ01000072.1	NCBI
**Dothideomycetes [X]**	**Nothophaeocryptopus gaeumannii**	Notga905X	MWSP01000905.1	NCBI
		Notga062X	MWSP01000062.1	NCBI
	**Ochroconis constricta**	OchcoX	AZYM01000081.1	NCBI
	**Ophiobolus disseminans**	OphdiX	446023	JGI
	**Ophiosphaerella herpotricha**	OphheX	JALNQK010000009.1	NCBI
	**Ophiosphaerella korrae**	OphkoX	JALNQF010000090.1	NCBI
	**Ophiosphaerella narmari**	OphnaX	JALNQB010000002.1	NCBI
	**Paracamarosporium sp.**	ParspX	JACVRA010000128.1	NCBI
	**Pallidocercospora crystalline**	Palcr108X	QQNG01000108.1	NCBI
	**Pallidocercospora crystallina**	Palcr044X	QQNG01000044.1	NCBI
	**Paraconiothyrium sporulosum**	ParspoX	1208779	JGI
	**Parafenestella ontariensis**	ParonX	JAKGDA010000024.1	NCBI
	**Paraphaeosphaeria sporulosa**	ParaspoX	NW_017253369.1	NCBI
	**Paraphoma chrysanthemicola MPI_GEGE**	Parch062X	40062	JGI
	**Paraphoma chrysanthemicola MPI_SDFR**	Parch241X	578241	JGI
	**Paraphoma sp.**	ParapspX	BCLK01000150.1	NCBI
	**Peltaster fructicola**	PelfrX	QIW97353.1	NCBI
	**Periconia digitata**	PerdiX	CAOQHR010000002.1	JGI
	**Periconia macrospinosa**	PermaX	654005	NCBI
	**Periconia sp.**	PerspX	JASNQO010000030.1	NCBI
	**Phaeosphaeria sp.**	PhaspX	JAGTJN010000002.1	NCBI
	**Phaeosphaeriaceae sp.**	PhaespX	CE412503_528	JGI
	**Phaeosphaeria poagena**	PhapoX	sh1_kg.2_#_700_#_TRINITY_DN4583_c1_	JGI
	**Pleosporaceae sp.**	Plesp002X	JAGTJN010000002.1	NCBI
	**Pleosporales sp.**	Plesp716X	AJMS01006716.1	NCBI
	**Pleosporineae sp.**	Plesp068X	CALMRN010000068.1	NCBI
	**Polychaeton citri**	Polci047X	304047	JGI
		Polci070X	308070	JGI
	**Polyplosphaeria fusca**	PolfuX	CE250808_778	JGI
	**Preussia sp.**	PrespX	LJJI01000212.1	NCBI
	**Pseudocercospora cruenta**	PsecruX	JAASFE010000791.1	NCBI
	**Pseudocercospora crystallina**	PsecryX	QQNG01000108.1	NCBI
	**Pseudocercospora fijiensis**	PsefiX	AIHZ01000223.1	NCBI
	**Pseudocercospora fuligena**	PsefuX	JABCIY010000235.1	NCBI
	**Pseudocercospora macadamiae**	PsemaX	JABCIY010000235.1	NCBI
	**Pseudocercospora pini-densiflorae**	PsepiX	AWYD01006552.1	NCBI
	**Pseudomonodictys sp.**	PsespX	JAFHBV010000158.1	NCBI
	**Pseudopyrenochaeta lycopersici**	PselyX	NHZP01000016.1	NCBI
	**Pyrenochaeta sp.**	PyrspX	ASRS01005673.1	NCBI
	**Pyrenochaeta inflorescentiae**	PyrinX	206982	JGI
	**Pyrenochaeta lycopersici**	PyrlyX	h1_kg.9_#_315_#_TRINITY_DN6511_c0_	JGI
	**Rachicladosporium sp.**	RacanX	NAEU01000674.1	NCBI
	**Rachicladosporium antarcticum**	RacspX	NAJO01000026.1	NCBI
	**Racodium therryanum**	RacthX	BLYF01000118.1	NCBI
	**Ramichloridium luteum**	RamluX	MTSC01000074.1	NCBI

	**Recurvomyces mirabilis**	RecmiX	XM_064841355.1	NCBI
	**Rhizodiscina lignyota**	Rhili585X	72585	JGI
		Rhili008X	JAADKO010000008.1	NCBI
	**Rhytidhysteron rufulum**	RhyruX	4577	JGI
	**Septoria populicola**	SeppoX	42516	JGI
	**Setomelanomma holmii**	SethoX	JAADKM010000054.1	NCBI
	**Setosphaeria turcica**	SettuX	1074264	JGI
	**Setophoma terrestris**	SetteX	Genewise1.C_2_t30018	JGI
	**Sphaceloma murrayae**	SphmuX	NKHZ01000012.1	NCBI
	**Sphaerulina populicola**	SphpoX	AIDU01002179.1	NCBI
	**Stagonospora sp.**	StaspX	246360	JGI
	**Stomiopeltis betulae**	StobeX	e_gw1.15.186.1	JGI
	**Teratosphaeriaceae_sp**	TerspX	gm1.2473_g	JGI
	**Tothia fuscella**	TotfuX	JAAEJU010000114.1	JGI
	**Trematosphaeria pertusa**	TrepeX	570037	JGI
	**Trichodelitschia bisporula**	TribiX	557937	JGI
	**Trypethelium eluteriae**	TryelX	JAGFMJ010000059.1	NCBI
	**Venturia asperata**	VenasX	QWWM01000051.1	NCBI
	**Venturia aucupariae**	VenauX	QWZN01000177.1	NCBI
	**Venturia carpophila**	VencaX	MECS01000010.1	NCBI
	**Venturia inaequalis**	VeninX	QWZI01002212.1	JGI
	**Venturia nashicola**	VennaX	SNSD01000002.1	NCBI
	**Venturia populina**	VenpoX	Genemark4.C_250024	NCBI
	**Venturia pyrina**	VenpyX	JEMP01000097.1	NCBI
	**Verruconis gallopava**	VergaX	124894	JGI
	**Verruconis sp.**	VerspX	SCGS01002764.1	NCBI
	**Verruculina enalia**	VerenX	615407	JGI
	**Viridothelium virens**	VirviX	JAAEIX010000043.1	JGI
	**Xanthoria parietina**	XanpaX	CAZZRP010004709.1	NCBI
	**Zalaria obscura**	ZalobX	JAKLMX010000004.1	NCBI
	**Zalaria sp.**	ZalspX	BPUN01000007.1	NCBI
	**Zasmidium angulare**	Zasan016X	NHNX01000016.1	NCBI
		Zasan007X	NHNX01000007.1	NCBI
	**Zasmidium cellare**	Zasce658X	71658	JGI
		Zasce994X	66994	JGI
	**Zasmidium citrigriseum**	Zasci044X	NHNW01000044.1	NCBI
		Zasci068X	NHNW01000068.1	NCBI
	**Zopfia rhizophila**	ZoprhX	741739	JGI
**Arthoniomycetes [XI]**	**Arthonia radiata**	ArtraXI	PSQN01000007.1	NCBI
**Lecanoromycetes [XII]**	**Agyrium rufum**	AgyruXII	JALDZB010000001.1	NCBI
	**Alectoria sarmentosa**	AlesaXII	WOGZ01000003.1	NCBI
	**Bacidia gigantensis**	BacgiXII	JAHXPR010000018.1	NCBI
	**Cladonia grayi**	ClagrXII	104134	JGI
	**Cladonia uncialis**	ClaunXII	NAPT01000294.1	NCBI
	**Evernia prunastri**	EveprXII	NKYR01000114.1	NCBI
	**Imshaugia aleurites**	ImsalXII	CAJPDT010000018.1	NCBI
	**Lasallia pustulata**	Laspu443XII	CALAON010000443.1	NCBI
		Laspu130XII	CALHDC010000130.1	NCBI
	**Letharia columbiana**	LetcoXII	JACCJC010000029.1	NCBI
	**Letharia lupina**	LetluXII	JACCJB010000008.1	NCBI
	**Letrouitia transgressa**	LettrXII	JALAIO010000034.1	NCBI
	**Lignoscripta atroalba**	LigatXII	JALDZG010000007.1	NCBI
	**Lobaria immixta**	LobimXII	659849	JGI
	**Lobaria pulmonaria Africa**	LobafXII	CE406415_2123	JGI
	**Lobaria pulmonaria Scotland**	LobscXII	2266336	JGI
	**Lobaria pulmonaria Spain**	LobspXII	391768	JGI
	**Loxospora ochrophaea**	LoxacXII	JALDZI010000011.1	NCBI
	**Mycoblastus sanguinarius**	MycsaXII	JALDZJ010000123.1	NCBI
	**Niebla homalea**	NiehoXII	JAHGAU010000002.1	NCBI
	**Parmelia sp.**	ParspXII	JAEUBB010000027.1	NCBI
	**Peltigera leucophlebia**	PelleXII	JALDZK010004437.1	NCBI
	**Physcia stellaris**	PhystXII	JABSSW010000035.1	NCBI
	**Pseudevernia furfuracea**	PsefuXII	NKYQ01000023.1	NCBI
	**Ptychographa xylographoides**	PtyxyXII	JALDZM010000094.1	NCBI
	**Puttea exsequens**	PutexXII	JALDZN010000015.1	NCBI
	**Ramalina farinacea**	RamfaXII	JAPUFD010000003.1	NCBI
	**Ramalina intermedia**	RaminXII	PEKF01000016.1	NCBI
	**Ramalina peruviana**	RampeXII	MSTJ01000221.1	NCBI
	**Thelotrema lepadinum**	TheleXII	JALDZR010000010.1	JGI
	**Toensbergia leucococca**	ToeleXII	JALDZS010000084.1	NCBI
	**Usnea florida**	UsnflXII	941042	JGI
	**Usnea hakonensis**	UsnhaXII	BLJB01000135.1	NCBI
	**Xylographa bjoerkii**	XylbjXII	JALDZV010000033.1	NCBI
	**Xylographa carneopallida**	XylcaXII	JALDZW010000016.1	NCBI
	**Xylographa pallens**	XylpalXII	JALDZY010000030.1	NCBI
	**Xylographa parallela**	XylparXII	JALDZZ010000074.1	NCBI
	**Xylographa soralifera**	XylsoXII	JALEAA010000004.1	NCBI
	**Xylographa trunciseda**	XyltrXII	JALEAB010000267.1	NCBI
	**Xylographa vitiligo**	XylviXII	JALEAC010000002.1	NCBI

	**Acephala macrosclerotiorum**	Acema745XIII	WHVH01000745.1	NCBI
	**Acephala macrosclerotiorum**	Acema591XIII	WHVH01000591.1	NCBI
	**Amylocarpus encephaloides**	AmyenXIII	JAFMPF010000503.1	NCBI
	**Arachnopeziza araneosa**	Araar078XIII	QYAI01000078.1	NCBI
		Araar106XIII	QYAI01000106.1	NCBI
	**Articulospora tetracladia**	ArtteXIII	PYIV01000003.1	NCBI
	**Ascocoryne sarcoides**	AscsaXIII	AIAA01000018.1	NCBI
	**Atropellis piniphila**	AtrpiXIII	491293	JGI
	**Bisporella sp.**	BisspXIII	JAJSPD010000046.1	NCBI
	**Botryotinia globosa**	BotglXIII	RCSZ01000006.1	NCBI
	**Botryotinia calthae**	BotcaXIII	PHWZ01000328.1	NCBI
	**Botryotinia convoluta**	BotcoXIII	PQXN01000239.1	NCBI
	**Botryotinia fuckeliana**	BotfuXIII	11934	JGI
	**Botryotinia narcissicola**	BotnaXIII	PQXJ01000088.1	NCBI
	**Botrytis aclada**	BotacXIII	RCSV01000007.1	NCBI
	**Botrytis byssoidea**	BotbyXIII	RCSW01000037.1	NCBI
	**Botrytis cinerea**	BotciXIII	9670	JGI
	**Botrytis elliptica**	BotelXIII	PQXM01000339.1	NCBI
	**Botrytis fabae**	BotfaXIII	RSAF01000543.1	NCBI
	**Botrytis galanthina**	BotgaXIII	PQXL01000369.1	NCBI
	**Botrytis hyacinthi**	BothyXIII	PQXK01000114.1	NCBI
	**Botrytis paeoniae**	BotpaXIII	LBGX01000679.1	NCBI
	**Botrytis porri**	BotpoXIII	PQXO01000220.1	NCBI
	**Botrytis pseudocinerea**	BotpsXIII	JAHXJK010000120.1	NCBI
	**Botrytis squamosa**	BotsqXIII	RCTC02000014.1	NCBI
	**Botrytis tulipae**	BottuXIII	PQXH01000026.1	NCBI
	**Cadophora sp**	Cadsp306XIII	CALMRP010000306.1	NCBI
		Cadsp232XIII	CALMRP010000232.1	NCBI
	**Cadophora malorum**	Cadma175XIII	JAFJYH010000175.1	NCBI
		Cadma255XIII	FKJQ01000255.1	NCBI
	**Cadophora luteo-olivacea**	CadloXIII	JALRMC010000003.1	NCBI
	**Cadophora gregata**	CadgrXIII	JASTTC010000012.1	NCBI
	**Cairneyella variabilis**	CaivaXIII	AYLM01000174.1	NCBI
	**Calycina herbarum**	CalheXIII	LLEY01000094.1	NCBI
	**Chaetoscypha palmicola**	ChapaXIII	QYAH01000124.1	JGI
	**Chalara longipes**	ChaloXIII	287508	JGI
	**Chlorencoelia torta**	ChltoXIII	QYAN01000006.1	NCBI
	**Chlorociboria aeruginascens**	ChlaeXIII	NCSK02000007.1	NCBI
	**Ciborinia camelliae**	CibcaXIII	LGKQ01000062.1	NCBI
	**Ciborinia shiraiana**	CibshXIII	VNFM01000008.1	NCBI
	**Clarireedia aff_paspali**	ClaafXIII	JAJCXA010000069.1	NCBI
	**Clarireedia hainanense**	ClahaXIII	JANHAA010000108.1	NCBI
	**Clarireedia homoeocarpa**	Claho107XIII	JANHAB010000107.1	NCBI
	**Clarireedia homoeocarpa**	Claho380XIII	LLKD01000380.1	NCBI
	**Clarireedia jacksonii**	ClajaXIII	JANHAG010000054.1	NCBI
	**Clarireedia monteithiana**	ClamoXIII	JANHAD010000068.1	NCBI
	**Clarireedia paspali**	ClapaXIII	JANCMV010000057.1	NCBI
	**Coleophoma crateriformis**	ColcrXIII	PDLN01000002.1	NCBI
	**Coleophoma cylindrospora**	ColcyXIII	PDLN01000002.1	NCBI
	**Coleophoma eucalyptorum**	Coleu023XIII	JALRME010000023.1	NCBI
		Coleu018XIII	JALRME010000018.1	NCBI
	**Cudoniella acicularis**	CudacXIII	JAAMPI010000570.1	NCBI
	**Filosporella fistucella**	FilfiXIII	JAQJZZ010000021.1	NCBI
	**Fungal_sp._EF0021**	FunspXIII	AIET01001736.1	NCBI
	**Gamarada debralockiae**	GamdeXIII	NXFV01001982.1	NCBI
	**Glarea lozoyensis**	GlaloXIII	XM_008089548.1	NCBI
	**Halenospora varia**	HalvaXIII	JAHEWH010000025.1	NCBI
	**Haplographium delicatum**	HapdeXIII	JAPZPC010000048.1	NCBI
	**Helotiales sp. F229**	Helsp003XIII	MXAW01000003.1	NCBI
	**Helotiales spP8P42**	Helsp746XIII	765746	JGI
	**Helotiales spP8C63**	Helsp679XIII	580679	JGI
	**Hyaloscypha bicolor**	Hyabi801XIII	XM_024874801.1	NCBI
		Hyabi107XIII	LXPI01000107.1	NCBI
	**Hyaloscypha finlandica**	Hyafi048XIII	1200048	NCBI
		Hyafi065XIII	JAJTUR010000065.1	NCBI
	**Hyaloscypha hepaticicola**	Hyahe191XIII	LYBP01000191.1	NCBI
		Hyahe659XIII	LYBP01000659.1	NCBI
	**Hyaloscypha variabilis**	Hyava031XIII	LXPR01000031.1	NCBI
		Hyava165XIII	LXPR01000165.1	NCBI
	**Hyaloscypha sp.**	Hyasp023XIII	JAJTUS010000023.1	NCBI
		Hyasp114XIII	JAJTUS010000114.1	NCBI
	**Hymenoscyphus albidus**	HymalXIII	CAJVRM010000262.1	NCBI
	**Hymenoscyphus fructigenus**	HymfrXIII	LKUV01000221.1	NCBI
**Leotiomycetes [XIII]**	**Hymenoscyphus herbarum**	HymheXIII	LLEY01000094.1	NCBI
	**Hymenoscyphus koreanus**	HymkoXIII	CACRVL010000089.1	NCBI
	**Hymenoscyphus linearis**	HymliXIII	UNPR01000151.1	NCBI
	**Hymenoscyphus occultus**	HymocXIII	UNPT01000089.1	NCBI
	**Hymenoscyphus repandus**	HymreXIII	LLCE01000084.1	NCBI
	**Hymenoscyphus scutula**	HymscXIII	LKTO01000301.1	NCBI

**Hymenoscyphus varicosporoides**	HymvaXIII	389547	JGI
**Hyphodiscus hymeniophilus**	Hyphy004XIII	VNKQ01000004.1	NCBI
	Hyphy020XIII	VNKQ010000020.1	NCBI
**Hyphodiscus sp.**	Hypsp019XIII	QYAO0100002.1	NCBI
	Hypsp002XIII	QYAO01000019.1	NCBI
**Lachnellula suecica**	LacsuXIII	QGMK01000177.1	NCBI
**Lachnum nothofagi**	LacnoXII	QYSG01014932.1	NCBI
**Lanzia echinophila**	LanecXIII	JWJA01005407.1	NCBI
**Leotiomycetes sp.**	Leosp001XIII	JAGMVK010000001.1	NCBI
	Leosp022XIII	JAGMVK010000022.1	NCBI
**Leptodontidium sp.**	LepspXIII	675498	NCBI
**Leptodontidium orchidicola**	LeporXIII	552792	JGI
**Meliniomyces bicolor**	MelbiXIII	589832	JGI
**Meliniomyces variabilis**	MelvaXIII	562787	JGI
**Meliniomyces sp**	MelspXIII	352757	JGI
**Mollisia scopiformis**	MolscXIII	LKNI01000172.1	NCBI
**Monilinia fructigena**	Monfr031XIII	QKRW01000031.1	NCBI
**Monilinia fructicola**	Monfr156XIII	PDME01000156.1	NCBI
**Monilinia laxa**	MonlaXIII	PDMC01000076.1	NCBI
**Monilinia polystroma**	MonpoXIII	PDUE01000182.1	NCBI
**Myriosclerotinia duriaeana**	MyrduXIII	NGKI01000003.1	NCBI
**Myriosclerotinia scirpicola**	MyrscXIII	NGKG01000023.1	NCBI
**Neobulgaria alba**	NeoalXIII	QYAM01000268.1	NCBI
**Neophaeococcomyces sp.**	NeospXIII	gw1.11.369.1	JGI
**Oculimacula yallundae**	OcuyaXIII	12964	JGI
**Oidiodendron maius**	Oidma909XIII	115909	JGI
	Oidma761XIII	25761	JGI
**Pasadenomyces melaninifex**	PasmeXIII	JAKLMI010000083.1	NCBI
**Pezicula neosporulosa**	PezneXIII	SELE01000043.1	NCBI
**Pezicula radicicola**	PezraXIII	PDUO01000008.1	NCBI
**Pezoloma ericae**	Pezer191XIII	LYBP01000191.1	NCBI
	Pezer659XIII	LYBP01000659.1	NCBI
**Phialocephala scopiformis**	PhiscXIII	Genemark1.C_200373	JGI
**Phialocephala subalpina**	Phisu016XIII	FJOG01000016.1	NCBI
	Phisu005XIII	FJOG01000005.1	NCBI
**Phialocephala sp.**	Phisp275XIII	QYAJ01000275.1	NCBI
	Phisp052XIII	QYAJ01000052.1	NCBI
**Pirottaea palmicola**	PirpaXIII	QYAH01000124.1	NCBI
**Polyphilus sieberi**	PolsiXIII	821700	JGI
**Proliferodiscus dingleyae**	ProdiXIII	QYAK01000005.1	NCBI
**Pseudogymnoascus pannorum**	Psepa040XIII	JPJV01000040.1	NCBI
	Psepa427XIII	JPKC01002427.1	NCBI
	Psepa998XIII	JPKC01000998.1	NCBI
	Psepa562XIII	JPKC01002562.1	NCBI
**Pseudogymnoascus verrucosus**	PseveXIII	XM_018273049.1	NCBI
**Pseudogymnoascus sp.**	Psesp419XIII	LAJK01001419.1	NCBI
	Psesp215XIII	LNAR01000215.1	NCBI
**Rhexocercosporidium sp.**	RhespXIII	CE243899_979	NCBI
**Rhizoscyphus ericae**	RhierXIII	140108	JGI
**Rhynchosporium agropyri**	RhyagXIII	FJUX01000123.1	NCBI
**Rhynchosporium commune**	RhycoXIII	FJUW01000001.1	NCBI
**Rhynchosporium secalis**	RhyseXIII	FJVC01000033.1	NCBI
**Rutstroemia firma**	RutfiXIII	ıesh1_kg.1_#_44_#_Locus3398v1rpkm3!	JGI
**Rutstroemia sp.**	Rutsp067XIII	NJPS01000067.1	NCBI
	Rutsp092XIII	NJPT01000092.1	NCBI
	Rutsp083XIII	NJPT01000083.1	NCBI
**Sclerotinia glacialis**	ScglXIII	NGKH01000007.1	NCBI
**Sclerotinia homoeocarpa**	SclhoXIII	JW827172.1	NCBI
**Sclerotinia pseudotuberosa**	SclpsXIII	JAWPPJ010000184.1	NCBI
**Sclerotinia sclerotiorum**	SclscXIII	Supercontig_2.6:161229-162757	JGI
**Scytalidium lignicola**	ScyliXIII	NCSJ01000117.1	NCBI
**Scytalidium sp.**	Scysp010XIII	JMRO03000010.1	NCBI
	Scysp005XIII	JMRO03000005.1	NCBI
**Stipitochalara longipes**	Stilo042XIII	VKGA01000042.1	NCBI
	Stilo135XIII	VKGA01000135.1	NCBI
**Xylogone sp**	Xylsp001XIII	JAJTUQ010000001.1	NCBI
	Xylsp003XIII	JAJTUQ010000003.1	NCBI
**Zalerion varium**	ZalvaXIII	659357	JGI

**Acidothrix acidophila**	AciacXIV	1468444	JGI
**Acremonium furcatum**	AcrfuXIV	BCIA01000001.1	NCBI
**Acremonium strictum**	AcrstXIV	433516	JGI
**Albifimbria verrucaria**	AlbverXIV	JAHREC010000049.1	NCBI
**Albophoma yamanashiensis**	AlbyaXIV	BCKH01000003.1	NCBI
**Apiospora malaysiana**	ApimaXIV	QUSE01000011.1	NCBI
**Apiospora montagnei**	Apimo70656XIV	70656	JGI
	Apimo33434XIV	33434	JGI
**Apiospora saccharicola**	ApisaXIV	JAFHKF010000002.1	NCBI
**Apodospora peruviana**	ApopeXIV	615849	JGI
**Aquanectria penicillioides**	AqupeXIV	PYIU01000007.1	NCBI
**Arthrinium esporlense**	ArtesXIV	346068	JGI
**Arthrinium malaysianum**	Artma516XIV	GEGW01002516.1	NCBI
**Arthrinium malaysianum**	Artma031XIV	QUSE01000031.1	NCBI
**Arthrinium phaeospermum**	ArtphXIV	GHWG01004007.1	NCBI
**Arthrinium sp**	ArtspXIV	JAFHKQ010000014.1	NCBI
**Aurifilum marmelostoma**	AurmaXIV	JAMXSV010000003.1	NCBI
**Beauveria bassiana**	BeabaXIV	ADAH01000691.1	NCBI
**Beauveria brongniartii**	BeabrXIV	AZHA01000037.1	NCBI
**Beauveria pseudobassiana**	BeapsXIV	LUDX01000493.1	NCBI
**Beauveria rudraprayagi**	BearuXIV	JMNB01004563.1	NCBI
**Beauveria sp.**	BeaspXIV	MSJG02000017.1	NCBI
**Calcarisporium arbuscula**	CalarXIV	WBSA01000013.1	NCBI
**Calonectria henricotiae**	CalheXIV	PHMY01017932.1	NCBI
**Calonectria leucothoes**	CalleXIV	NAJI01000079.1	NCBI
**Calonectria naviculata**	CalnaXIV	NAGG01000382.2	NCBI
**Calonectria pseudonaviculata**	Calps272XIV	JYJY01000272.1	NCBI
**Calonectria pseudoreteaudii**	Calps560XIV	MOCD01000560.1	NCBI
**Cephalotrichum gorgonifer**	Cepgo009XIV	ONZQ02000001.1	NCBI
	Cepgo001XIV	ONZQ02000009.1	NCBI
**Cercophora caudata**	CercaXIV	460915	JGI
**Cercophora newfieldiana**	CerneXIV	391490	JGI
**Cercophora scortea**	CerscXIV	186623	JGI
**Chaetomidium fimeti**	ChafiXIV	473356	JGI
**Chaetomium funicola**	ChafuXIV	150049	JGI
**Chaetosphaeriaceae sp.**	ChaspXIV	679560	JGI
**Chaetosphaeria innumera**	ChainXIV	3461	JGI
**Chrysoporthe austroafricana**	ChrauXIV	JYIP01000729.1	NCBI
**Chrysoporthe cubensis**	ChrcuXIV	LJCY01000190.1	NCBI
**Chrysoporthe deuterocubensis**	ChrdeXIV	LJDD01000019.1	NCBI
**Chrysoporthe zambiensis**	ChrzaXIV	JASENA010000039.1	NCBI
**Cladobotryum protrusum**	ClaprXIV	RZGP01000007.1	NCBI
**Clonostachys sp.**	ClospXIV	404401	JGI
**Clonostachys chloroleuca**	ClochXIV	JBANAY010000003.1	NCBI
**Clonostachys rhizophaga**	ClorhXIV	JBICSC010000042.1	NCBI
**Clonostachys rosea**	CloroXIV	LWRQ01000169.1	NCBI
**Coniochaeta ligniaria**	ConliXIV	643574	JGI
**Coniochaeta sp.**	Consp056XIV	155056	JGI
	Consp149XIV	967149	JGI
**Cordyceps militaris**	CormiXIV	AEVU01000101.1	NCBI
**Cordyceps pruinosa**	CorprXIV	LUFD01003780.1	NCBI
**Cordyceps sp.**	CorspXIV	NJEV01000632.1	NCBI
**Corinectria fuckeliana**	CorfuXIV	QGQC01000039.1	NCBI
**Corynascella inaequalis**	CorinXIV	11861	JGI
**Cryphonectria carpinicola**	CrycaXIV	JACWRE010000115.1	NCBI
**Cryphonectria naterciae**	CrynaXIV	JACWSS010000265.1	NCBI
**Cryphonectria radicalis**	CryraXIV	NAQO01000457	NCBI
**Cylindrocarpon olidum**	CylolXIV	405339	JGI
**Dactylonectria estremocensis**	DacesXIV	270019	JGI
**Dactylonectria macrodidyma**	DacmaXIV	JYGD01000417.1	NCBI
**Diaporthe longicolla**	DialoXIV	AYRD01007349.1	NCBI
**Diaporthe ampelina**	DiaamXIV	LWAD01000237.1	NCBI
**Diaporthe vochysiae**	DiavoXIV	492051	JGI
**Diatrype disciformis**	DiadiXIV	807117	JGI
**Diatrype stigma**	DiastXIV	JAKJXP010000050.1	NCBI
**Echria macrotheca**	EchmaXIV	JAUFNW010000089.1	NCBI
**Endothia cerciana**	EndceXIV	JALRMH010000049.1	NCBI
**Escovopsis sp.**	EscspXIV	NIGD01000012.1	NCBI
**Escovopsis weberi**	EscweXIV	NIGB01000010.1	NCBI
**Eutypa lata**	EutlaXIV	AORF01002616.1	NCBI
**Falciphora oryzae**	FalorXIV	JNVV01000009.1	NCBI
**Fragosphaeria purpurea**	FrapuXIV	PCDL01000002.1	NCBI
**Fungal_sp.**	FunspXIV	PNEP01000019.1	NCBI
**Furcasterigmium furcatum**	FurfuXIV	BCIA01000001.1	NCBI
**Fusarium acuminatum**	FusacXIV	CBMG010003518.1	NCBI
**Fusarium agapanthi**	FusagXIV	LUFC01000741.1	NCBI
**Fusarium algeriense**	FusalXIV	PVPY01000620.1	NCBI
**Fusarium ambrosium**	FusamXIV	NIZV01000012.1	NCBI
**Fusarium asiaticum**	FusasXIV	LHTZ01000061.1	NCBI

	**Fusarium azukicola**	FusazXIV	MAEG01007923.1	NCBI
	**Fusarium beomiforme**	FusbeXIV	PVQB01000488.1	NCBI
	**Fusarium brasiliense**	FusbrXIV	MAEC01005884.1	NCBI
	**Fusarium circinata**	FusciXIV	AYJV01002164.1	NCBI
	**Fusarium commune**	FuscoXIV	MIX3296_8_63	JGI
	**Fusarium culmorum**	FusculXIV	CBMH010000861.1	NCBI
	**Fusarium cuneirostrum**	FuscunXIV	MAEA01002323.1	NCBI
	**Fusarium equiseti**	FuseqXIV	CBMI010004636.1	NCBI
	**Fusarium euwallaceae**	FuseuXIV	NHTE02000046.1	NCBI
	**Fusarium fujikuroi**	FusfuXIV	ANFV01000069.1	NCBI
	**Fusarium fracticaudum**	FusfrXIV	PDNT01000011.1	NCBI
	**Fusarium graminearum**	FusgrXIV	HG970333	NCBI
	**Fusarium mangiferae**	FusmaXIV	FCQH01000014.1	NCBI
	**Fusarium meridionale**	FusmeXIV	LHUB01000087.1	NCBI
	**Fusarium nygamai**	FusnyXIV	LBNR01000016.1	NCBI
	**Fusarium oxysporum Lycopersici**	FusolXIV	AAXH01000716	NCBI
	**Fusarium oxysporum Raphani**	FusorXIV	mrna_FOQG_11832T0	NCBI
	**Fusarium oxysporum f. sp. Vasinfectum**	FusovXIV	AGNC01000296.1	NCBI
	**Fusarium phaseoli**	FusphXIV	MAEB01000885.1	NCBI
	**Fusarium pininemorale**	FuspiXIV	NFZR01000029.1	NCBI
	**Fusarium pseudograminearum**	FuspsXIV	EKJ77666	NCBI
	**Fusarium temperatum**	FusteXIV	LJGR01000010.1	NCBI
	**Fusarium verticillioides**	FusveXIV	AAIM02000133.1	NCBI
	**Fusarium udum**	FusudXIV	NIFK01000011.1	NCBI
	**Fusarium virguliforme**	FusviXIV	AEYB01001170.1	NCBI
	**Gaeumannomyces graminis var. tritici**	GaegrXIV	GGTG_06660	JGI
	**Gibberella moniliformis**	GibmoXIV	AAIM02000133	NCBI
	**Gibberella zeae**	GibzeXIV	NT_086532.1	NCBI
	**Gliomastix tumulicola**	GlostXIV	BCHX01000001.1	NCBI
	**Graphilbum fragrans**	GrafrXIV	LLKO01000009.1	NCBI
	**Grosmannia galeiformis**	GrogaXIV	RQWE01000006.1	NCBI
	**Harpophora oryzae**	HarorXIV	JNVV01000009.1	NCBI
	**Hawksworthiomyces Lignivorus**	HawigXIV	NTMA01000093.1	NCBI
	**Hirsutella minnesotensis**	HirmiXIV	JPUM01001078.1	NCBI
	**Hirsutella rhossiliensis**	HirrhXIV	MPJM01000322.1	NCBI
	**Hypocreaceae sp.**	Hypsp126XIV	DAGWQZ010000126.1	NCBI
	**Hypomyces perniciosus**	HyppeXIV	SPDT01000008.1	NCBI
	**Hypomyces rosellus**	HyproXIV	WWCI01000001.1	NCBI
	**Ilyonectria sp**	IlyspXIV	531675	JGI
	**Ilyonectria destructans**	IlydeXIV	MPHF01000127.1	NCBI
	**Ilyonectria europaea**	IlyeuXIV	1809843	JGI
	**Ilyonectria mors-panacis**	IlymoXIV	PPHJ01000084.1	NCBI
	**Ilyonectria robusta**	IlyroXIV	59233	JGI
	**Keithomyces neogunnii**	KeineXIV	JAIOWQ010000006.1	JGI
	**Khuskia oryzae**	KhuorXIV	485486	JGI
	**Knoxdaviesia capensis**	KnocaXIV	LNGK01000008.1	NCBI
	**Knoxdaviesia proteae**	KnoprXIV	LNGK01000008.1	NCBI
	**Lasiosphaeria ovinas**	LasovXIV	637879	JGI
	**Lasiosphaeris hirsuta**	LashirXIV	94943	JGI
	**Lasiosphaeris hispida**	LashisXIV	480539	JGI
	**Lasiosphaeriaceae sp.**	LasspXIV	573813	JGI
	**Lecanicillium fungicola**	LecfuXIV	FWCC01000362.1	NCBI
	**Lecanicillium psalliotae**	LecpsXIV	PHFE01000067.1	NCBI
	**Leptographium procerum**	LepprXIV	JRUC01000485.1	NCBI
	**Lomentospora prolificans**	LomprXIV	NLAX01001139.1	NCBI
	**Magnaporthiopsis incrustans**	MaginXIV	PYSR01000005.1	NCBI
**Sordariomycetes [XIV]**	**Magnaporthiopsis poae**	MagpoXIV	MAPG_11684T0	NCBI
	**Magnaporthiopsis rhizophila**	MagrhXIV	PYSS01000229.1	NCBI
	**Mariannaea sp.**	MarspXIV	514108	JGI
	**Melanconium sp.**	Melsp003XIV	360003	JGI
	**Metacordyceps chlamydosporia**	MetchXIV	AOSW01005593.1	JGI
	**Metarhizium acridum**	MetacXIV	EFY92789	NCBI
	**Metarhizium album**	MetalXIV	AZHE01000016.1	NCBI
	**Metarhizium anisopliae**	MetanXIV	APNB01000197.1	NCBI
	**Metarhizium brunneum**	MetbrXIV	AZNG01000001.1	NCBI
	**Metarhizium guizhouense**	MetguXIV	AZNH01000004.1	NCBI
	**Metarhizium majus**	MetmaXIV	AZNE01000014.1	NCBI
	**Metarhizium robertsii**	MetroXIV	MAA_05705T0	JGI
	**Microascus cirrosus**	Micci310XIV	JAMBUN010000310.1	NCBI
		Micci062XIV	JAMBUN010000062.1	NCBI
	**Microascus trigonosporus**	Mictr040XIV	549040	JGI
		Mictr983XIV	92983	JGI
	**Microcera coccophila**	MiccoXIV	JAMYIC010000931.1	NCBI
	**Microdochium trichocladiopsis**	Mictr772XIV	291772	NCBI
	**Monosporascus sp.**	MonspXIV	QJNV01000907.1	NCBI
	**Monosporascus cannonballus**	MoncaXIV	QJNT01000265.1	NCBI
	**Monosporascus ibericus**	MonibXIV	QJNU01000022.1	NCBI
	**Mycogone perniciosa**	MycpeXIV	JAOALB010000107.1	NCBI
	**Myrothecium inundatum**	MyrinXIV	269554	JGI

**Nakataea oryzae**	NakorXIV	PYSQ01000639.1	JGI
**Nectria haematococca**	NechaXIV	XP_003042844.1	NCBI
**Nectria sp.**	NecspXIV	NOKP01000031.1	NCBI
**Neocosmospora boninensis**	NeoboXIV	SSHR01000129.1	JGI
**Neonectria ditissima**	NeodiXIV	3637	NCBI
**Neonectria hederae**	NeoheXIV	QGQB01000097.1	NCBI
**Neonectria punicea**	NeopuXIV	QGQA01000657.1	NCBI
**Neonectria sp.**	NeospXIV	RQWH01000022.1	NCBI
**Neopestalotiopsis sp.**	NeopspXIV	601256	JGI
**Niesslia exilis**	NieexXIV	220466	JGI
**Ophiocordycipitaceae sp.**	OphspXIV	DAGXBU010001383.1	NCBI
**Parascedosporium putredinis**	ParpuXIV	CASHTG010000006.1	NCBI
**Paramyrothecium roridum**	ParroXIV	PXOD01000275.1	NCBI
**Paramyrothecium sp**	ParspXIV	JAJFPB010000004.1	NCBI
**Parapyrenis maritima**	ParmaXIV	127418	NCBI
**Parathielavia appendiculata**	ParapXIV	565516	NCBI
**Parathielavia hyrcaniae**	ParhyXIV	516839	NCBI
**Penicillatus**	PenspXIV	VCCP01000005.1	NCBI
**Phaeoacremonium aleophilum**	PhaalXIV	8398m.01	JGI
**Phaeoacremonium minimum**	PhamiXIV	XM_007920911.1	NCBI
**Phaeoacremonium sp.**	PhaspXIV	gm1.6071_g	JGI
**Phialemoniopsis curvata**	PhicuXIV	SKBQ01000027.1	NCBI
**Phialemonium atrogriseum**	PhiatXIV	547419	NCBI
**Phialemonium globosum**	PhiglXIV	429001	NCBI
**Plectosphaerella cucumerina**	PlecuXIV	JAGPXD010000005.1	NCBI
**Plectosphaerella plurivora**	PleplXIV	JAGSXJ010000025.1	NCBI
**Plectosphaerella sp.**	PlespXIV	JACAFV010000166.1	NCBI
**Pochonia chlamydosporia**	Pocch001XIV	AOSW01003564.1	NCBI
	Pocch564XIV	AOSW04000001.1	JGI
**Podospora aff communis**	PodcoXIV	452915	JGI
**Podospora curvicolla**	PodcuXIV	351100	JGI
**Podospora decipiens**	PoddeXIV	434686	JGI
**Purpureocillium lavendulum**	PurlaXIV	JAQHRD010000001.1	NCBI
**Purpureocillium lilacinum**	PurliXIV	LCWV01000016.1	NCBI
**Purpureocillium takamizusanense**	PurtaXIV	XP_047837310.1	NCBI
**Purpureocillium sp.**	PurspXIV	347422	JGI
**Reticulascus tulasneorum**	RettuXIV	LSAX01000001.1	NCBI
**Sarocladium kiliense**	SarkiXIV	JAUZTG010000041.1	NCBI
**Sarocladium strictum**	SarstXIV	OKRM01000006.1	NCBI
**Sarocladium terricola**	SarteXIV	JAHKSO010000001.1	NCBI
**Scedosporium aurantiacum**	SceauXIV	JUDQ01000065.1	NCBI
**Scedosporium apiospermum**	SceapXIV	JOWA01000089.1	NCBI
**Scedosporium dehoogii**	ScedeXIV	PGIR01000082.1	NCBI
**Schizothecium conicum**	SchcoXIV	JAUFRE010000017.1	NCBI
**Schizothecium tetrasporum**	SchteXIV	JAQKAE010000004.1	NCBI
**Schizothecium vesticola**	SchveXIV	gw1.6.2428.1	JGI
**Simplicillium aogashimaense**	SimaoXIV	JAKHSJ010000006.1	NCBI
**Simplicillium sp.**	SimspXIV	JAJIYY010000007.1	NCBI
**Sordariomycetes sp.**	Sorsp039XIV	JAMFQS010000039.1	NCBI
**Sordariomycetes sp.**	Sorsp089XIV	JAMFQS010000089.1	NCBI
**Sphaerostilbella broomeana**	SphbrXIV	CAKNET010000059.1	NCBI
**Sporothrix dimorphospora**	SpodiXIV	WOUA01000023.1	NCBI
**Sporothrix insectorum**	SpoinXIV	AZHD01000005.1	NCBI
**Sporothrix schenckii**	SposcXIV	AWEQ01000100.1	NCBI
**Stachybotrys chartarum**	StachXIV	APIU01000295.1	NCBI
**Stachybotrys chlorohalonata**	Stachl470XIV	APWP01002470.1	NCBI
	Stachl612XIV	APWP01003612.1	NCBI
**Stachybotrys echinata**	StaecXIV	BCHF01000013.1	NCBI
**Stachybotrys elegans**	StaelXIV	MIX15202_195_47	NCBI
**Stachybotrys microspora**	StamiXIV	JAEVLY010002147.1	NCBI
**Stanjemonium grisellum**	StagrXIV	e_gw1.4.163.1	JGI
**Staphylotrichum longicolle**	StaloXIV	19970	JGI
**Stenocarpella maydis**	StemaXIV	NQMI01000013.1	NCBI
**Thielavia appendiculata**	ThiapXIV	Genemark1.C_2_t30079	JGI
**Thielavia hyrcaniae**	ThihyXIV	fgenesh1_pm.C_200029	JGI
**Thozetella sp.**	ThospXIV	734478	JGI
**Thyridium sp.**	ThyspXIV	561948	JGI
**Togninia minima**	TogmiXIV	XM_007920911.1	NCBI
**Tolypocladium album**	TolalXIV	JALHCH010000467.1	NCBI
**Tolypocladium amazonense**	TolamXIV	JALHCH010000467.1	NCBI
**Tolypocladium capitatum**	TolcaXIV	NRSZ01000447.1	NCBI
**Tolypocladium cylindrosporum**	TolcyXIV	JALHCC010000467.1	NCBI
**Tolypocladium endophyticum**	TolenXIV	JALHBW010000176.1	NCBI
**Tolypocladium geodes**	TolgeXIV	JALHCB010000069.1	NCBI
**Tolypocladium guangdongense**	TolguXIV	NRQP01000004.1	NCBI
**Tolypocladium inflatum**	TolinXIV	AOHE01000147.1	NCBI
**Tolypocladium ophioglossoides**	TolopXIV	LFRF01000016.1	NCBI
**Tolypocladium pustulatum**	TolpuXIV	JALHBY010000071.1	NCBI
**Tolypocladium salcha**	TolsaXIV	JPIJ02000005.1	NCBI

	**Tolypocladium sp**	TolspXIV	JPHH01000339.1	NCBI
	**Tolypocladium tropicale**	ToltrXIV	JALHBU010000100.1	NCBI
	**Torrubiella hemipterigena**	TorheXIV	CDHN01000004.1	NCBI
	**Trichoderma afroharzianum**	TriafXIV	JALIRT010000001.1	NCBI
	**Trichoderma arundinaceum**	TriarXIV	PXOA01000064.1	NCBI
	**Trichoderma asperellum**	TriasXIV	fgenesh1_pm.2_#_21	JGI
	**Trichoderma atrobrunneum**	Triat015XIV	PNRQ01000015.1	NCBI
	**Trichoderma atroviride**	Triat243XIV	319992	JGI
	**Trichoderma breve**	Tribr001XIV	JAOPEN010000001.1	NCBI
	**Trichoderma brevicompactum**	Tribr573XIV	PXNZ01000573.1	NCBI
	**Trichoderma citrinoviride**	TriciXIV	gm1.72_g	JGI
	**Trichoderma cornu-damae**	TricoXIV	JAIWOZ010000003.1	NCBI
	**Trichoderma erinaceum**	TrierXIV	JABSTY010000006.1	NCBI
	**Trichoderma gamsi**	TrigaXIV	JPDN01000001.1	NCBI
	**Trichoderma gracile**	TrigrXIV	JAINEY010000002.1	NCBI
	**Trichoderma guizhouense**	TriguXIV	LVVK01000020.1	NCBI
	**Trichoderma hamatum**	TrihamXIV	ANCB02000213.1	NCBI
	**Trichoderma harzianum**	TriharXIV	fgenesh1_pm.1_#_258	JGI
	**Trichoderma koningii**	Triko003XIV	BCGH01000003.1	NCBI
	**Trichoderma koningiopsis**	Triko140XIV	MRBD01000140.1	NCBI
	**Trichoderma lixii**	TriliXIV	SESN01000071.1	JGI
	**Trichoderma longibrachiatum**	TriloXIV	CE139160_1188	JGI
	**Trichoderma parareesei**	TripaXIV	LFMI01000206.1	NCBI
	**Trichoderma pleuroti**	TriplXIV	MDJU01000245.1	NCBI
	**Trichoderma polysporum**	TripoXIV	JARFTM010000123.1	JGI
	**Trichoderma reesei**	TrireXIV	fgenesh5_pg.C_scaffold_6000096	JGI
	**Trichoderma semiorbis**	TriseXIV	JAIMJC010000001.1	JGI
	**Trichoderma sp**	TrispXIV	JAJNCI010000002.1	JGI
	**Trichoderma virens**	Trivire129XIV	222129	JGI
		Trivire919XIV	194919	JGI
	**Trichoderma viride**	TriviriXIV	VCEC01000014.1	JGI
	**Truncatella angustata**	TruanXIV	508630	JGI
	**Vermiculariopsiella pediculata**	VerpeXIV	475567	JGI
	**Xylariaceae sp.**	Xylsp373XIV	639373	JGI
	**Xylariales sp.**	Xylsp638XIV	217638	JGI
**Xylonomycetes [XV]**	**Symbiotaphrina buchneri**	SymbuXV	BCIG01000027.1	NCBI
	**Symbiotaphrina kochii**	SymkoXV	esh1_kg.1_#_5885_#_Locus6071v1rpkm	JGI
	**Trinosporium guianense**	TriguXV	CE48284_233	JGI
	**Xylona heveae**	Xylhe044XV	JXCS01000044.1	JGI
		Xylhe054XV	JXCS01000054.1	JGI
	**Xylonomycetes sp.**	Xylsp016XV	JADCQY010000016.1	JGI
**Saccharomycota [XVI]**	**Debaryomycetaceae sp.**	DebspXVI	JAMAFS010000025.1	NCBI
	**Galactomyces candidus**	GalcaXVI	JMRO02000005.1	NCBI
	**Lipomyces arxii**	LiparXVI	PPJT02000021.1	NCBI
	**Lipomyces doorenjongii**	LipdoXVI	412976	JGI
	**Lipomyces kononenkoae**	LipkoXVI	PPJW01000018.1	NCBI
	**Lipomyces oligophaga**	LipolXVI	PPJR02000001.1	NCBI
	**Lipomyces orientalis**	LiporXVI	342385	JGI
	**Lipomyces spencermartinsiae**	LipspXVI	JAJLTQ010000017.1	JGI
	**Lipomyces sp.**	LipsXVI	PPJW02000018.1	JGI
	**Lipomyces starkeyi**	LipstXVI	191638	JGI
	**Lipomyces tetrasporus**	LipteXVI	starkeyi	JGI
	**Lipomyces yarrowii**	LipyaXVI	JANJPG010000190.1	NCBI
	**Spencermartinsiella europaea**	SpeeuXVI	PPIE02000004.1	JGI
**Glomeromycotina [XVIII]**	**Ambispora leptoticha**	Amble823XVIII	CAJVPS010000823.1	NCBI
		Amble163XVIII	CAJVPS010004163.1	NCBI
	**Geosiphon pyriformis**	Geopy171XVIII	9171	JGI
		Geopy098XVIII	14098	JGI
**Zoopagomycotina [XXI]**	**Acaulopage tetraceros**	AcateXXI	QZWU01000072.1	NCBI
**Entomophthoromycotina [XXII]**	**Basidiobolus heterosporus**	Bashe894XXII	JNET01031894.1	NCBI
		Bashe773XXII	JNET01012773.1	NCBI
	**Basidiobolus meristosporus**	Basme033XXII	3790033	JGI
		Basme565XXII	JNEO01006565.1	NCBI
	**Basidiobolus ranarum**	Basra596XXII	JASJQH010002596.1	NCBI
		Basra953XXII	JASJQH010008953.1	NCBI
