# Supplementary material for: Major Intrinsic Proteins in Fungi: A Special Emphasis on the XIP Subfamily
Source: J Fungi (Basel). 2025 Jul 21;11(7):543. doi: 10.3390/jof11070543 (PMC12300952; doi:10.3390/jof11070543)
Supplement: Supplementary file 1 [file jof-11-00543-s001.zip › jof-3752183_Supplementary_Table_S2.pdf]

Supplementary Table S2. full-length aquaporin protein sequences from one species of each major fungal phylum detailed on JGI's MycoCosm portal.

XIP sequences are highlighted in red.

Code	Phyla	Species	JGI nomenclature	Complete protein sequence
I	Pucciniomycotina	*Cronartium quercuum*	Croqu35404	[illegible]
			Croqu68699	[illegible]
			Croqu659961	[illegible]
			Croqu49229	[illegible]
			Croqu652096	[illegible]
			Croqu656049	[illegible]
			Croqu37757	[illegible]
			Croqu284390	[illegible]
			Croqu75289	[illegible]
			Croqu661726	[illegible]
			Croqu845I	[illegible]
			Croqu6585I	[illegible]
II	Ustilaginomycotina	*Ustilago maydis*	Ustma10398	[illegible]
			Ustma8828	[illegible]
			Ustma8384	[illegible]
			Ustma9367	[illegible]
			Ustma7026	[illegible]
			Ustma10090	[illegible]
III	Agaricomycetes	*Mycena alexandri*	Mycal1036362	[illegible]
			Mycal1115705	[illegible]
			Mycal1210769	[illegible]
			Mycal1212415	[illegible]
			Mycal262410	[illegible]
			Mycal1003170	[illegible]
			Mycal1052167	[illegible]
			Mycal1113161	[illegible]
			Mycal1355211	[illegible]
			Mycal241214	[illegible]
			Mycal1085978	[illegible]
			Mycal1170102	[illegible]
IV	Dacrymycetes	*Dacryonaema rufum*	Dacru2265047	[illegible]
			Dacru2260629	[illegible]
			Dacru1262245	[illegible]
			Dacru2357520	[illegible]
			Dacru519289	[illegible]
			Dacru1357811	[illegible]
			Dacru2252672	[illegible]
			Dacru2256153	[illegible]
			Dacru2276186	[illegible]
			Dacru202916	[illegible]
			Dacru2420693	[illegible]
V	Tremellomycetes	*Fellomyces penicillatus*	Felpe250787	[illegible]
			Felpe250799	[illegible]
			Felpe261217	[illegible]
			Felpe257230	[illegible]
			Felpe277600	[illegible]
VI	Wallemiomycetes	*Wallemia mellicola*	Walme31249	[illegible]
			Walme32912	[illegible]
			Walme59835	[illegible]
VII	Pezizomycetes	*Gyromitra esculenta*	Gyres619320	[illegible]
			Gyres459760	[illegible]
			Gyres550632	[illegible]
			Gyres517966	[illegible]
VIII	Orbiliomycetes	*Arthrobotrys oligospora*	Artol3057	[illegible]
			Artol52763	[illegible]
IX	Eurotiomycetes	*Aspergillus aculeatus*	Aspacu210310	[illegible]
			Aspacu423845	[illegible]
			Aspacu475574	[illegible]
			Aspacu141510	[illegible]
			Aspacu422223	[illegible]
			Aspacu63958	[illegible]
			Aspacu64679	[illegible]
			Aspacu44287	[illegible]
X	Dothideomycetes	*Periconia macrospinosa*	Perma535873	[illegible]
			Perma579807	[illegible]
			Perma235665	[illegible]
			Perma510285	[illegible]
			Perma428062	[illegible]
			Perma516073	[illegible]
			Perma472447	[illegible]
			Perma527231	[illegible]
			Perma654005	[illegible]
XII	Lecanoromycetes	*Usnea florida*	Usnfl376191	[illegible]
			Usnfl852003	[illegible]
			Usnfl605016	[illegible]
			Usnfl891003	[illegible]
			Usnfl830025	[illegible]
			Usnfl941042	[illegible]
XIII	Leotiomycetes	*Cadophora malorum*	Cadma1166	[illegible]
			Cadma5464	[illegible]
			Cadma14331	[illegible]
			Cadma15598	[illegible]
			Cadma8765	[illegible]
			Cadma5119	[illegible]
			Cadma4624	[illegible]
			Cadma10444	[illegible]
			Cadma13479	[illegible]
			Cadma14158	[illegible]
			Cadma13991	[illegible]
			Cadma175	[illegible]
			Cadma255	[illegible]
XIV	Sordariomycetes	*Trichoderma atroviride*	Triat283564	[illegible]
			Triat90169	[illegible]
			Triat39327	[illegible]
			Triat31598	[illegible]
			Triat43816	[illegible]
			Triat6990	[illegible]
			Triat319992	[illegible]
XV	Xylonomycetes	*Xylona heveae*	Xylhe266999	[illegible]
			Xylhe198721	[illegible]
			Xylhe215125	[illegible]
			Xylhe044	[illegible]
			Xylhe054	[illegible]
XVI	Saccharomycotina	*Lipomyces orientalis*	Lipor337367	[illegible]
			Lipor326481	[illegible]
			Lipor334472	[illegible]
			Lipor167918	[illegible]
			Lipor342385	[illegible]
XVII	Taphrinomycotina	*Taphrina populi-salicis*	Tappo1089904	[illegible]
			Tappo1300026	[illegible]
			Tappo1300025	[illegible]
			Tappo1277613	[illegible]
			Tappo1277612	[illegible]
			Tappo1302952	[illegible]
			Tappo1303368	[illegible]
			Tappo1298756	[illegible]
XVIII	Glomeromycotina	*Geosiphon pyriformis*	Geopy171	[illegible]
			Geopy098	[illegible]
			Geopy1701	[illegible]
			Geopy1482	[illegible]
XIX	Mortierellomycotina	*Mortierella alpina*	Moral1948	[illegible]
			Moal10232	[illegible]
			Moral4868	[illegible]
			Moral7508	[illegible]
			Moral10332	[illegible]
XX	Mucoromycotina	*Rhizomucor pusillus*	Rhipu681	[illegible]
			Rhipu6272	[illegible]
XXI	Zoopagomycotina	*Cunninghamella_echinulata*	Cunech241350	[illegible]
			Cunech285912	[illegible]
			Cunech258863	[illegible]
			Acate1000072	[illegible]
XXII	Entomophthoromycotina	*Basidiobolus meristosporus*	Basme033	[illegible]
			Basme565	[illegible]
			Basme50562	[illegible]
			Basme129234	[illegible]
			Basme223097	[illegible]
			Basme231814	[illegible]
			Basme233018	[illegible]
			Basme238666	[illegible]
			Basme307273	[illegible]
			Basme326148	[illegible]
			Basme328970	[illegible]
			Basme333109	[illegible]
			Basme368551	[illegible]
			Basme207572	[illegible]
			Basme207752	[illegible]
			Basme214444	[illegible]
			Basme231096	[illegible]
			Basme234148	[illegible]
			Basme247457	[illegible]
			Basme247587	[illegible]
			Basme289817	[illegible]
			Basme318284	[illegible]
			Basme374593	[illegible]
XXIII	Kickxellomycotina	*Coemansia mojavensis*	Coemoj472274	[illegible]
			Coemoj499325	[illegible]
			Coemoj504042	[illegible]
			Coemoj504067	[illegible]
			Coemoj467721	[illegible]
XXIV	Blastocladiomycota	*Allomyces macrogynus*	Allma37	[illegible]
			Allma5232	[illegible]
			Allma6889	[illegible]
			Allma7110	[illegible]
			Allma8110	[illegible]
			Allma10988	[illegible]
			Allma12960	[illegible]
			Allma12961	[illegible]
			Allma2905	[illegible]
			Allma16284	[illegible]
XXV	Chytridiomycetes	*Globomyces pollinis-pini*	Glopol625454	[illegible]
			Glopol119922	[illegible]
			Glopo539730	[illegible]
XXVI	Monoblepharidomycetes	*Hyaloraphidium curvatum*	Hyacur714381	[illegible]
			Hyacur613526	[illegible]
			Hyacur661765	[illegible]
			Hyacur673571	[illegible]
			Hyacur685027	[illegible]
			Hyacur714401	[illegible]
			Hyacur423580	[illegible]
XXVII	Neocallimastigomycetes	*Neocallimastix californiae*	Neoca195738	[illegible]
			Neoca702287	[illegible]
			Neoca708696	[illegible]
XXVIII	Microsporidia	*Ordospora pajunii*	Ordpa4284	[illegible]
XXIX	Cryptomycota	*Paramicrosporidium saccamoebae*	Parsa3432	[illegible]
XXX	Aphelidiomycota	*Amoeboaphelidium protococcarum*	Amopr8850	[illegible]
