# Supplementary figures and images for: Major Intrinsic Proteins in Fungi: A Special Emphasis on the XIP Subfamily
Source: J Fungi (Basel). 2025 Jul 21;11(7):543. doi: 10.3390/jof11070543 (PMC12300952; doi:10.3390/jof11070543)

Tree scale: 1

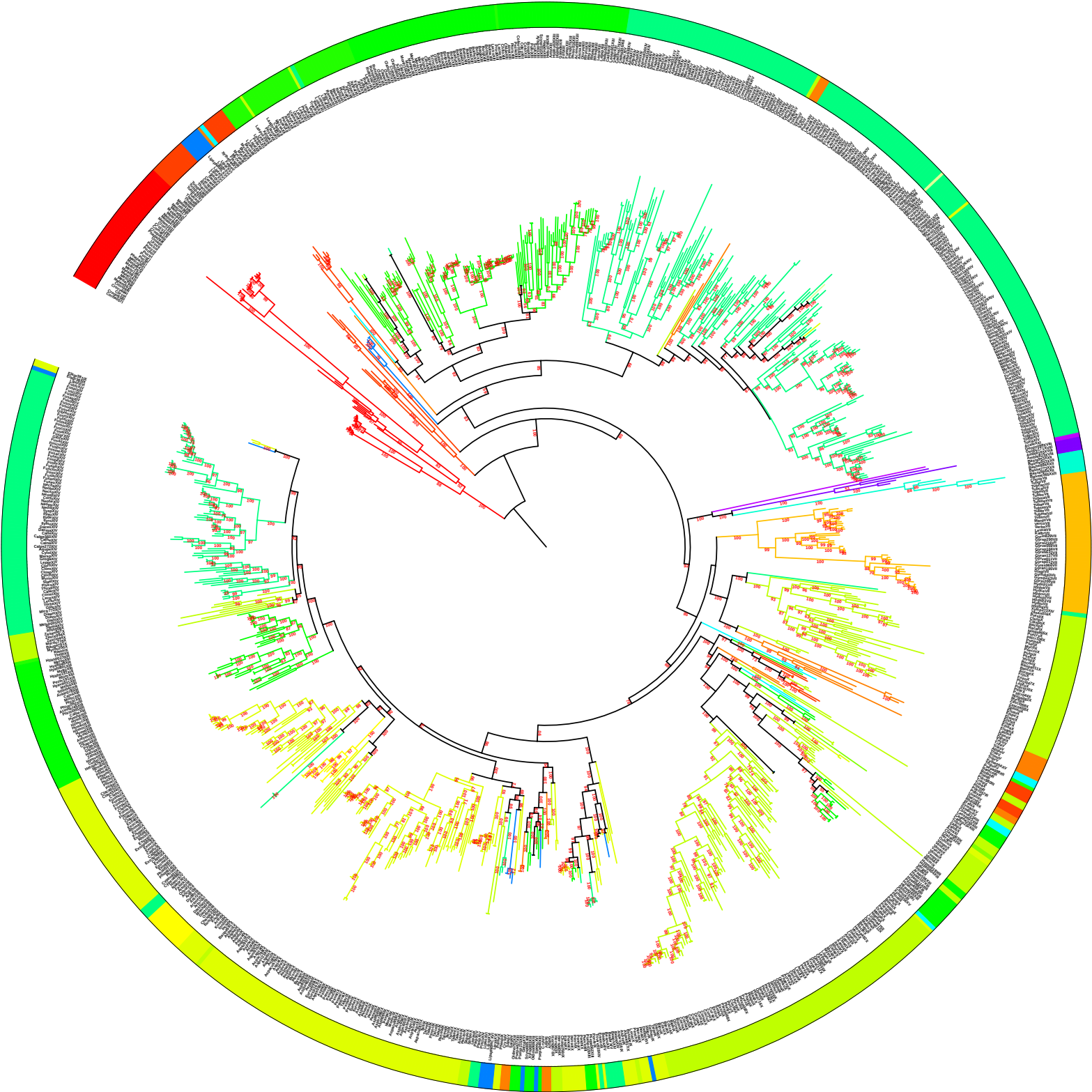

Supplement: Supplementary file 1 [file jof-11-00543-s001.zip › jof-3752183_Supplementary_Figure_S3.pdf]
